# A FAK Inhibitor Boosts Anti-PD1 Immunotherapy in a Hepatocellular Carcinoma Mouse Model

**DOI:** 10.3389/fphar.2021.820446

**Published:** 2022-01-18

**Authors:** Yuhua Wei, Yufeng Wang, Nanbin Liu, Ran Qi, Yan Xu, Kun Li, Yu Feng, Baomin Shi

**Affiliations:** ^1^ Department of General Surgery, Tongji Hospital, Tongji University Medical School, Shanghai, China; ^2^ Shuguang Hospital, Shanghai University of Traditional Chinese Medicine, Shanghai, China

**Keywords:** hepatocellular carcinoma, FAK inhibitor (VS4718), PD1, PD-L1, combination

## Abstract

Anti-PD-1/PD-L1 immunotherapy has limited efficacy in hepatocellular carcinoma (HCC) and does not benefit all patients. A FAK inhibitor (VS-4718) has been reported to improve the microenvironment in some tumors. This study aimed to investigate the effect of the combination of the FAK inhibitor VS4718 and anti-PD1 for the treatment of HCC in a mouse model and its possible mechanism of action. The expression of FAK and infiltrated immune cells in human HCC from the data of TCGA were analyzed. A primary murine HCC model was established *via* protooncogene (c-Met/β-catenin) transfection. The pathological characteristics of tumors were examined after the mice were treated with VS4718 and/or anti-PD1 therapy. This study revealed that FAK is highly expressed in human HCC and is associated with poor prognosis of OS (overall survival) and PFS (progress free survival) in HCC patients. Immune cell infiltration (CD8^+^ T, Tregs, M0, M2, CAFs and MDSCs) was correlated with FAK expression. In the experimental HCC model, the combination of a FAK inhibitor VS4718 and an anti-PD1 antibody had a better effect than monotherapy against HCC. VS4718 reduced the number of Tregs and macrophages but increased the number of CD8^+^ T cells in HCC mice. Notably, FAK inhibitor promoted the expression of PD-L1 in HCC. This study suggested that combination of the FAK inhibitor VS4718 and anti-PD1 could be a potential therapy for HCC by improving the immune environment, reducing liver fibrosis and simultaneously preventing PD1 from binding to the increased PD-L1 induced by FAK inhibitor VS4718.

## Introduction

Hepatocellular carcinoma (HCC) is one of the leading causes of cancer-related death (Yang et al., 2019). Due to the high rate of recurrence and metastasis of HCC and resistance to antitumor drugs, the 5-years survival rate of HCC patients is low (Greten et al., 2019; Xiang et al., 2019). Therefore, exploration of more effective treatments for HCC is an urgent need (Rimassa et al., 2019).

In recent years, attention has been given to the effectiveness of immunotherapy which targets immune checkpoints, such as anti-programmed cell death 1(PD1) targeted immunotherapy, which has resulted in encouraging effects in the treatment of some solid tumors (Motzer et al., 2020). PD1 belongs to the CD28 family and it regulates peripheral immune tolerance and autoimmunity in CD8^+^ T cells, regulatory T cells (Tregs), and myeloid suppressor cells (MDSCs) (Yao et al., 2018; Pu et al., 2019). PD-L1 and PD-L2 are specific ligands of PD-1 and are mainly expressed in tumor cells and antigen-presenting cells. PD1 inhibits the function of effector T cells when it binds to PD-L1 or PD-L2 (Dong et al., 1999). The FDA has approved the anti-PD1 drugs nivolumab and pembrolizumab for the second-line treatment of HCC. However, not all patients are sensitive to these therapies, and the clinical efficacy of anti-PD1 is limited to a subset of patients, with a total effective rate of 20% or lower (Xu et al., 2018; Zhu et al., 2018). These results indicate that most patients are not suitable for anti-PD1 therapy. Therefore, it is of great significance to find ways to improve the sensitivity and effectiveness of anti-PD1 therapy.

Focal adhesion kinase (FAK) is encoded by the PTK2 gene. It is a nonreceptor tyrosine kinase in the integrin signal transduction cascade, which mediates the connection between cells and extracellular matrix (Evans and Müller, 2000). Highexpression of FAK is detected in a variety of human solid tumors (Song et al., 2021; Torres-Ayuso, et al., 2021). FAK can be phosphorylated and activated to target multiple downstream signaling pathways, promoting cell growth, development, invasion, and metastasis. FAK affects both cancer cells and tumor stromal cells (Shang et al., 2015; Shang et al., 2016; Lees et al., 2021). FAK can regulate the transcription of inflammatory genes and promote antitumor immune evasion (Jeong, et al., 2021). Previous studies have found that inhibition of FAK activity changed immune cell infiltration in tumor microenvironment (Jiang et al., 2016; Serrels et al., 2017; Canel et al., 2020). A FAK inhibitor VS4718, also named PND-1186, blocks FAK Tyr-397 phosphorylation and has become a potential anticancer drug (Wang et al., 2019; Dawson et al., 2021). The effect of VS4718 with PD1 blockade as a possible combination therapy has not been evaluated.

In this work, we used the c-Met/β-catenin plasmids to induce primary HCC model in C57BL/6 J mice. Using this mouse primary HCC model, we aimed to observe the efficacy of a FAK inhibitor (VS4718) in combination with an anti-PD1 antibody for the treatment of HCC and investigate the related mechanism.

## Materials and Methods

### Data Source and Preprocessing

Gene expression data of LIHC projects (included 50 normal and 374 tumor tissues) with clinical information were obtained from TCGA (https://portal.gdc.cancer.gov/). Survival analyses, such as overall survival (OS), were measured from the date of study enrollment to death from any cause or last follow-up. Disease-free survival (DFS) is defined as the time between the treatment of intrahepatic lesions and the first discovery of recurrence or metastasis (Liu et al., 2018). The relationship between the expression of FAK and immune cells (CD8^+^ T, Tregs, M0, M2, CAFs, and MDSCs) was analyzed by TIMER2.0 website (http://timer.cistrome.org/) (Li et al., 2016; Li et al., 2017; Li et al., 2020).

### Plasmids

The plasmids pT3-EF1a-c-Met (Cat. #31784) and pT3-N90-β-catenin (Cat. #31785) were obtained from Addgene (United States). The plasmids pCMV/SB (Liang et al., 2018) was presented by Professor Dong (Naval Medical University, Shanghai, China). The plasmids were purified using EndoFree Maxi Plasmid Kit (Cat. #DP117) from Tiangen Biotech (China) for hydrodynamic tail vein injection.

### Mice and Treatments

C57BL/6 J mice were purchased from Jiesijie (Shanghai, China). Mice were 6–8 weeks old and their body weight ranged from 18 to 22 g. The mice were placed in a micro-isolator cage in a room illuminated from 7:00 AM to 7:00 PM (12:12-HR light-dark cycle) and adequate food and water were provided. All animal experimental procedures were approved by the Institutional Animal Care and Use Committee of Tongji University.

To establish an HCC mouse model, we used the tail vein hydrodynamic high-pressure injection technique to inject 22.5 µg pT3-EF1α-c-MET; 22.5 µg pT3-EF1α-∆N90-β-catenin; 5 µg pCMV/SB plasmid DNA dissolved in sterile saline (10% of the body weight of mice) into the mouse. After 4 weeks, the HCC mouse model was established which caused cancer only in liver (Shang et al., 2015).

The HCC mice were treated with drugs as following: 1) placebo group: A placebo is given orally (0.5% methylcellulose) or by injection (PBS) in equal doses and the same number of times. 2) anti-PD1 group: 200 μg PD1 antibody (anti-mPD1 clone RMP1-14, BioXcell, Cat. # BE0146) was injected by intraperitoneal injection, once every 3 days; 3) FAK inhibitor (VS4718) group: 50 mg/kg FAK inhibitor (VS4718) (Csnpharm, Cat. # CSN16593) dissolved in 0.5% methylcellulose (v/v, saline) was given to mice by gavage, twice a day. 4) The combination of anti-PD1 and FAK inhibitor group: 200 μg PD1 antibody was injected by intraperitoneal injection, once every 3 days and 50 mg/kg FAK inhibitor (VS4718) dissolved in 0.5% methylcellulose (v/v, saline) was given to mice by gavage, twice a day.

### Protein Extraction and Western Blot

The mouse HCC tissues were added with RIPA (Thermo Scientific, 89,900) containing protease inhibitors (MCE, HY-K0010), placed on ice, and lysed for 30 min. After centrifugation at 12,000 rpm at 4°C for 10 min, the supernatant was transferred to a new EP tube. Then, the protein samples were quantified by the BCA method. The samples were placed in a metal bath and denatured at 100°C for 10 min. The protein samples were separated by electrophoresis on 10% SDS-PAGE gel and transferred to a 0.45 mm nitrocellulose membrane. Western blotting was performed with specific primary antibodies. Finally, imaging was performed using ECL (GE Health Care, United States). The detailed information about antibodies was listed in Supplementary Table S1.

### ImmunoHistochemical Staining

The mouse HCC tissue was immobilized in 10% formalin and embedded in paraffin. Then, 5 μm thick slices were dewaxed in xylene and rehydrated in descending graded ethanol. A specific antigenic repair solution was used for antigenic repair. The slices were sealed in 10% BSA solution at room temperature for 1 h. The slides were then incubated overnight with specific antibodies at 4°C.The corresponding positive expression was detected by 3,3′ -diaminobenzidine (DAB) or indirect immunofluorescence. Positive staining was scored in at least three fields. At least three mice were included in each group.

### RNA Extraction, cDNA Synthesis, and qPCR

The total RNAs were extracted from cells and tissues by the total RNA rapid extraction kit(Bioteke Corporation, RP4002). The cDNA was reverse-transcribed from 500 ng of total RNA using HiScript II Q RT SuperMix for qPCR kit (Vazyme, R222-01). The cDNA was diluted (1:20) for qPCR by ChamQ SYBR qPCR Master Mix (Vazyme, Q311-02) with gene-specific primers. β-actin was used as an endogenous control for normalization. The detailed information about the primer sequences was listed in Supplementary Table S2.

### Statistical Analysis

GraphPad Prism 8.0.2 software was used for statistical analysis. Data were expressed as mean ± standard deviation (SD). Student’s *t*-test was used to compare two groups. Multiple groups were compared using one-way ANOVA. *p* < 0.05 was considered to be statistically significant. A Fishers’ exact test was used to analyze significance. The means ± SD are shown in the figures where applicable.

## Results

### High Expression of FAK in Human HCC was Associated With Poor Prognosis and an Immunosuppressive TME

**FIGURE 1:**
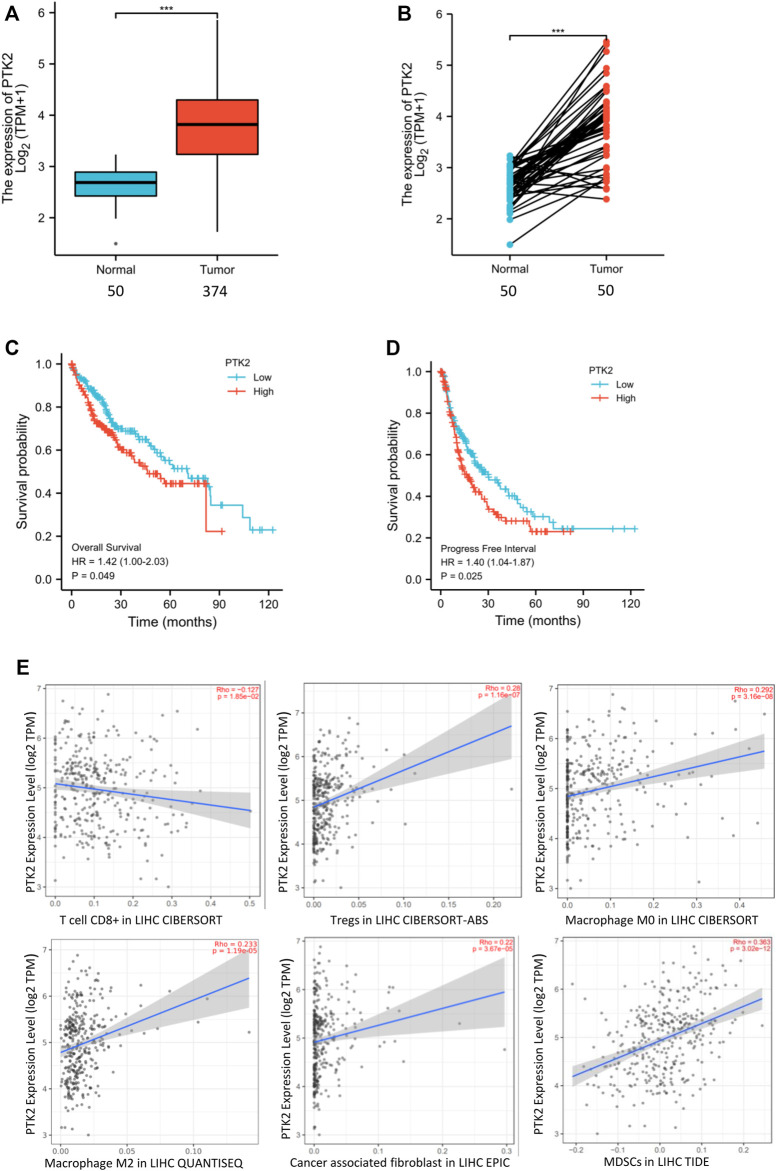
High expression of FAK in human HCC is associated with poor prognosis and an immunosuppressive TME **(A)** FAK expression is higher in human HCC tissues than in paired normal tissues by Wilcoxon rank sum test (*p* < 0.001). **(B)** The expression of FAK significantly increased in HCC tissues compared with adjacent tissues in the paired samples with Wilcoxon signed-rank test. **(C–D)** Kaplan-Meier survival analysis showed that increased expression of FAK was significantly associated with poor Overall Survival (*p* = 0.049) and Progression-free survival (*p* = 0.027). **(E)** The relationship between FAK expression and CD8^+^ T cells(Rho = -0.17 *p* = 1.85e-2), Tregs(Rho = 0.28 *p* = 1.16e-7),M0(Rho = 0.292; *p* = 3.16e-8),M2(Rho = 0.233; *p* = 1.19e-5),CAFs(Rho = 0.22; *p* = 3.67e-5) and MDSCs(Rho = 0.363; *p* = 3.02e-12).Significance identification: ns, *p* ≥ 0.05; *, *p* < 0.05; **, *p* < 0.01; ***, *p* < 0.001.

We analyzed the expression of FAK in human HCC and the correlation of FAK with patient outcome. It was found that FAK was highly expressed in HCC tissues (*p* < 0.001) (Figure 1A). In addition, we investigated FAK expression in the paired sample, and the expression of FAK was also higher in HCC tissues (*p* < 0.001) (Figure 1B). Cox regression analysis showed that high expression of FAK was associated with poor OS (*p* = 0.049) and PFS (*p* = 0.027), indicating that HCC patients with high FAK expression had a worse prognosis than those with low FAK expression (Figures 1C,D).

To evaluate whether FAK might impact the tumor microenvironment (TME), we analyzed the relationship between the expression of FAK and immune cells in HCC tumor tissues by TIMER2.0. The results showed that the expression of FAK was correlated with the number of CD8^+^ T, Tregs, macrophages (M0, M2), CAFs and MDSCs (Figure 1E).

### Combination of FAK Inhibition and Anti-PD1 Therapy Effectively Inhibited the Growth of HCC in Mice

**FIGURE 2:**
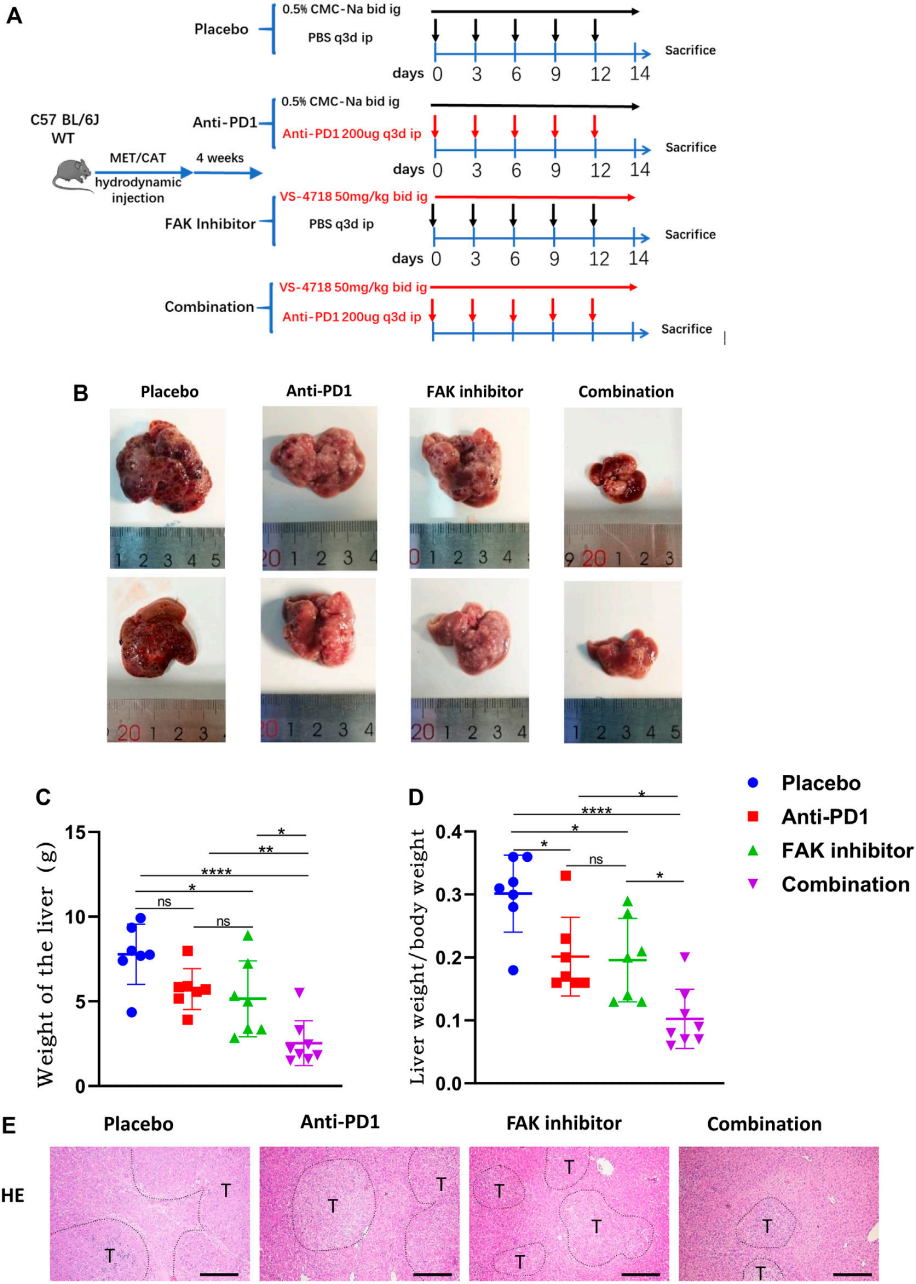
Combination of FAK inhibition and anti-PD1 therapy effectively inhibited the growth of HCC in mice **(A)** After 4 weeks of plasmid injection in C57BL/6 J mice, a primary hepatocellular carcinoma model was established and the mice were randomly divided into four groups (Placebo group, *n* = 7; Anti-PD1 group, *n* = 7; FAK inhibitor group, *n* = 7; Combination group, *n* = 8), and specific information about the administration (time, dosage, and method). **(B)** The mouse liver after 2 weeks of medication. **(C–D)** The liver weight of mice and the liver weight/body weight of mice were compared in each group (Placebo group, *n* = 7; Anti-PD1 group, *n* = 7; FAK inhibitor group, *n* = 7; Combination group, *n* = 8). Significance identification: ns, *p* ≥ 0.05; *, *p* < 0.05; **, *p* < 0.01; ***, *p* < 0.001. **(E)** HCC tissues of mice were histologically analyzed by H and E staining (scale bars, 400 μm).

To observe the efficacy of VS4718, anti-PD1 monotherapy or the combination of VS4718 and anti-PD1 in the treatment of HCC, we established a C57BL/6 J primary HCC model with complete immune function and then randomly grouped them for drug administration (Figure 2A). Compared with placebo, VS4718 or anti-PD1 monotherapy, the combination of VS4718 and anti-PD1 significantly inhibited HCC development in mice (Figure 2B). Compared with placebo and monotherapy, both liver weight and liver weight/mouse body weight were significantly lower in the combination treatment group (Figures 2C,D). It was found that the tumors were smaller and the liver tissue structure was relatively normal on H and E staining in the combination treatment group compared to the placebo and monotherapy groups (Figure 2E). These results suggest that a FAK inhibitor (VS4718) can promote the anti-PD1 immunotherapeutic efficacy in HCC in mice.

### Combination of FAK Inhibition and Anti-PD1 Therapy Inhibited Proliferation and Promoted Apoptosis of HCC in Mice

**FIGURE 3:**
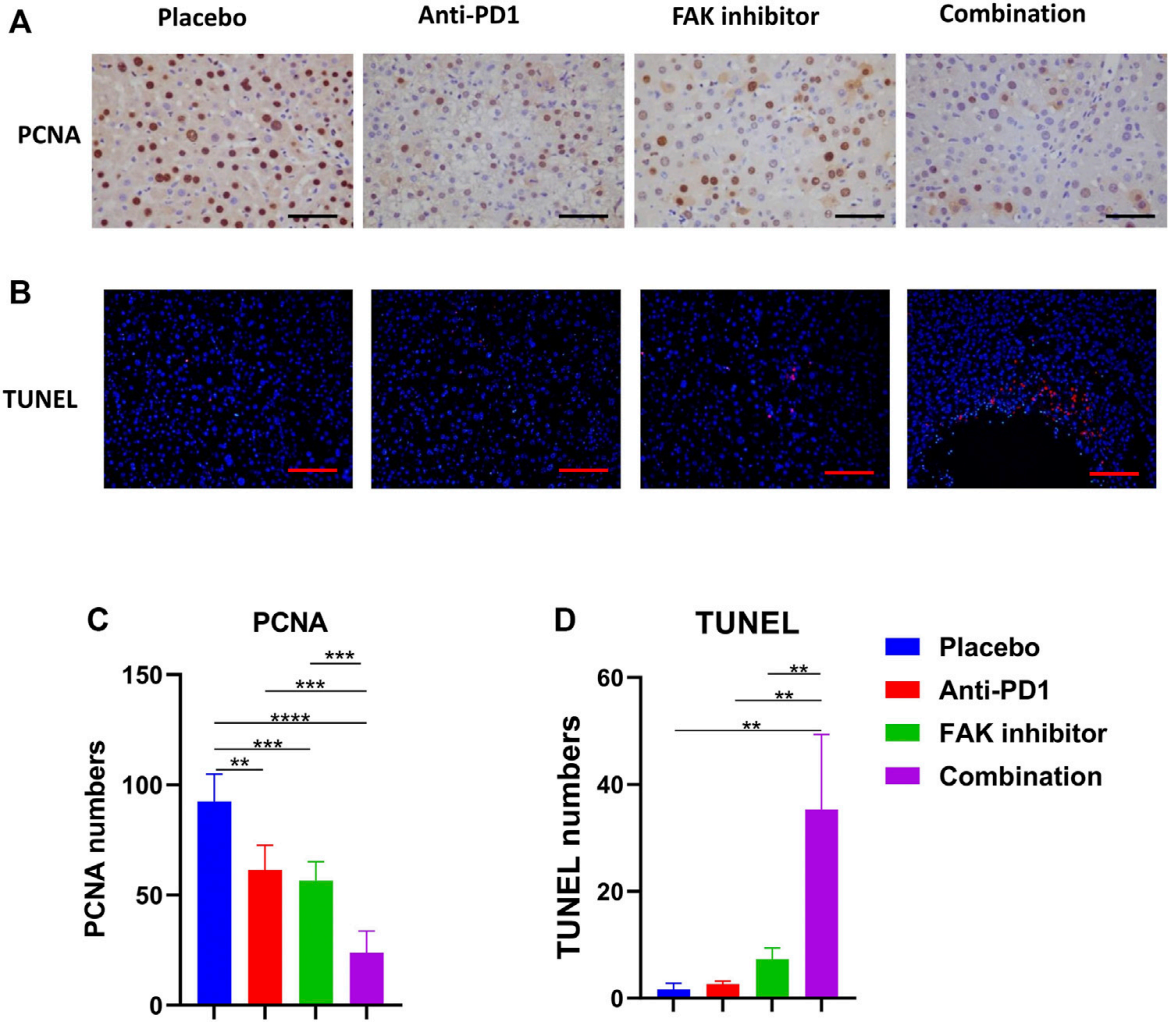
Combination of FAK inhibition and anti-PD1 therapy inhibited proliferation and promoted apoptosis of HCC in mice **(A)** PCNA immunohistochemistry on HCC tissues of mice (scale bars, 100 μm). **(B)** HCC tissues of mice in each group were stained with TUNEL-staining (scale bars, 200 μm). **(C)** Quantification of PCNA staining (*n* = 5 mice/group). **(D)** Quantification of TUNEL staining (*n* = 3 mice/group). Significance identification: ns, *p* ≥ 0.05; *, *p* < 0.05; **, *p* < 0.01; ***, *p* < 0.001.

To observe the effect of treatment on tumor status, including proliferation and apoptosis, PCNA immunohistochemical staining (Figure 3A) and TUNEL staining (Figure 3B) were performed. We analyzed the positive staining area in each group. We found that the combination treatment significantly inhibited tumor proliferation (Figure 3C) and promoted liver tumor cell apoptosis in mice compared with placebo and monotherapy (Figure 3D).

### FAK Inhibition Reduced the Fibrosis of HCC in Mice

**FIGURE 4:**
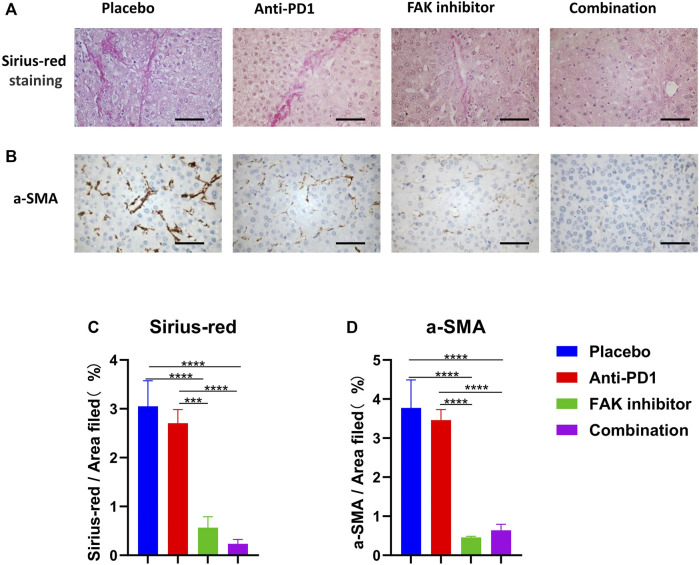
FAK inhibition reduced the fibrosis of HCC in mice **(A)** Sirius-red staining was performed on HCC tissues of mice in each group (scale bars, 100 μm). **(B)** a-SMA immunohistochemistry was performed on HCC tissues of mice in each group (scale bars, 100 μm). **(C)** Quantification of Sirius-red staining (*n* = 3 mice/group). **(D)** Quantification of a-SMA staining (*n* = 3 mice/group). Significance identification: ns, *p* ≥ 0.05; *, *p* < 0.05; **, *p* < 0.01; ***, *p* < 0.001.

To determine the effect of drug treatments on fibrosis of HCC in mice, Sirius red staining (Figure 4A) and a-SMA immunohistochemical staining (Figure 4B) were performed. The anti-PD1 group showed a similar level of fibrosis compared with the placebo group. The FAK inhibitor group and combination group showed a significantly lower level of fibrosis (Figures 4C,D), implying that inhibition of FAK could reduce fibrosis in HCC mice.

### FAK Inhibition Improved the Immune Microenvironment of HCC in Mice

**FIGURE 5:**
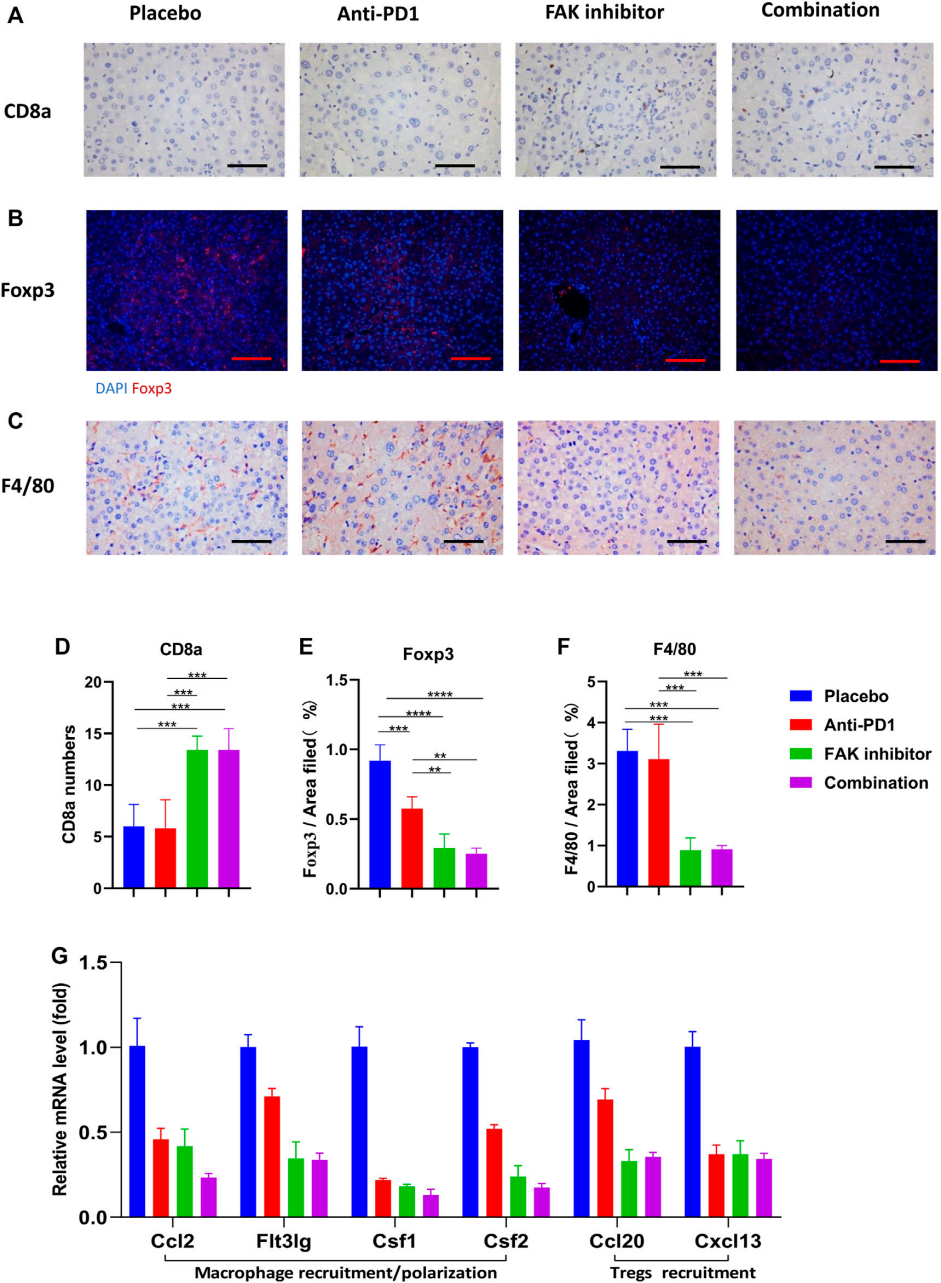
FAK inhibition improved the immune microenvironment of HCC in mice **(A)** CD8a immunohistochemistry on HCC tissues of mice in each group (scale bars, 100 μm). **(B)** Foxp3 immunofluorescence on HCC tissues of mice in each group (scale bars, 200 μm). **(C)** F4/80 immunohistochemistry on HCC tissues of mice in each group (scale bars, 100 μm). **(D)** The number of CD8a positive cells in on HCC of mice (*n* = 5 mice/group). **(E)** Quantitative analysis of Foxp3 positive area per field by ImageJ (*n* = 4 mice/group). **(F)** Quantitative analysis of F4/80 positive area per field by ImageJ (*n* = 4 mice/group). Significance identification: ns, *p* ≥ 0.05; *, *p* < 0.05; **, *p* < 0.01; ***, *p* < 0.001. **(G)** The mRNA expressions of macrophage recruitment/polarization factors (Ccl2, Flt3lg, Csf1, Csf2) and Tregs recruitment factors (Ccl20, Cxcl13) in HCC tissues of mice detected by q-PCR (*n* = 3 mice/group).

To detect immune cell infiltration in HCC tissues of mice, CD8a, Foxp3, and F4/80 + immunohistochemical staining were used (Figures 5A–C). We found that the level of CD8a infiltration in HCC was higher in the FAK inhibitor monotherapy and combination groups than in the placebo or anti-PD1 groups (Figure 5D). Foxp3, a surface marker of Tregs, was significantly decreased (Figure 5E). F4/80 + expression decreased significantly (Figure 5F). These results suggest that a FAK inhibitor alone or in combination with an anti-PD1 antibody increases the number of CD8a T cells and decreases the number of Tregs and macrophages. We also detected the mRNA expression of some macrophage recruitment molecules (Ccl2, Flt3lg, Csf1, Csf2) and Tregs recruitment molecules (Ccl20, Cxcl13) in HCC tissues. The results showed that, compared with placebo and anti-PD1 monotherapy, a significantly lower expression of macrophage recruitment molecules and Tregs recruitment molecules was observed in the FAK inhibitor monotherapy and combination groups (Figure 5G).

### FAK Inhibition Increased the Expression of PD-L1 in HCC

**FIGURE 6:**
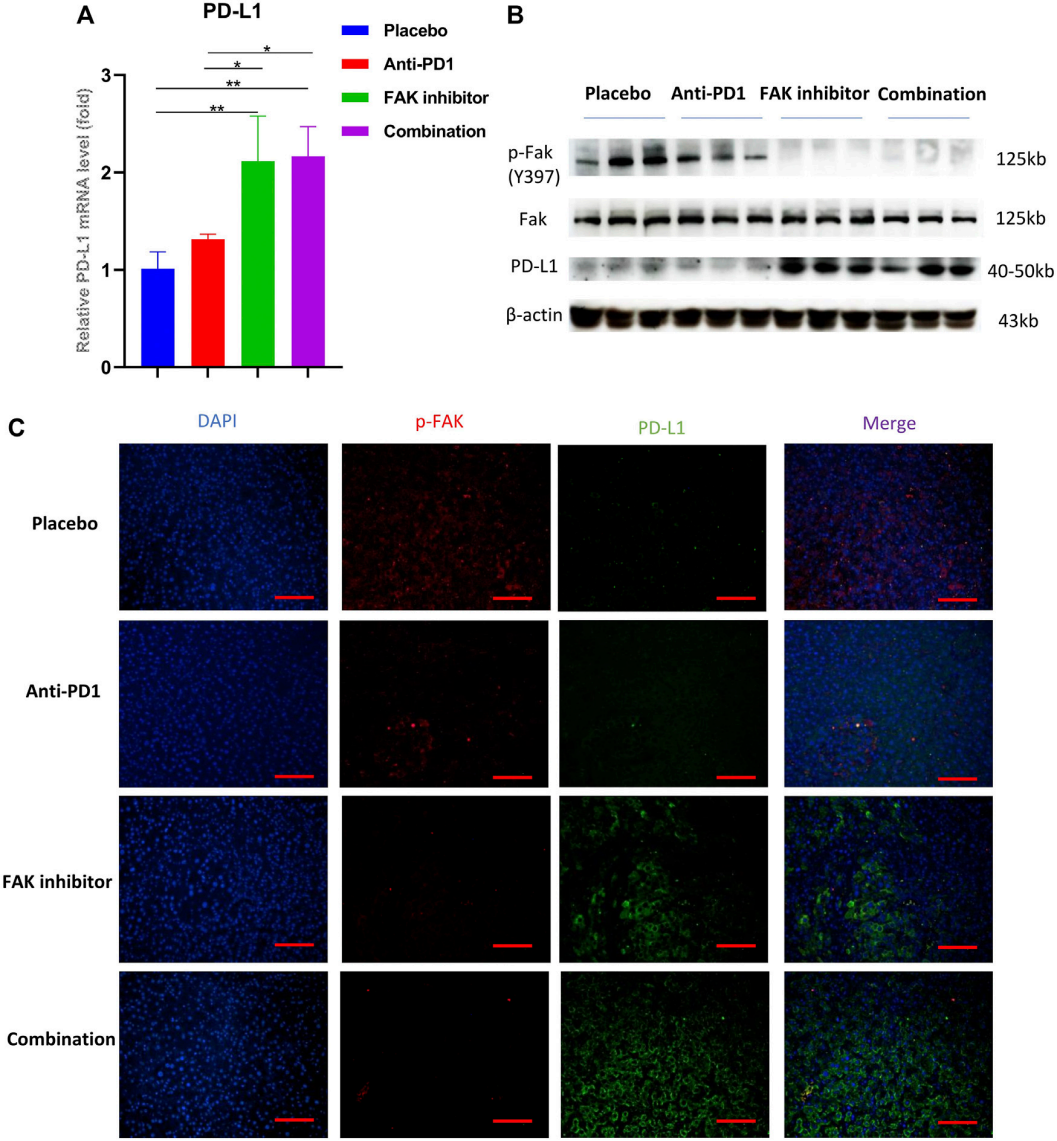
FAK inhibition increased the expression of PD-L1 in HCC **(A)** The mRNA expressions of PD-L1 in HCC in mice detected by q-PCR (*n* = 3 mice/group). Significance identification: ns, *p* ≥ 0.05; *, *p* < 0.05; **, *p* < 0.01; ***, *p* < 0.001. **(B)** The expression levels of *p*-Fak (Y397), Fak and PD-L1 in HCC in mice detected by Western Blotting (*n* = 3 mice/group). **(C)** Immunofluorescence staining of *p*-FAK and PD-L1 in HCC in mice (scale bar, 200 μm).

To study whether the FAK inhibitor can affect PD-L1 expression in HCC in mice, we used qPCR to detect the mRNA expression of PD-L1. The results showed that the mRNA expression of PD-L1 in the FAK inhibitor monotherapy group and the combination group was higher than that in the placebo and anti-PD1 monotherapy groups (Figure 6A). Then, we extracted proteins from HCC in mice to perform Western blotting. We found that compared with the placebo group and anti-PD1 monotherapy group, *p*-FAK protein expression was decreased, but PD-L1 protein expression was significantly increased in the FAK inhibitor monotherapy group and the combination group (Figure 6B). To verify the expression of *p*-FAK and PD-L1, immunofluorescence staining was performed on the HCC tissues of mice. We also found that *p*-FAK expression was decreased in both the FAK inhibitor monotherapy group and the combination group, but PD-L1 protein expression was significantly increased (Figure 6C).

## Discussion

In this study, we observed the effect of a FAK inhibitor (VS4718) in combination with an anti-PD1 antibody for the treatment of HCC in a mouse model. The results suggested that VS4718 significantly enhanced the sensitivity of HCC to anti-PD1 and improved therapeutic effect in mice.

FAK, which is overexpressed and highly phosphorylated in a variety of cancer cells, can activate multiple signaling pathways (Zhang et al., 2020). FAK not only affects cancer cells but also the TME which is associated with tumor growth and apoptosis (Anderson et al., 2017; Hamidi and Ivaska, 2018). The high expression of FAK is related to inhibitory immune cell infiltration in some tumors (Li et al., 2016; Li et al., 2017; Li et al., 2020). At present, many small molecule FAK inhibitors have been evaluated or are undergoing clinical trials, and results show that FAK inhibitors have anticancer efficacy and tolerability (Brown et al., 2018; de Jonge et al., 2019; Mohanty et al., 2020). Previous research showed that FAK knockdown or pharmacological inhibition of FAK activity promoted apoptosis and induced tumor regression (Haun et al., 2018; Cooper and Giancotti, 2019). We found that FAK was highly expressed in human HCC tissues and associated with poor prognosis of patients, which was consistent with the results of a clinical study (Fujii et al., 2004). Our experiment in mice showed that inhibition of the activity of FAK not only inhibited the growth of HCC but also reduced liver fibrosis and improved the immune microenvironment of HCC in mice.

Clinical trials showed that only 14.3% (22/154) of HCC patients responded to anti-PD1 therapy. (http://www.opdivohcp.com/advanced-hcc/efficacy/clinical-trial). Another challenge is that patients who respond initially may develop drug resistance later, leading to disease recurrence (Schoenfeld and Hellmann 2020). Therefore, some drugs combined with anti-PD1 are being explored to improve the sensitivity of HCC to treatment (Cheng et al., 2020). The immunosuppressive TME has been considered to be the reason for the failure of immunotherapy in HCC (Sangro et al., 2021). Extensive myeloid cell infiltration, such as macrophages and Tregs, may lead to dysfunction of infiltrating T cells (Mitchem et al., 2013), and cause tumor immunosuppression (Jiang, et al., 2016). These factors might contribute to the low sensitivity of tumors to anti-PD1 treatment. Similarly, in our experiments, anti-PD1 exhibited a weaker therapeutic effect on murine HCC, which is characterized by a higher degree of fibrosis and more immunosuppressive cell (macrophage and Treg) infiltration in the HCC tissues of mice. Some reports suggested that FAK inhibitors could act as immune modulators to improve the immune microenvironment of tumors (Jiang et al., 2016; Anderson et al., 2017; Osipov et al., 2019). Therefore, we evaluated the effect of FAK inhibitors on the liver tumor TME. A FAK inhibitor (VS4718) in combination with anti-PD1 therapy effectively inhibited the infiltration of macrophages and Tregs but increased CD8^+^ T cell infiltration in tumors compared to anti-PD1 monotherapy. Mechanistically, the effect of the FAK inhibitor on HCC immune infiltration may be due to a decrease in some macrophage recruitment molecules (Ccl2, Flt3lg, Csf1, Csf2) (Soncin et al., 2018; Sterner et al., 2019) and Treg cell recruitment molecules (Ccl20, Cxcl13) (Chen et al., 2017; Ji et al., 2020) in HCC.

Another reason for the inhibitory TME could be the highly fibrotic stroma of tumor tissue (Robinson et al., 2016). A high density of stroma forms a barrier that makes it difficult for drugs to reach the tumor interior (Provenzano et al., 2012). Previous reports showed that inhibition of FAK reduced tumor fibrosis, thereby reducing the tumor barrier and improving the TME (Jiang et al., 2016; Miller and Weissleder, 2017). Our results showed that a FAK inhibitor (VS4718) affects tumor fibrosis. We suggest that the antifibrotic effect of VS4718 plays a supporting role in the treatment of HCC. This effect might involve the inhibition of FAK on TGF-β/SMAD signaling pathway (Jiang et al., 2020).

Unexpectedly, we observed that treatment with VS4718 resulted in overexpression of PD-L1 in HCC. This finding differs from that of a previous study which showed that FAK inhibition induced the downregulation of PD-L1 in triple-negative breast cancer (Pan et al., 2019). High expression of PD-L1 may lead to the suppression of immune function (Topalian et al., 2016; Li et al., 2019). However, clinical studies have shown that patients with increased CD8^+^ T cell infiltration and high PD-L1 positivity in HCC are more sensitive to anti-PD1 therapy and have a significantly improved disease control rate, which is significantly associated with prolonged PFS and OS (Aguiar et al., 2018; Han et al., 2019; Morita et al., 2021). The combination of VS4718 and an anti-PD1 antibody can not only increase the infiltration of CD8^+^ T cells and reduce the infiltration of immunosuppressive Tregs and macrophages but also block the binding of PD1 on the surface of T cells to PD-L1 on the surface of tumor cells. This might be the main reason why the combination of a FAK inhibitor (VS4718) and an anti-PD1 antibody can better inhibit HCC in mice than FAK inhibitor (VS4718) monotherapy.

However, our murine model did not represent the heterogeneity of all HCC cases (Craig et al., 2020). The effect of a FAK inhibitor (VS4718) combined with an anti-PD1 antibody needs to be evaluated in different HCC models. The actual clinical therapeutic effect of FAK inhibitors combined with anti-PD1 antibodies on HCC patient needs further clinical research. In addition, the detailed mechanism of the combination of FAK inhibitor and anti-PD1 warrants further study.

In conclusion, our findings revealed that the combination of the FAK inhibitor VS4718 and an anti-PD1 antibody could suppress tumor progression in HCC mice and was better than monotherapy. The combined therapy improved the tumor immune microenvironment and reduced liver fibrosis. In addition, the combination therapy blocked the potential side effects of FAK inhibition-induced PD-L1 upregulation. Taken together, we demonstrate that the FAK inhibitor VS4718 enhances the efficacy of anti-PD1 immunotherapy in HCC. The combination of a FAK inhibitor and PD1 inhibitor could be a potential therapeutic strategy for the treatment of HCC.

## Data Availability

The original contributions presented in the study are included in the article/Supplementary Materials, further inquiries can be directed to the corresponding author.
