# Supplementary figures and images for: A FAK Inhibitor Boosts Anti-PD1 Immunotherapy in a Hepatocellular Carcinoma Mouse Model
Source: Front Pharmacol. 2022 Jan 18;12:820446. doi: 10.3389/fphar.2021.820446 (PMC8804348; doi:10.3389/fphar.2021.820446)

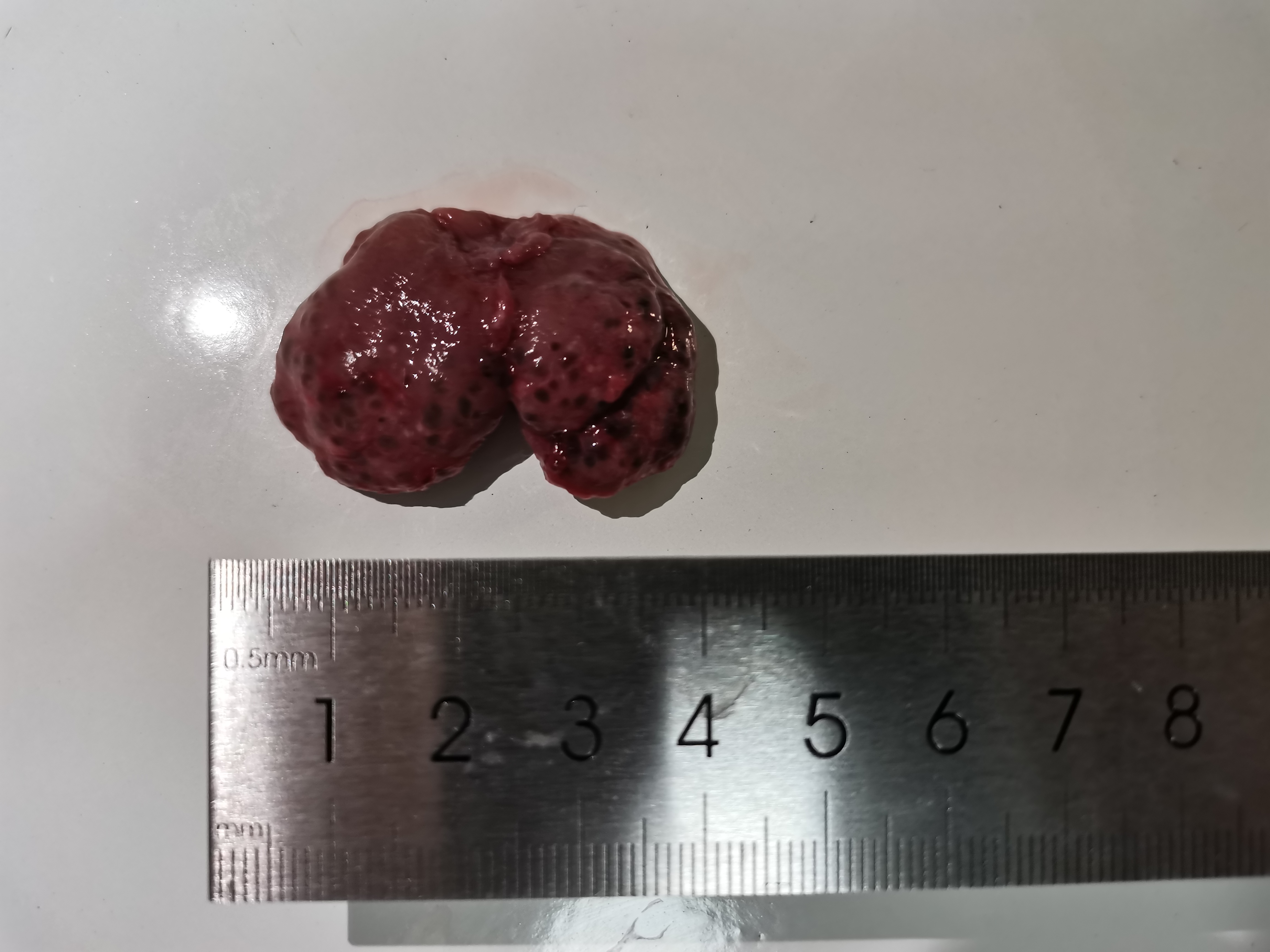

Supplement: Supplementary file 2 [file DataSheet8.ZIP › mouse liver of Placebo Group/Placebo1.jpg]

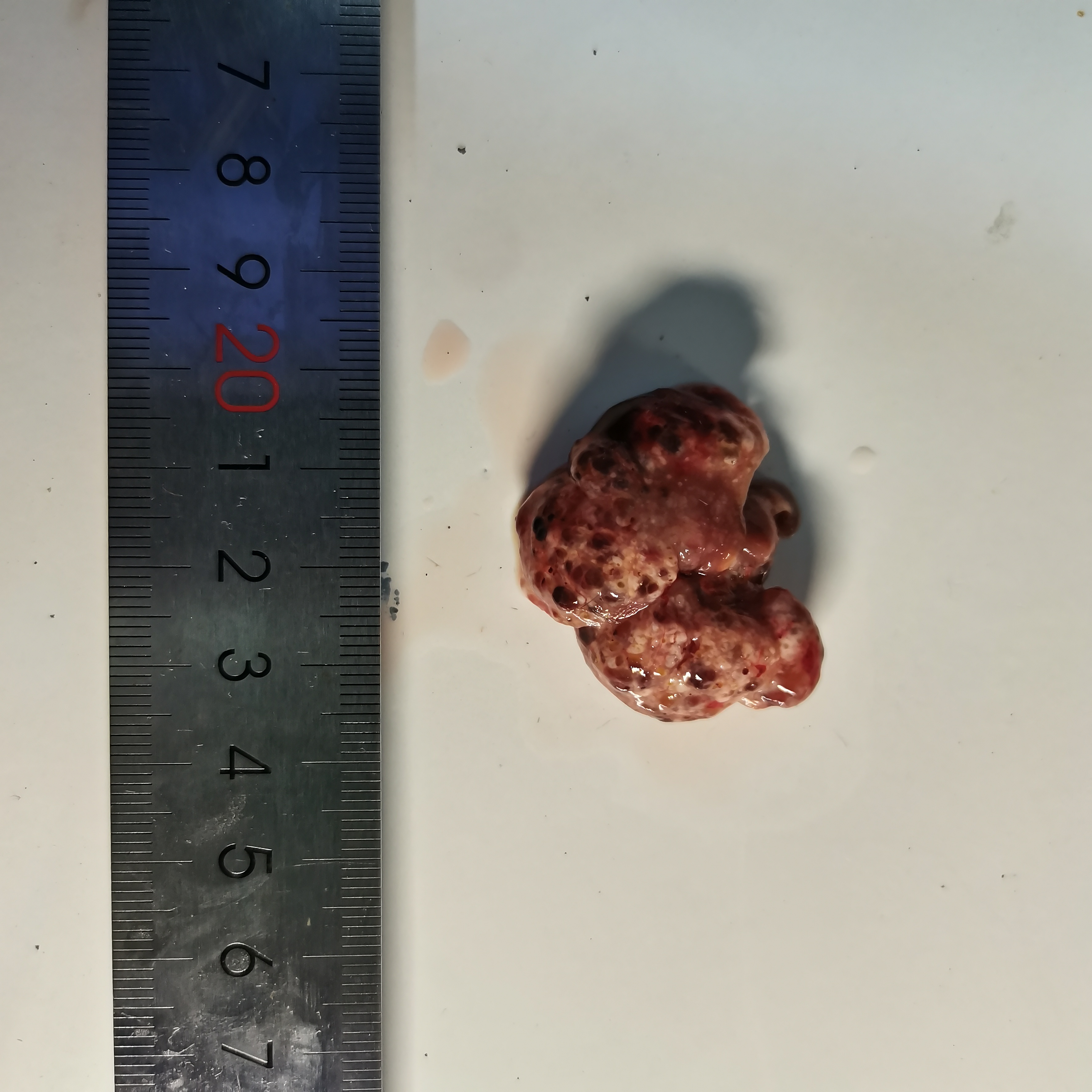

Supplement: Supplementary file 2 [file DataSheet8.ZIP › mouse liver of Placebo Group/Placebo2.jpg]

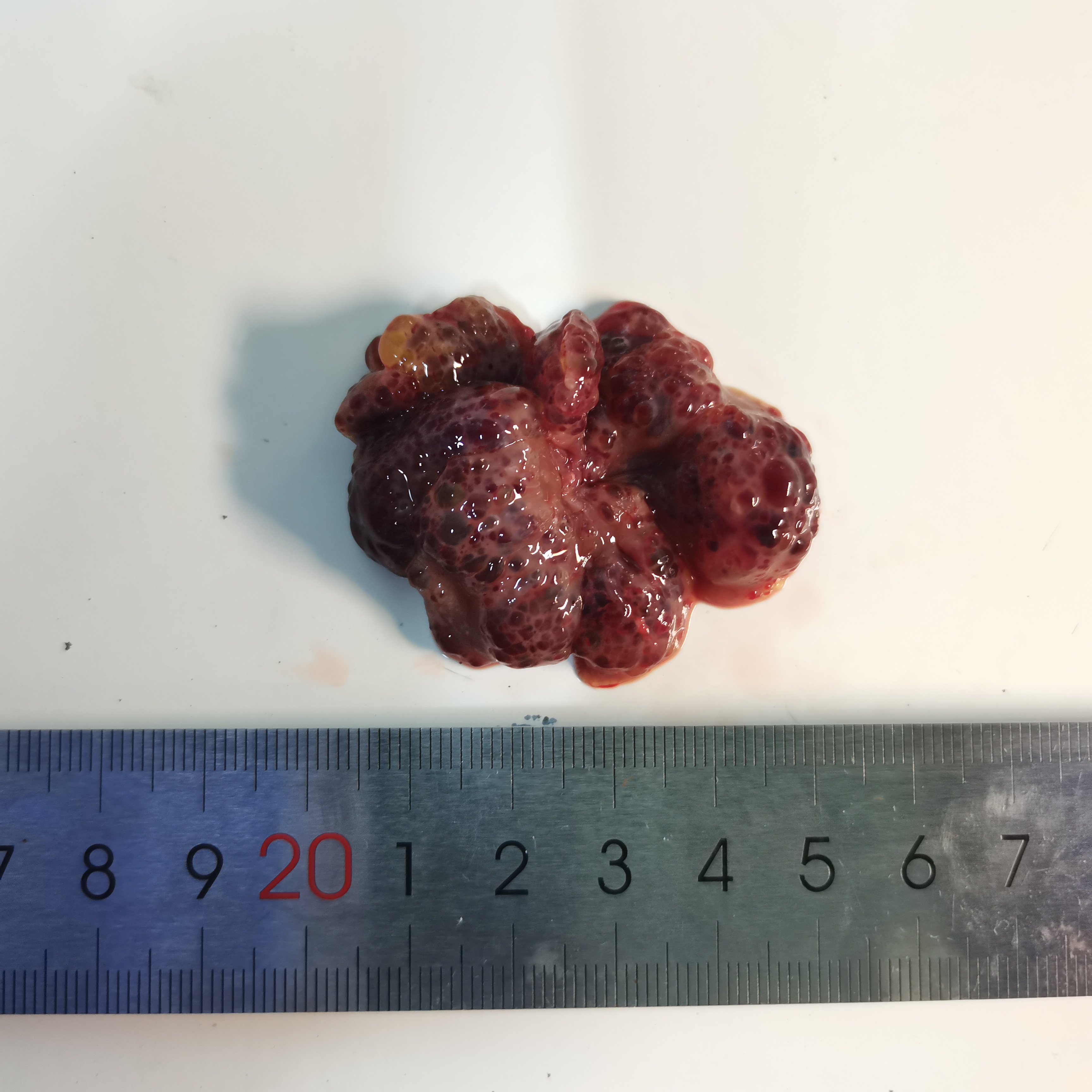

Supplement: Supplementary file 2 [file DataSheet8.ZIP › mouse liver of Placebo Group/Placebo3.jpg]

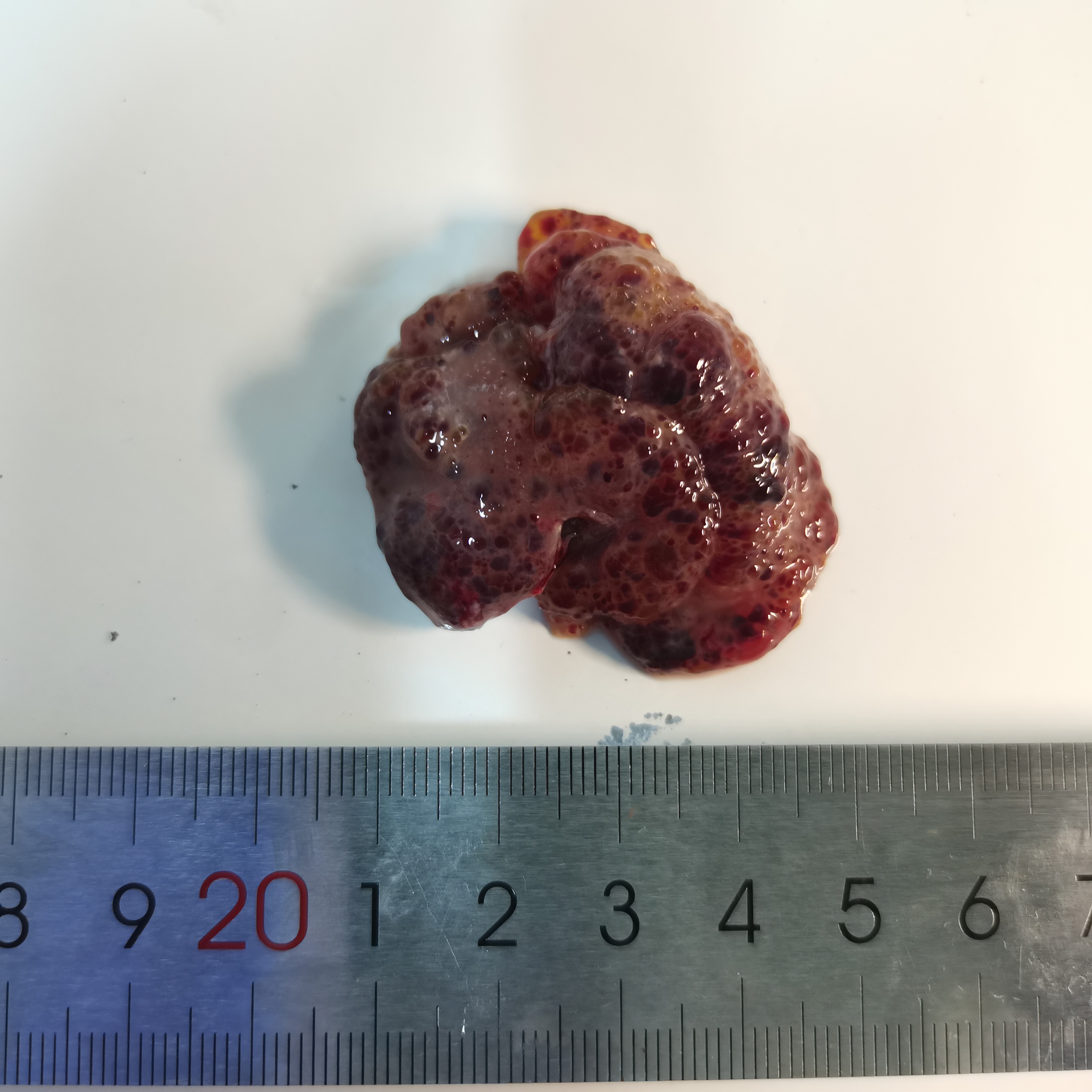

Supplement: Supplementary file 2 [file DataSheet8.ZIP › mouse liver of Placebo Group/Placebo4.jpg]

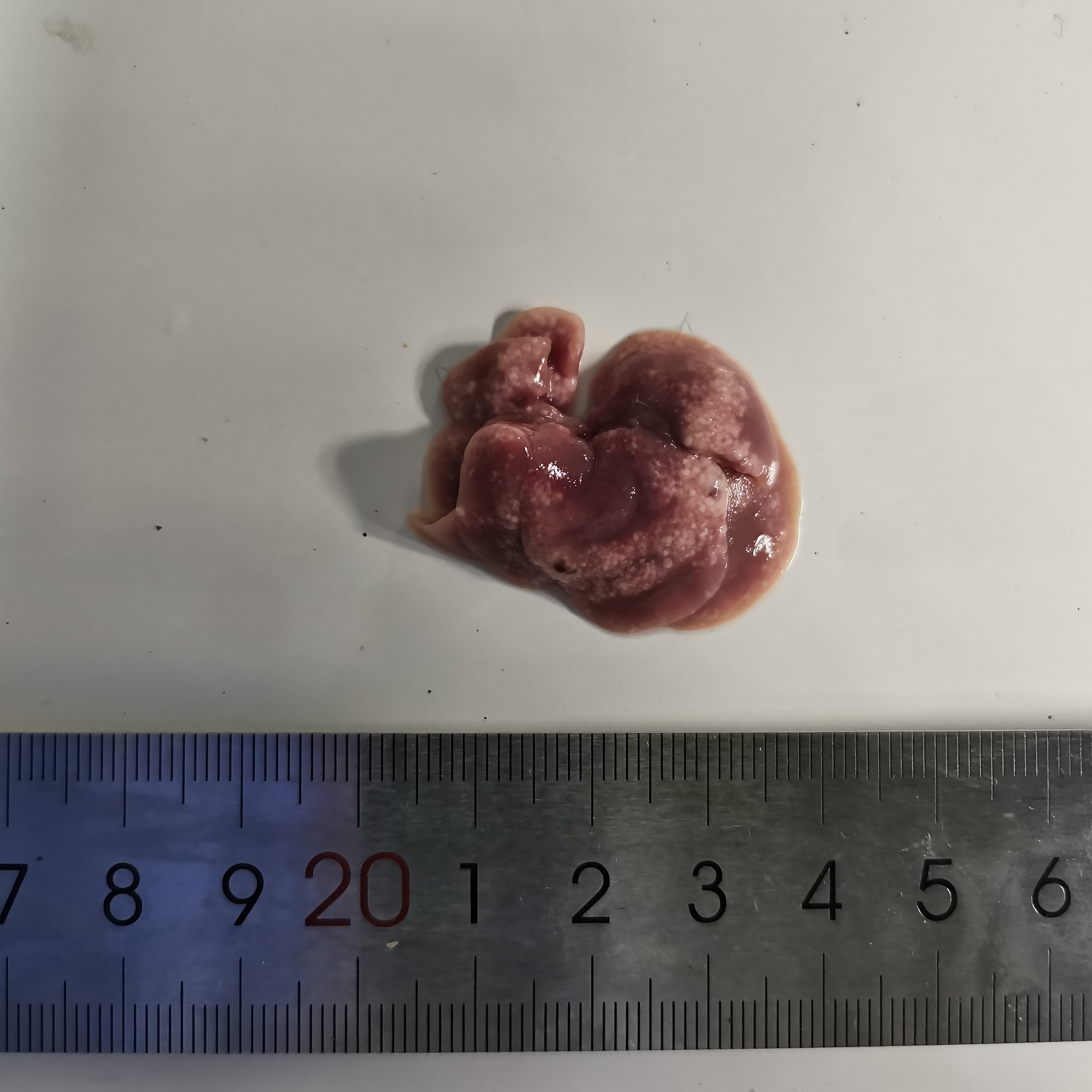

Supplement: Supplementary file 2 [file DataSheet8.ZIP › mouse liver of Placebo Group/Placebo5.jpg]

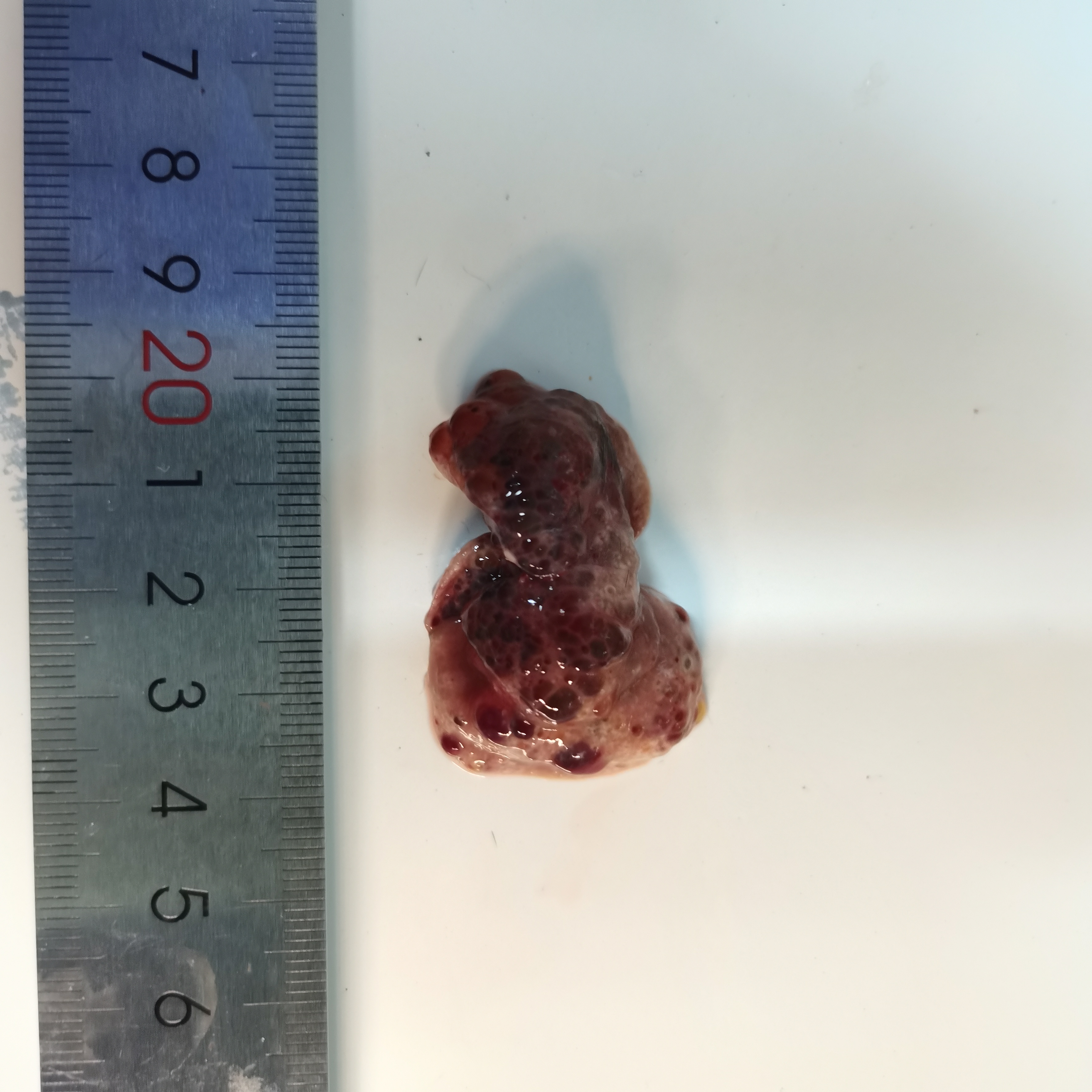

Supplement: Supplementary file 2 [file DataSheet8.ZIP › mouse liver of Placebo Group/Placebo6.jpg]

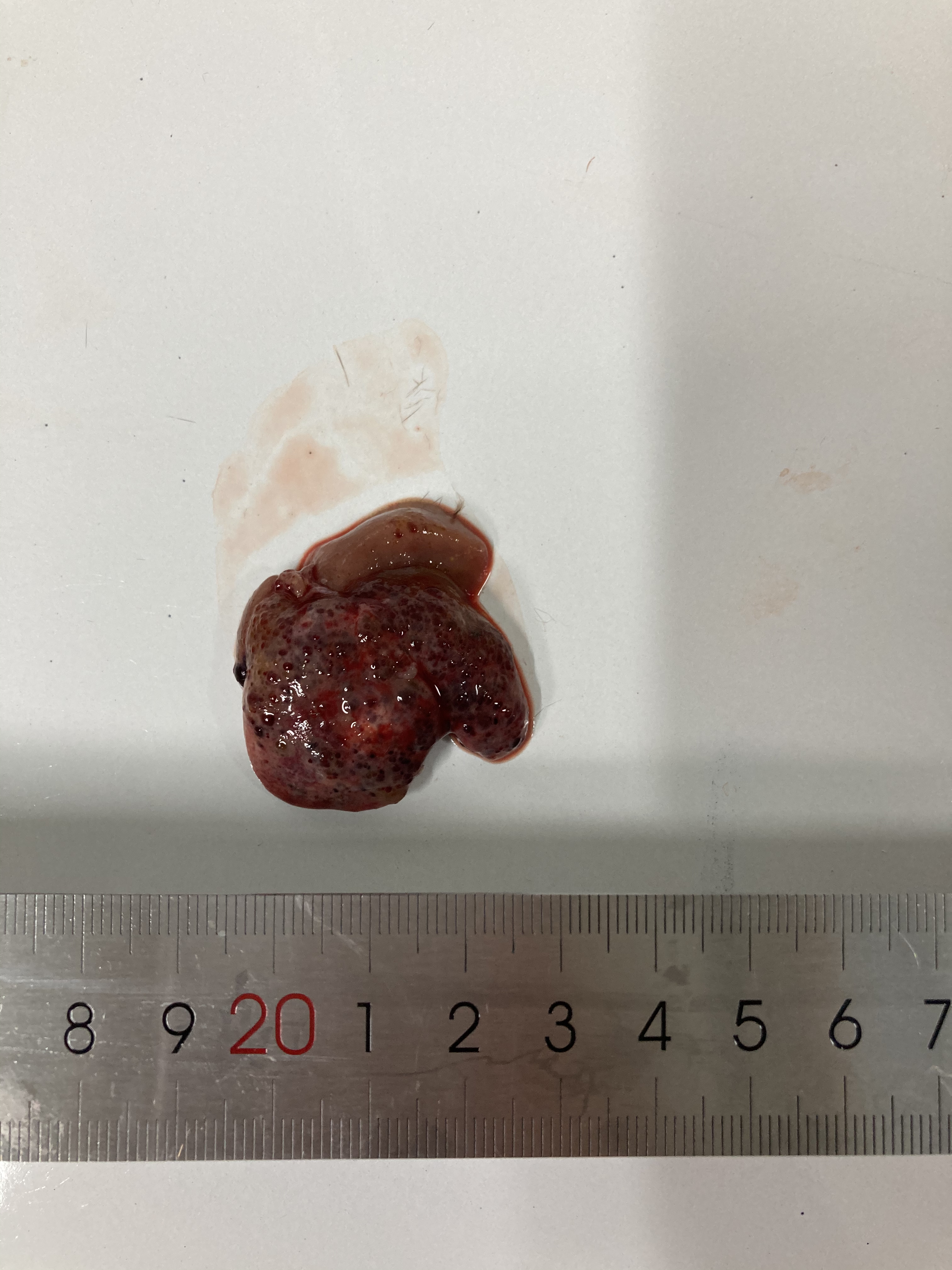

Supplement: Supplementary file 2 [file DataSheet8.ZIP › mouse liver of Placebo Group/Placebo7.jpg]

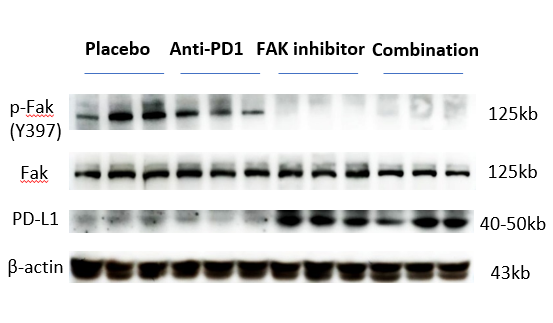

Supplement: Supplementary file 4 [file DataSheet1.ZIP › WB date/Fig6B.tif]

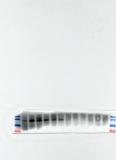

Supplement: Supplementary file 4 [file DataSheet1.ZIP › WB date/mouse p-FAK/mouse p-FAK (2).jpg]

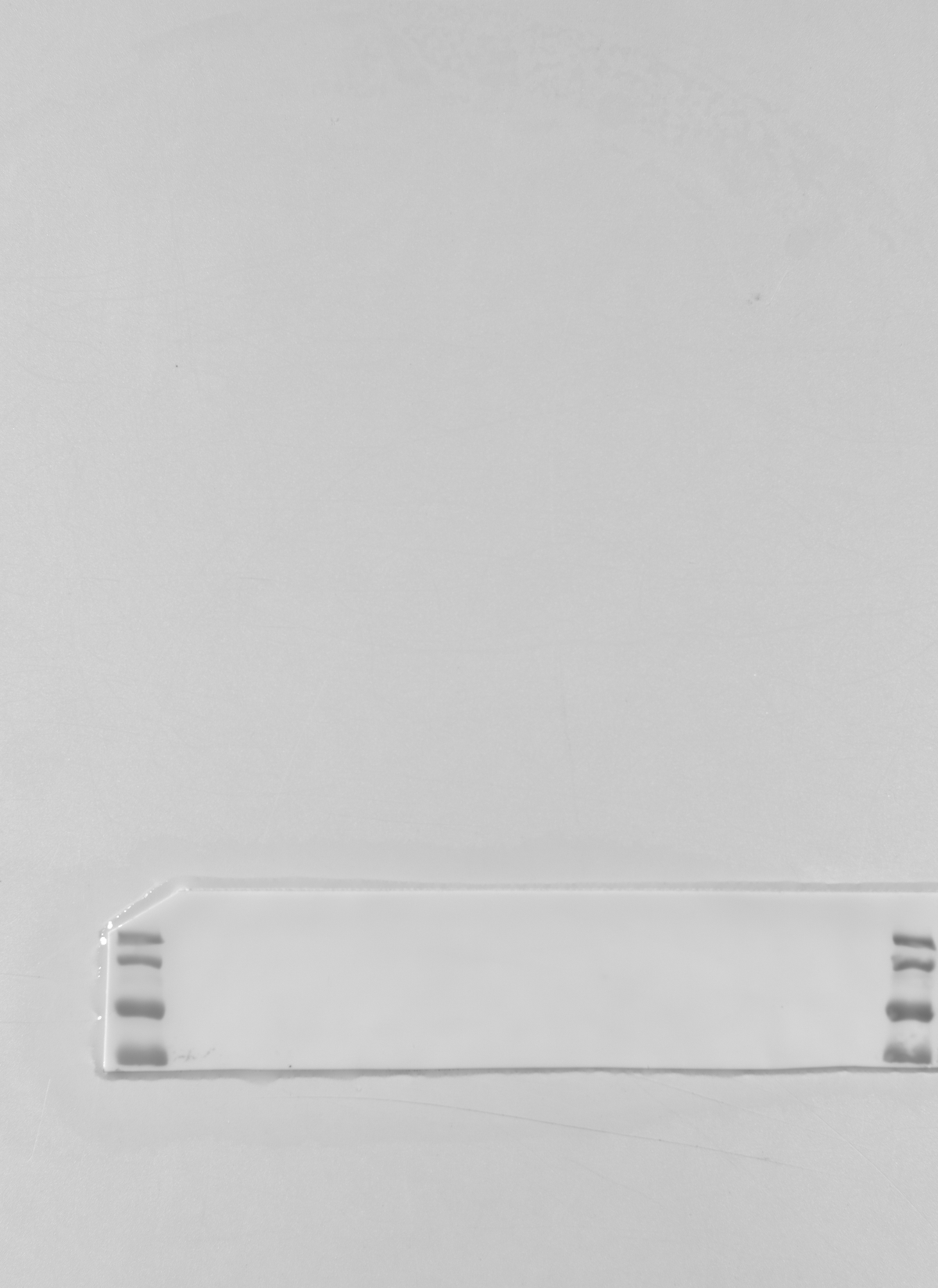

Supplement: Supplementary file 4 [file DataSheet1.ZIP › WB date/mouse p-FAK/mouse p-FAK (2).tif]

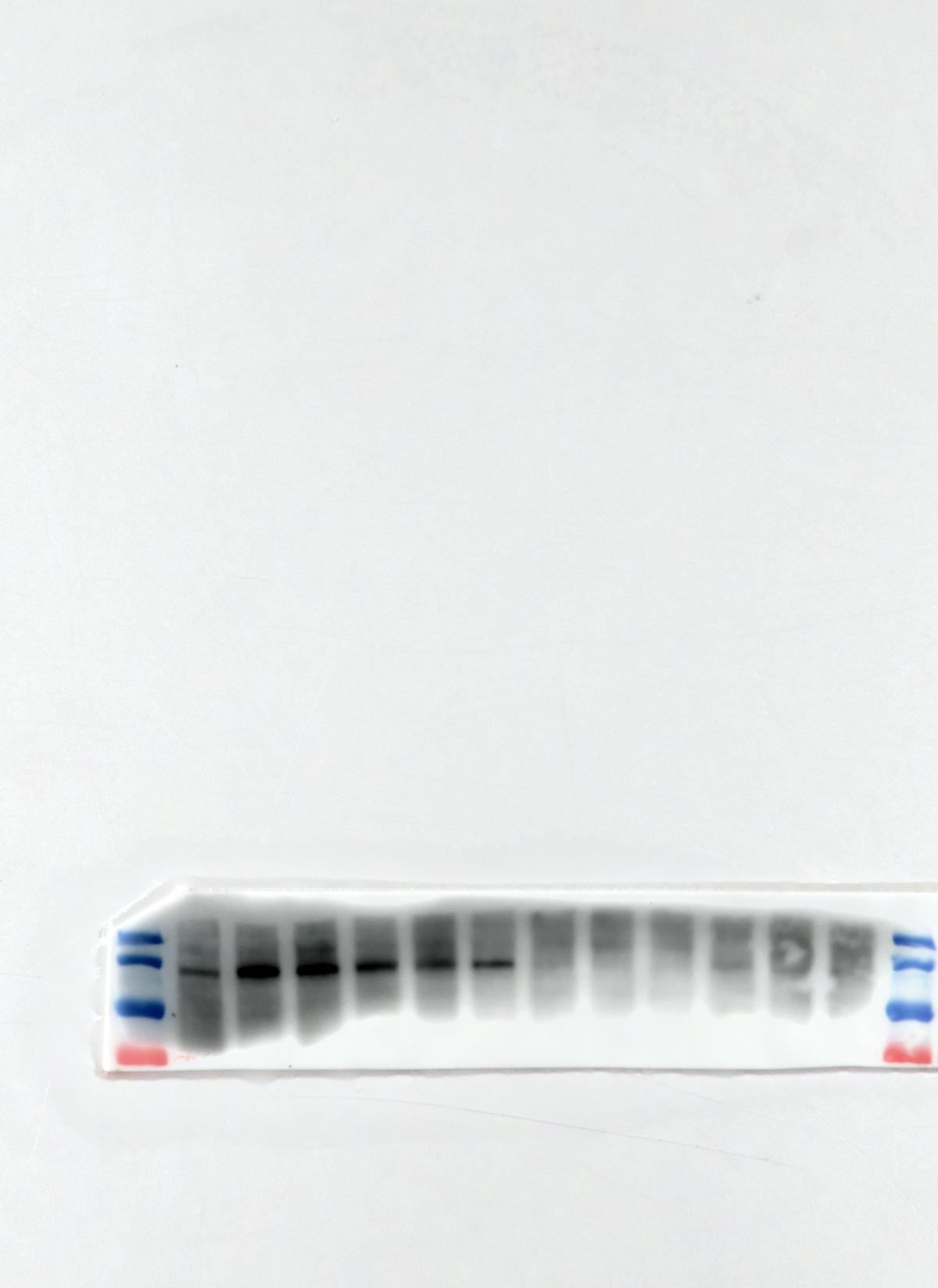

Supplement: Supplementary file 4 [file DataSheet1.ZIP › WB date/mouse p-FAK/mouse p-FAK (3).jpg]

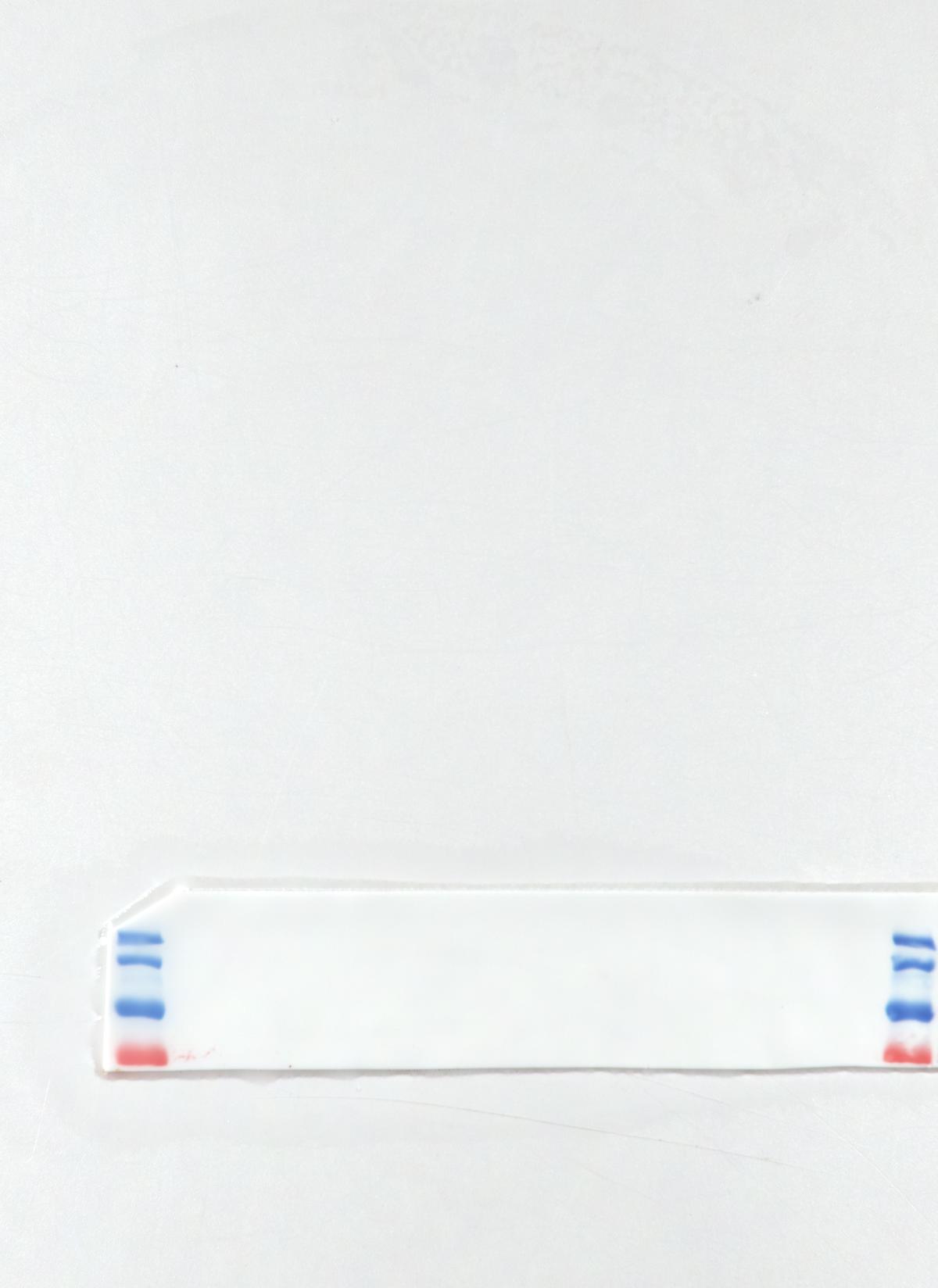

Supplement: Supplementary file 4 [file DataSheet1.ZIP › WB date/mouse p-FAK/mouse p-FAK.jpg]

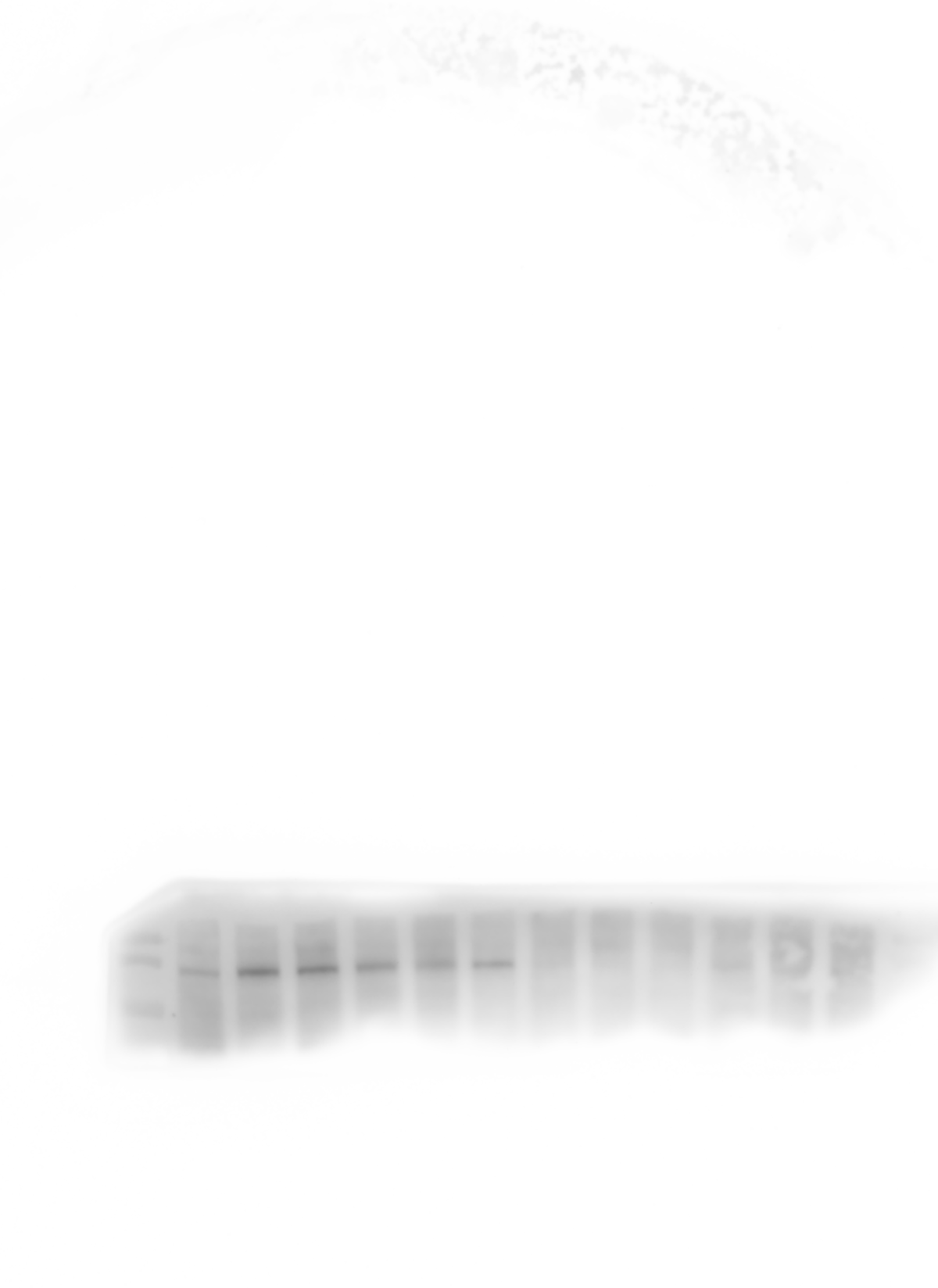

Supplement: Supplementary file 4 [file DataSheet1.ZIP › WB date/mouse p-FAK/mouse p-FAK.tif]

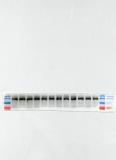

Supplement: Supplementary file 4 [file DataSheet1.ZIP › WB date/mouse FAK/mouse FAK (2).jpg]

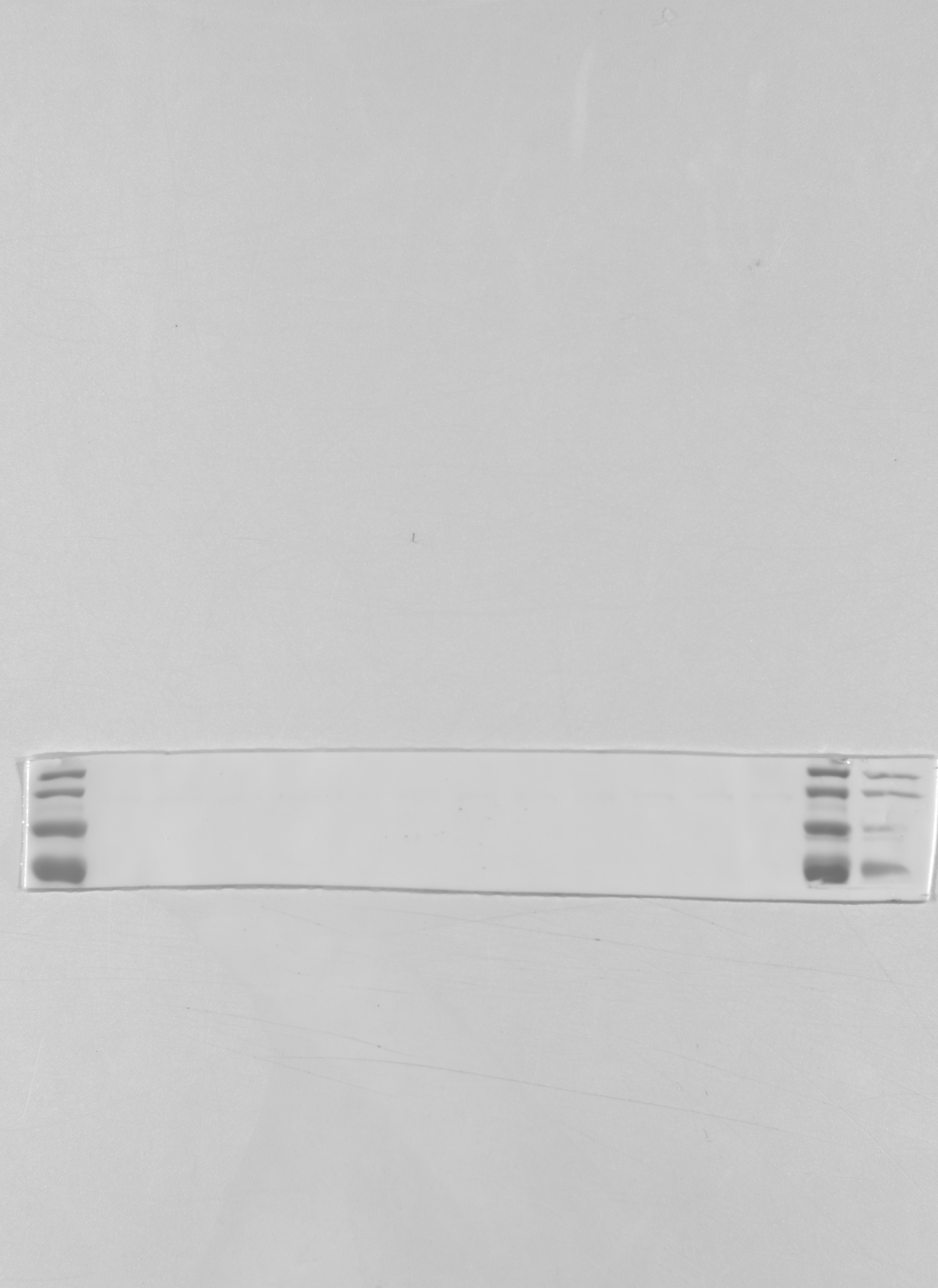

Supplement: Supplementary file 4 [file DataSheet1.ZIP › WB date/mouse FAK/mouse FAK (2).tif]

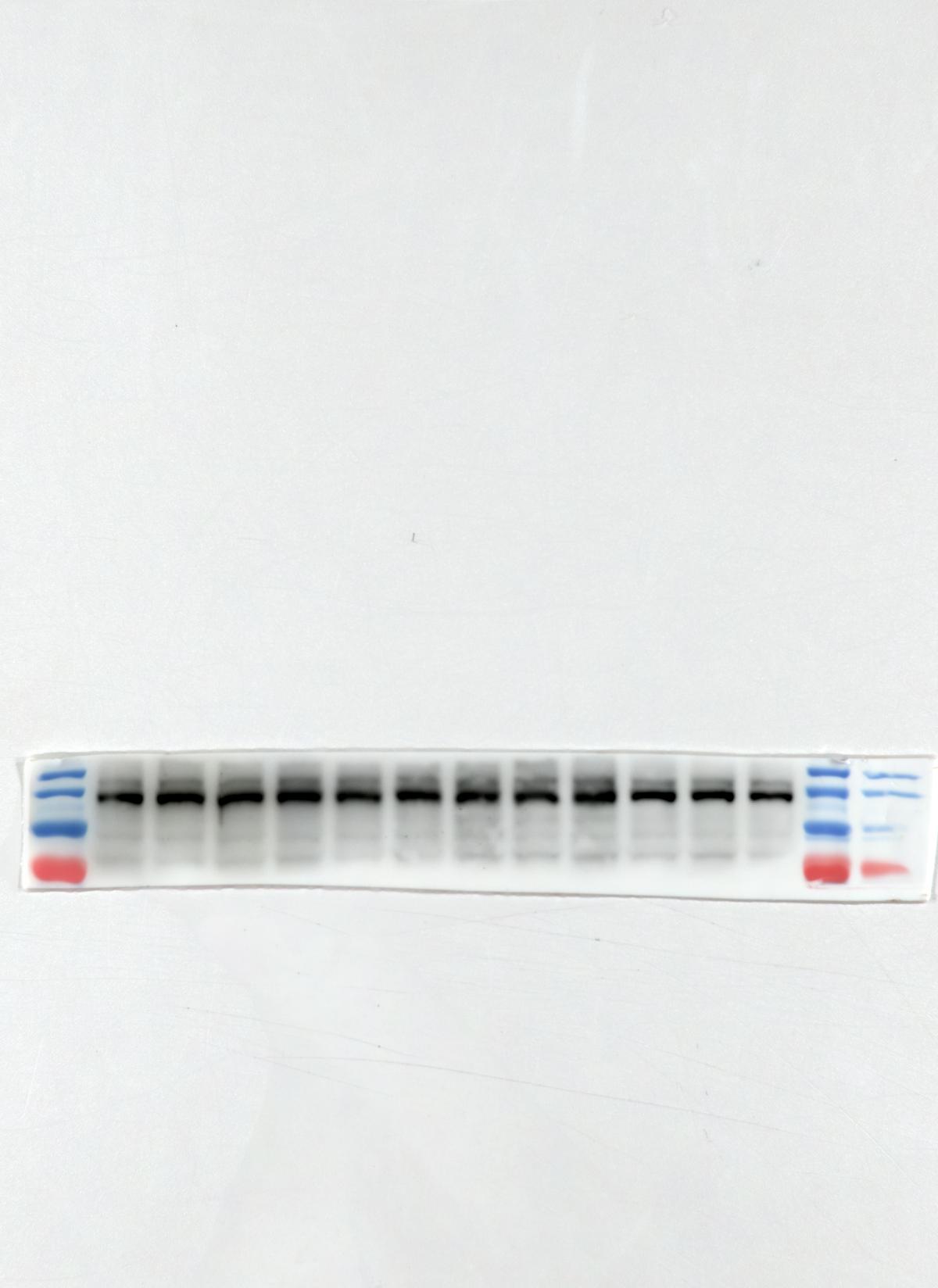

Supplement: Supplementary file 4 [file DataSheet1.ZIP › WB date/mouse FAK/mouse FAK (3).jpg]

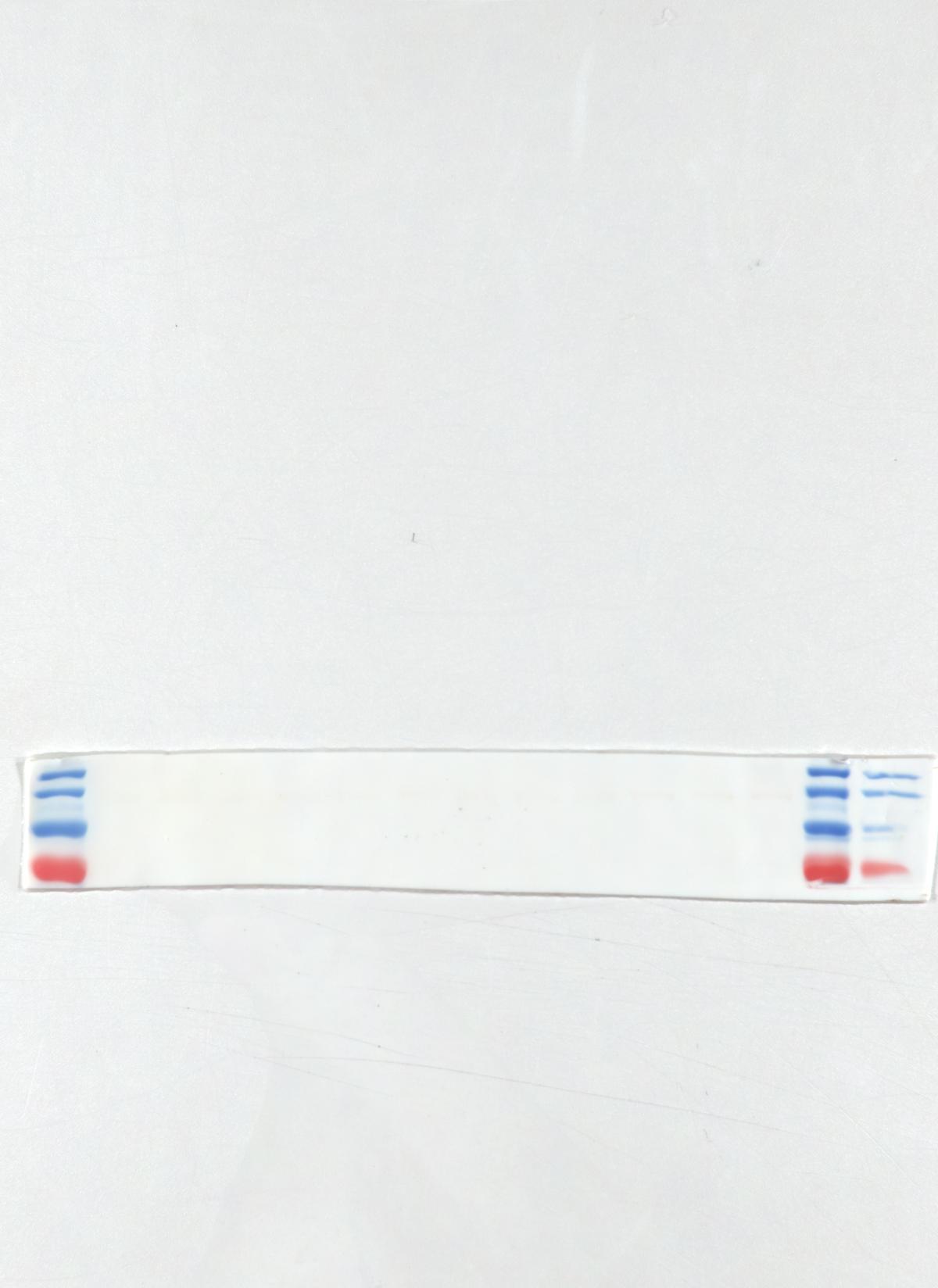

Supplement: Supplementary file 4 [file DataSheet1.ZIP › WB date/mouse FAK/mouse FAK.jpg]

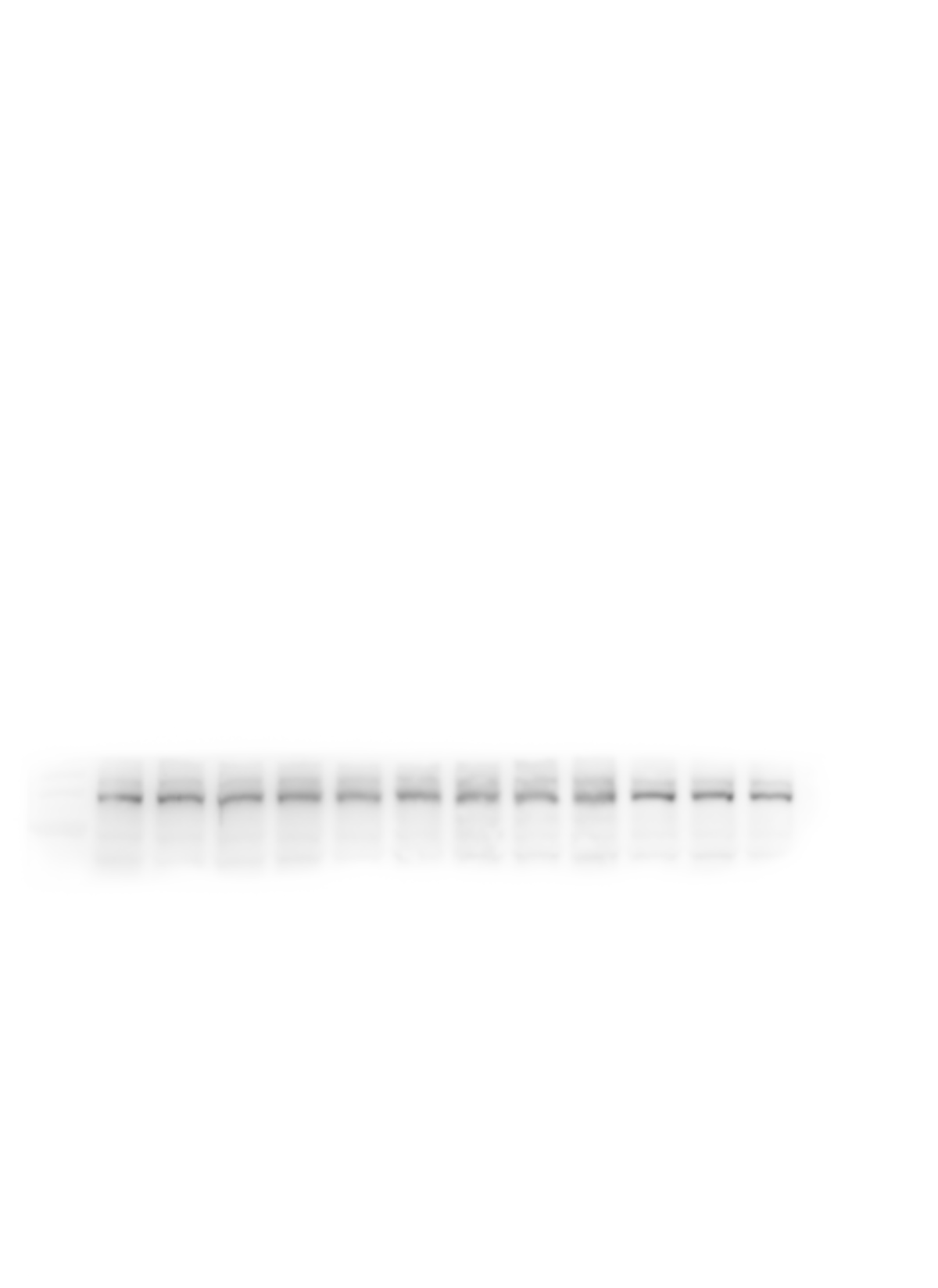

Supplement: Supplementary file 4 [file DataSheet1.ZIP › WB date/mouse FAK/mouse FAK.tif]

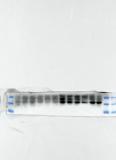

Supplement: Supplementary file 4 [file DataSheet1.ZIP › WB date/mouse PD-L1/mouse PD-L1 (2).jpg]

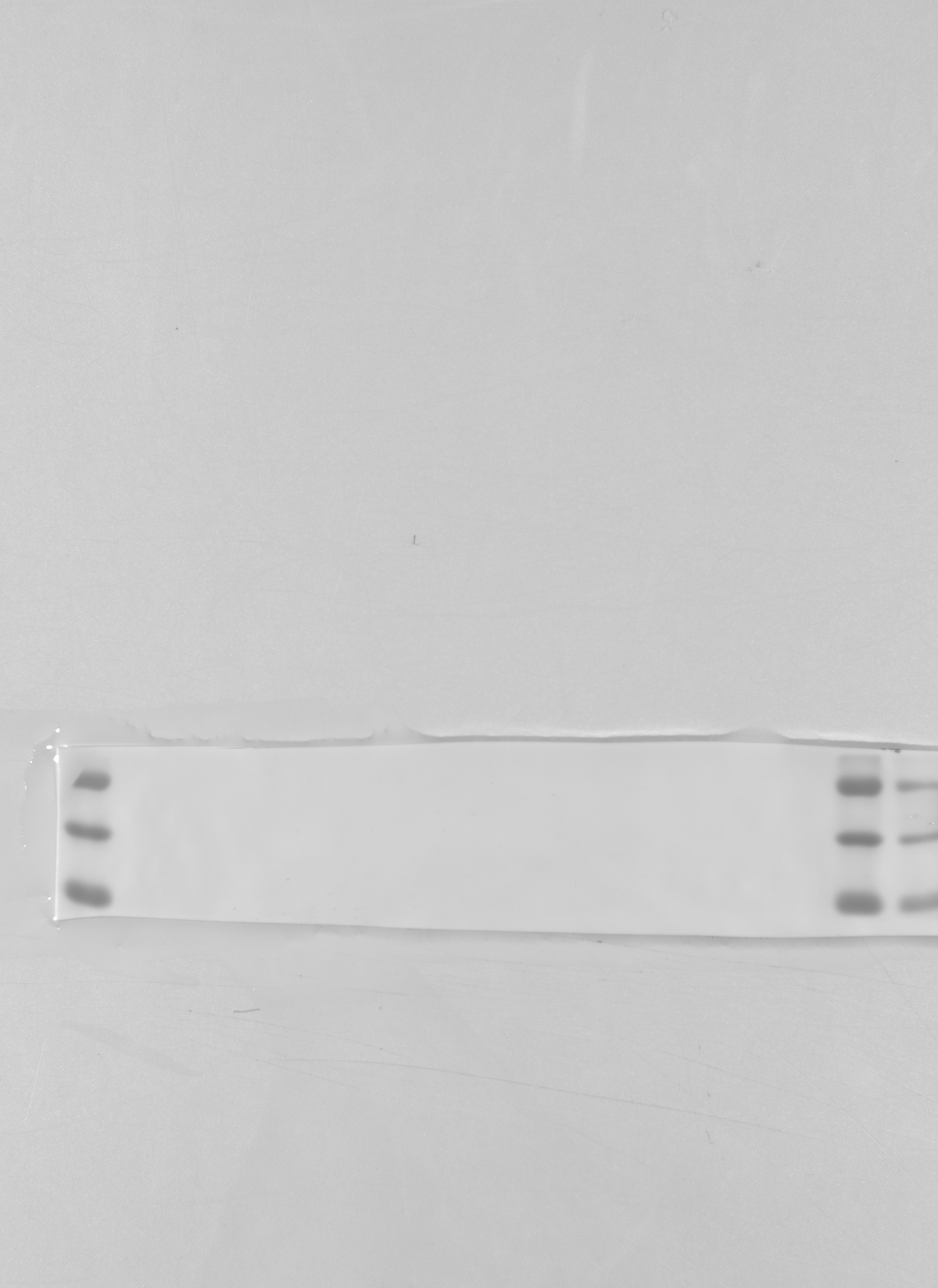

Supplement: Supplementary file 4 [file DataSheet1.ZIP › WB date/mouse PD-L1/mouse PD-L1 (2).tif]

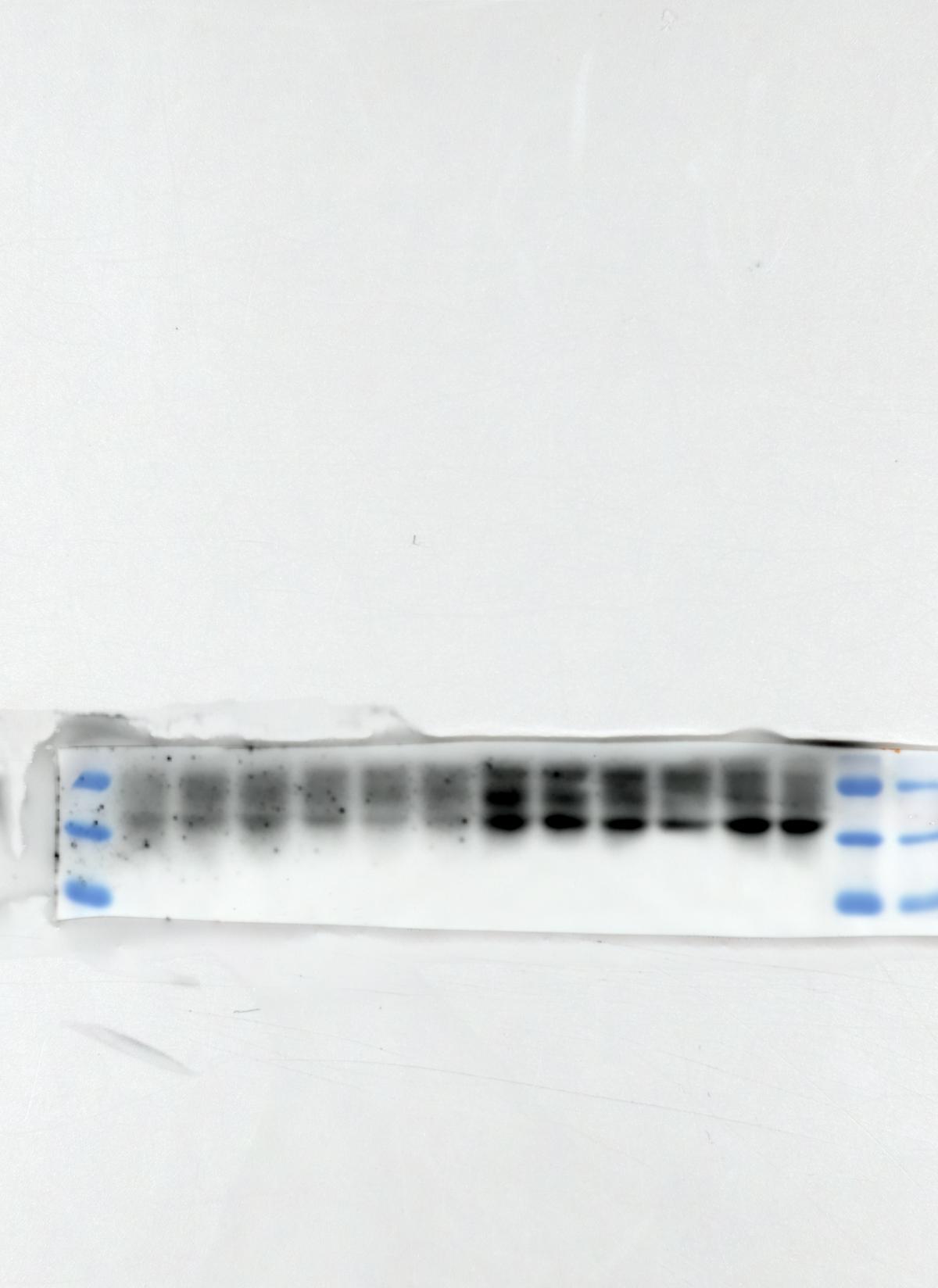

Supplement: Supplementary file 4 [file DataSheet1.ZIP › WB date/mouse PD-L1/mouse PD-L1 (3).jpg]

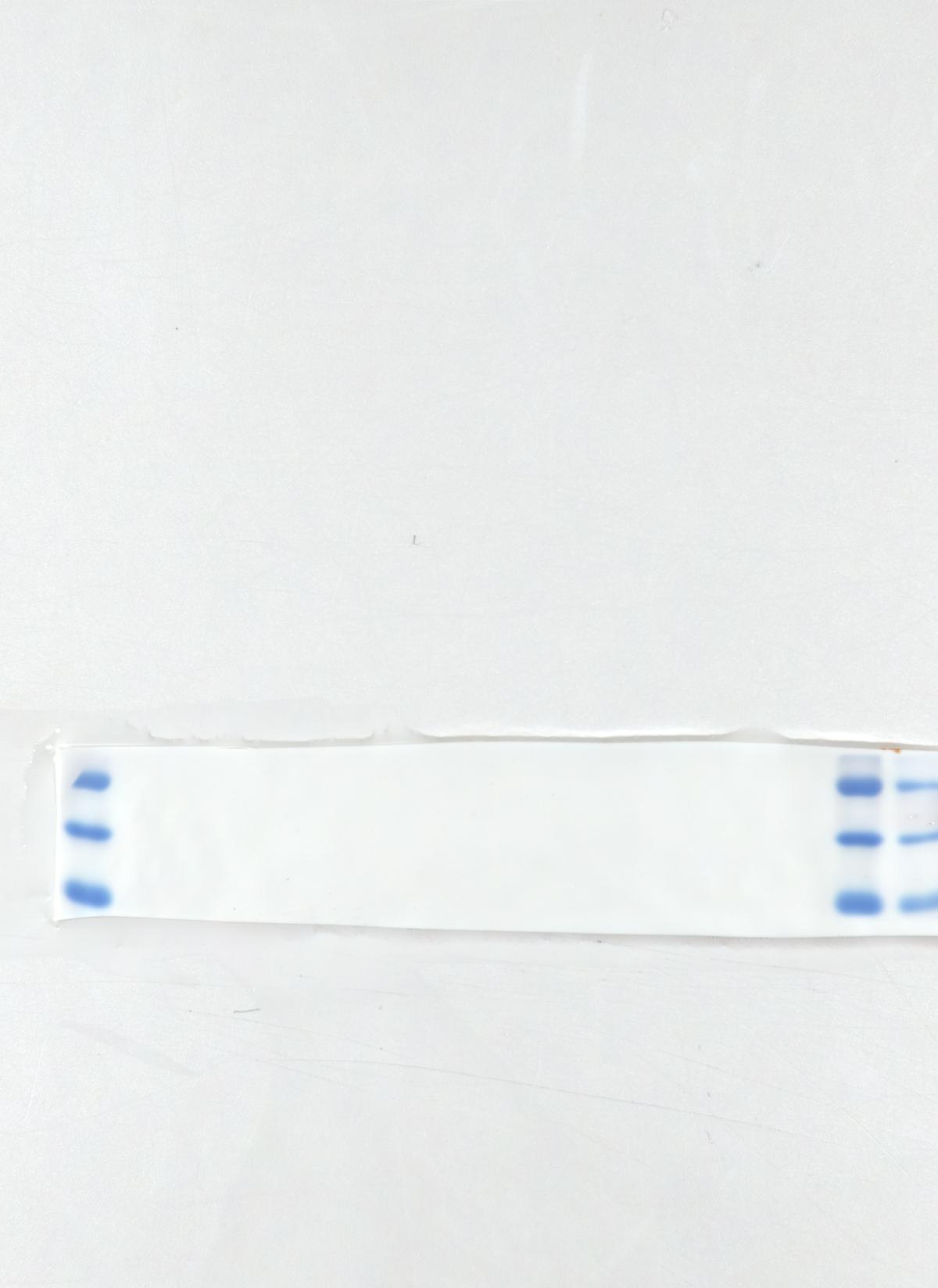

Supplement: Supplementary file 4 [file DataSheet1.ZIP › WB date/mouse PD-L1/mouse PD-L1.jpg]

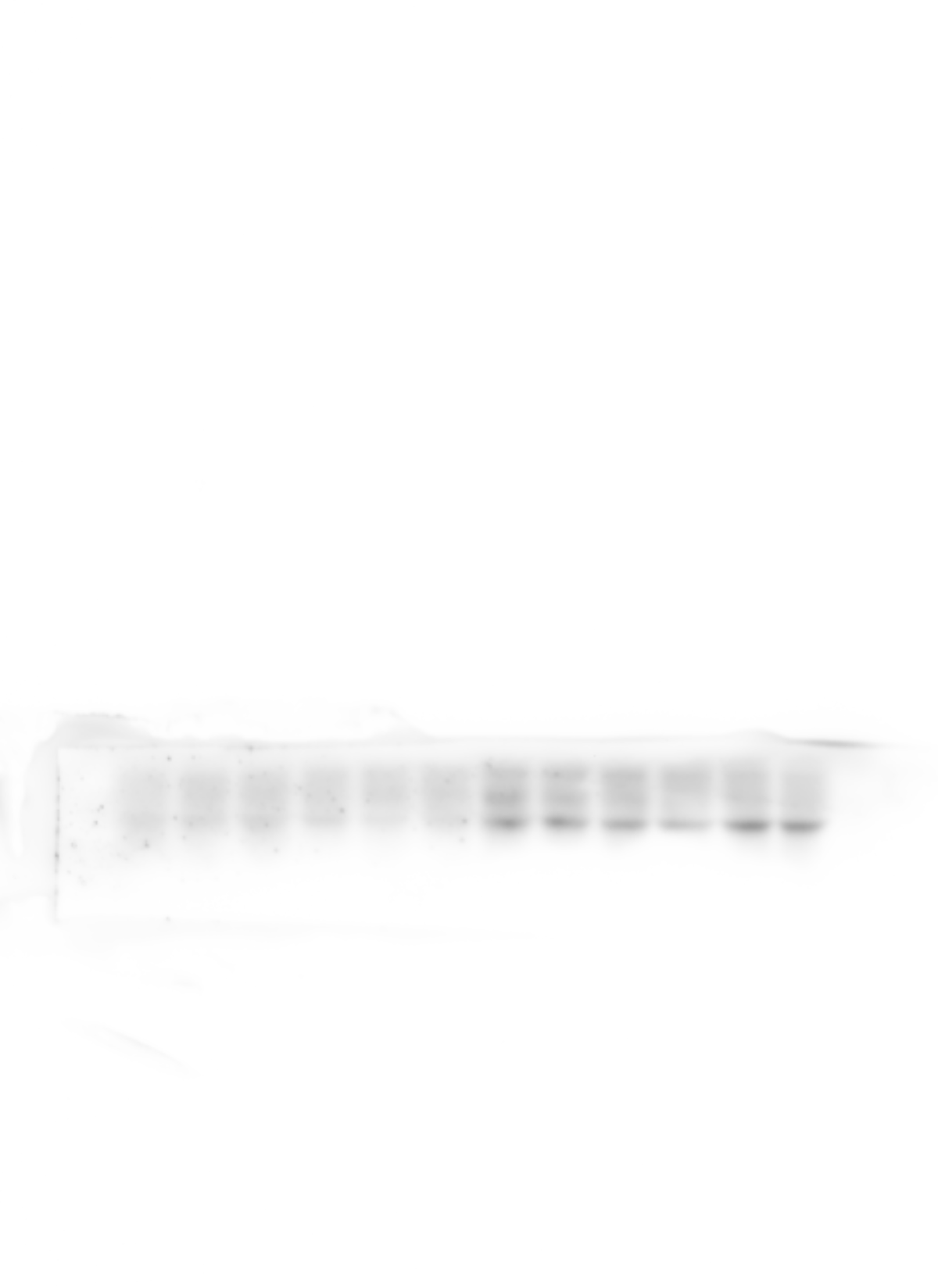

Supplement: Supplementary file 4 [file DataSheet1.ZIP › WB date/mouse PD-L1/mouse PD-L1.tif]

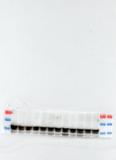

Supplement: Supplementary file 4 [file DataSheet1.ZIP › WB date/mouse actin/mouse actin (2).jpg]

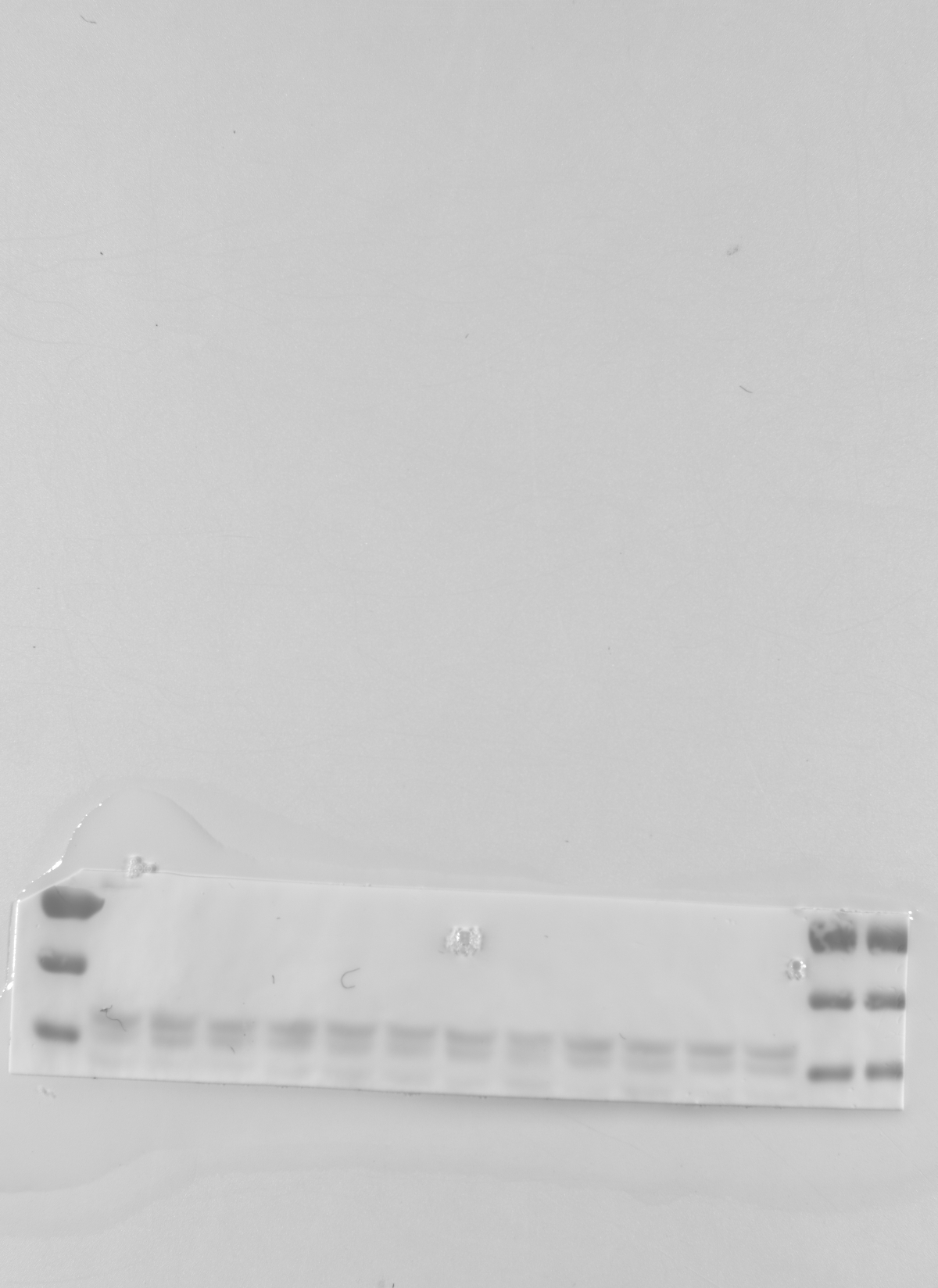

Supplement: Supplementary file 4 [file DataSheet1.ZIP › WB date/mouse actin/mouse actin (2).tif]

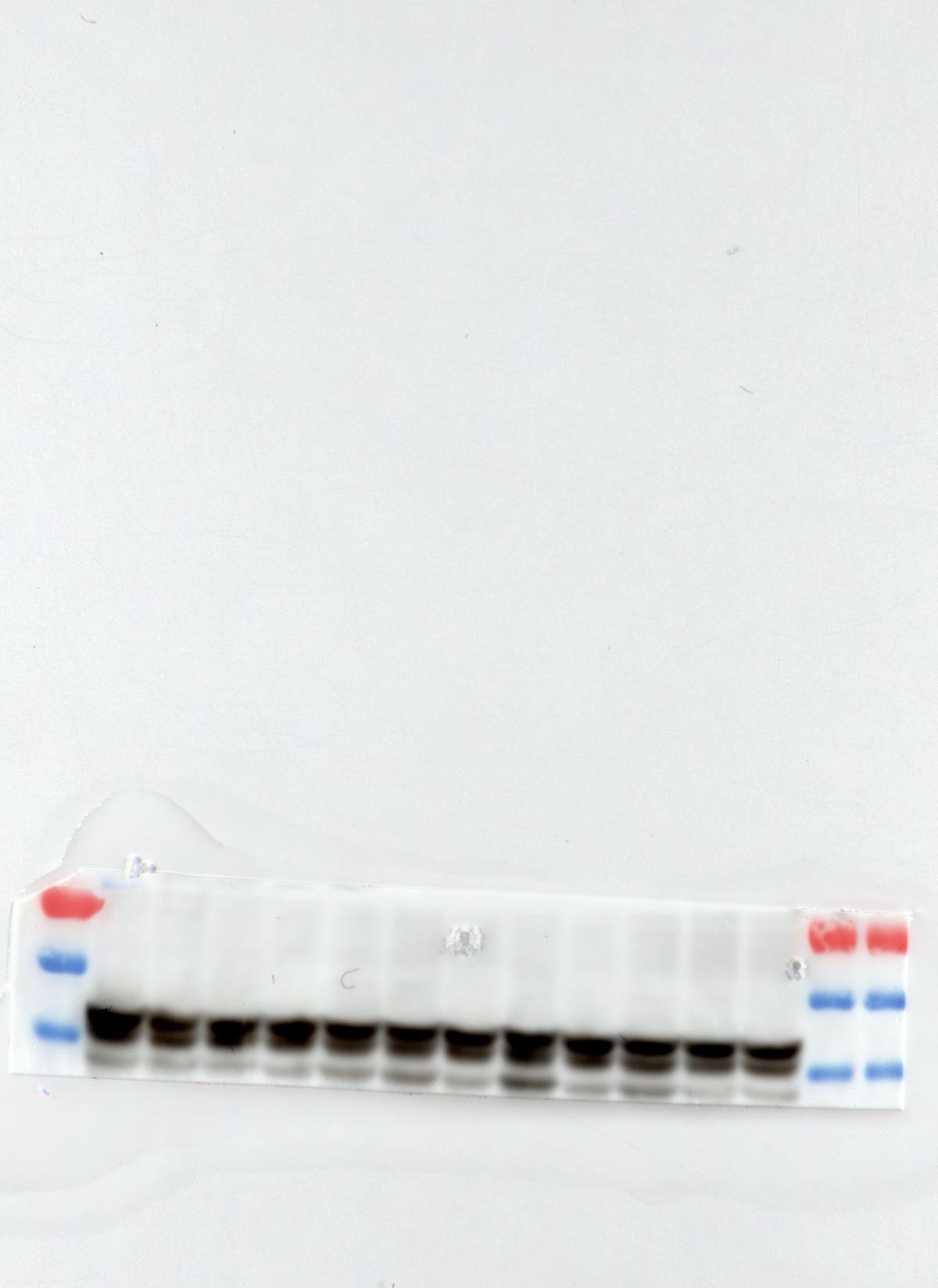

Supplement: Supplementary file 4 [file DataSheet1.ZIP › WB date/mouse actin/mouse actin (3).jpg]

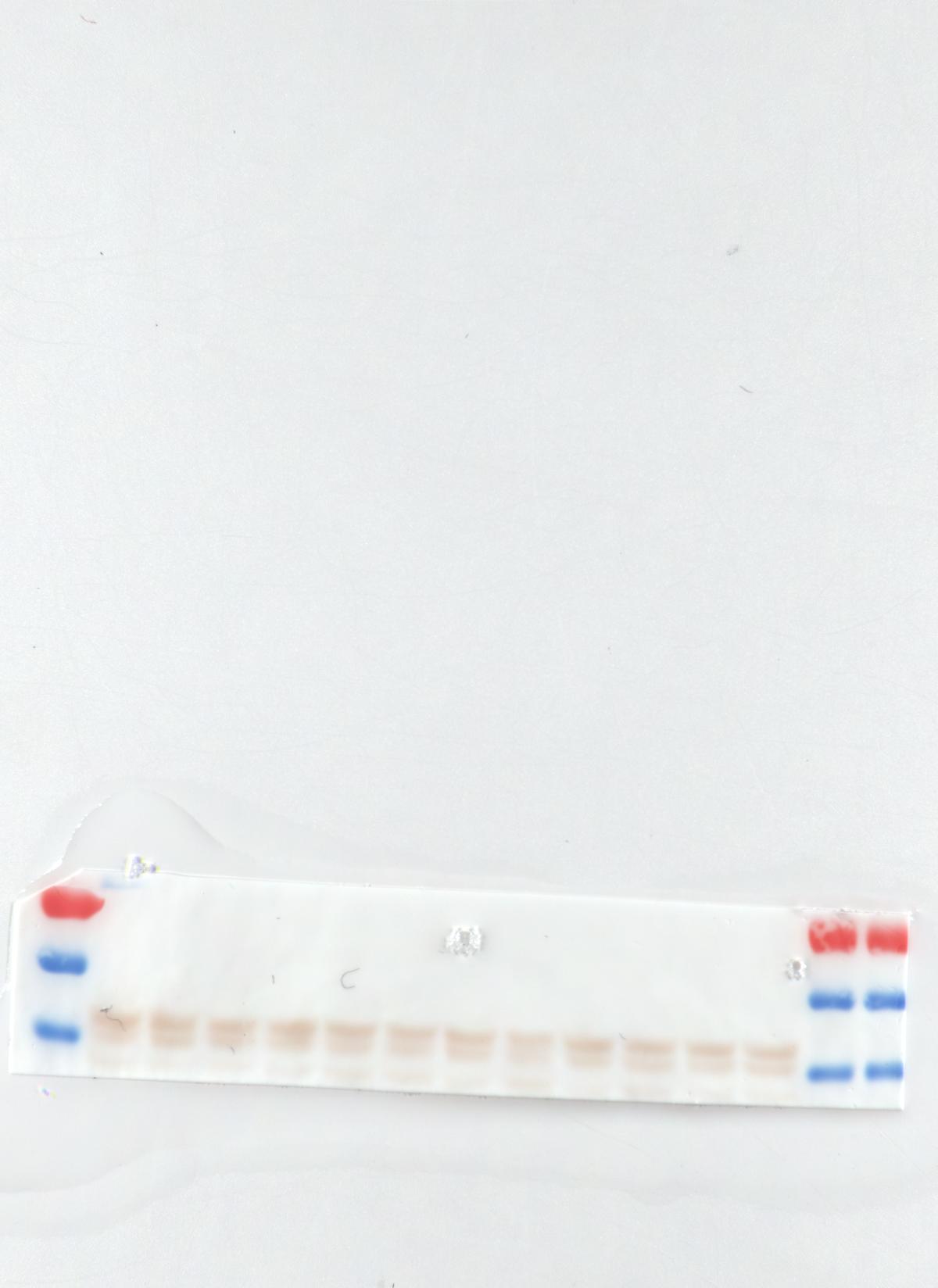

Supplement: Supplementary file 4 [file DataSheet1.ZIP › WB date/mouse actin/mouse actin.jpg]

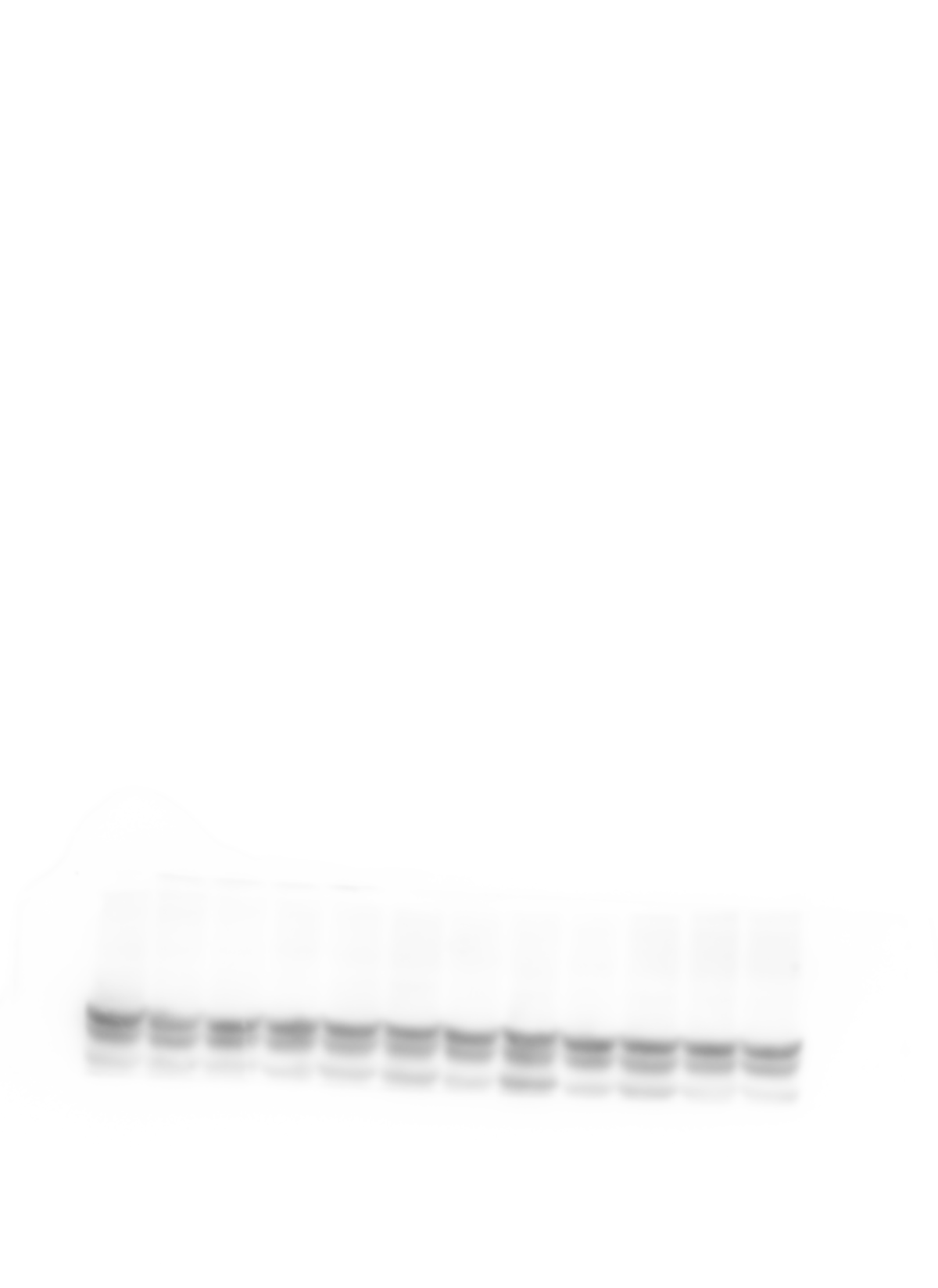

Supplement: Supplementary file 4 [file DataSheet1.ZIP › WB date/mouse actin/mouse actin.tif]

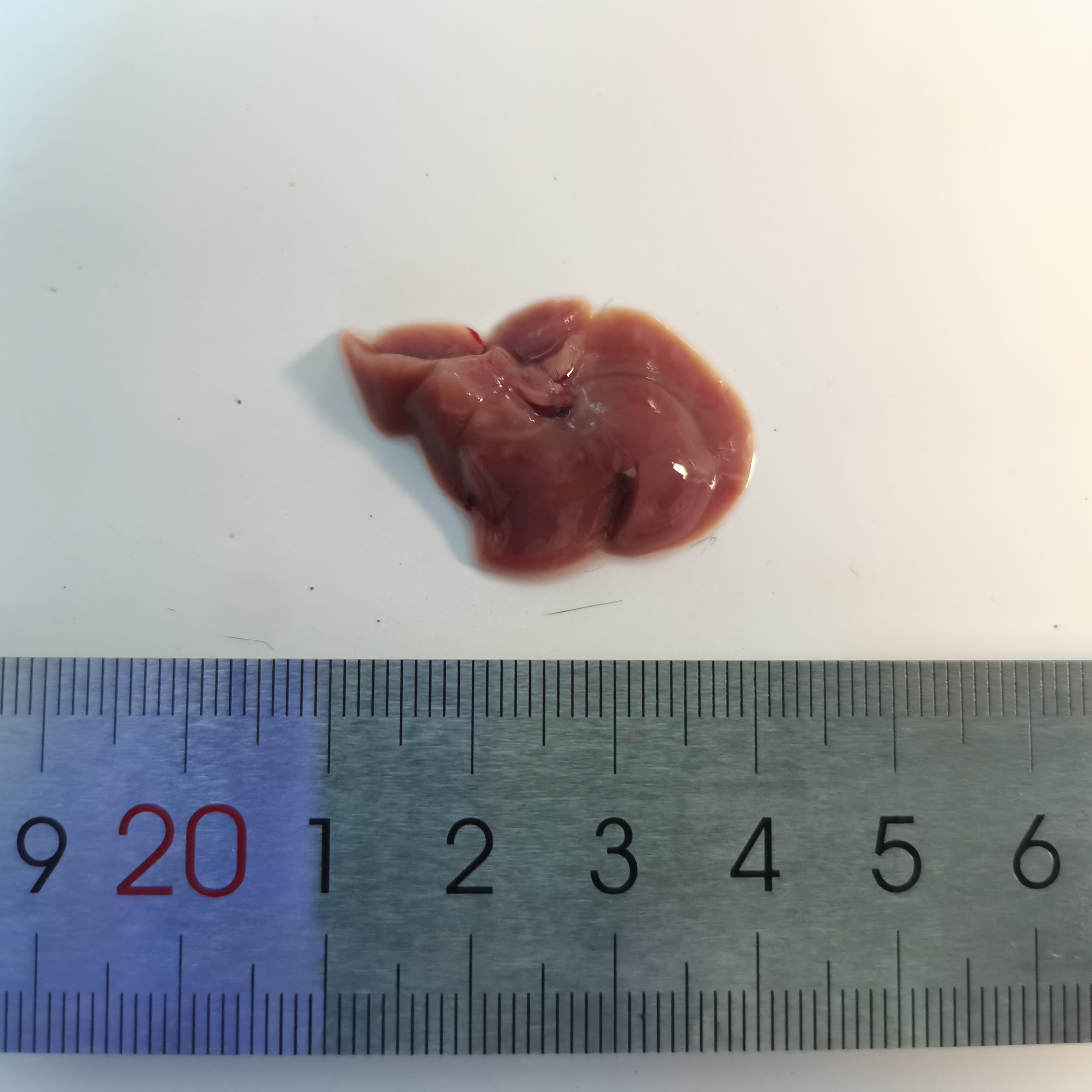

Supplement: Supplementary file 5 [file DataSheet6.ZIP › mouse liver of Combination Group/Combination 2.jpg]

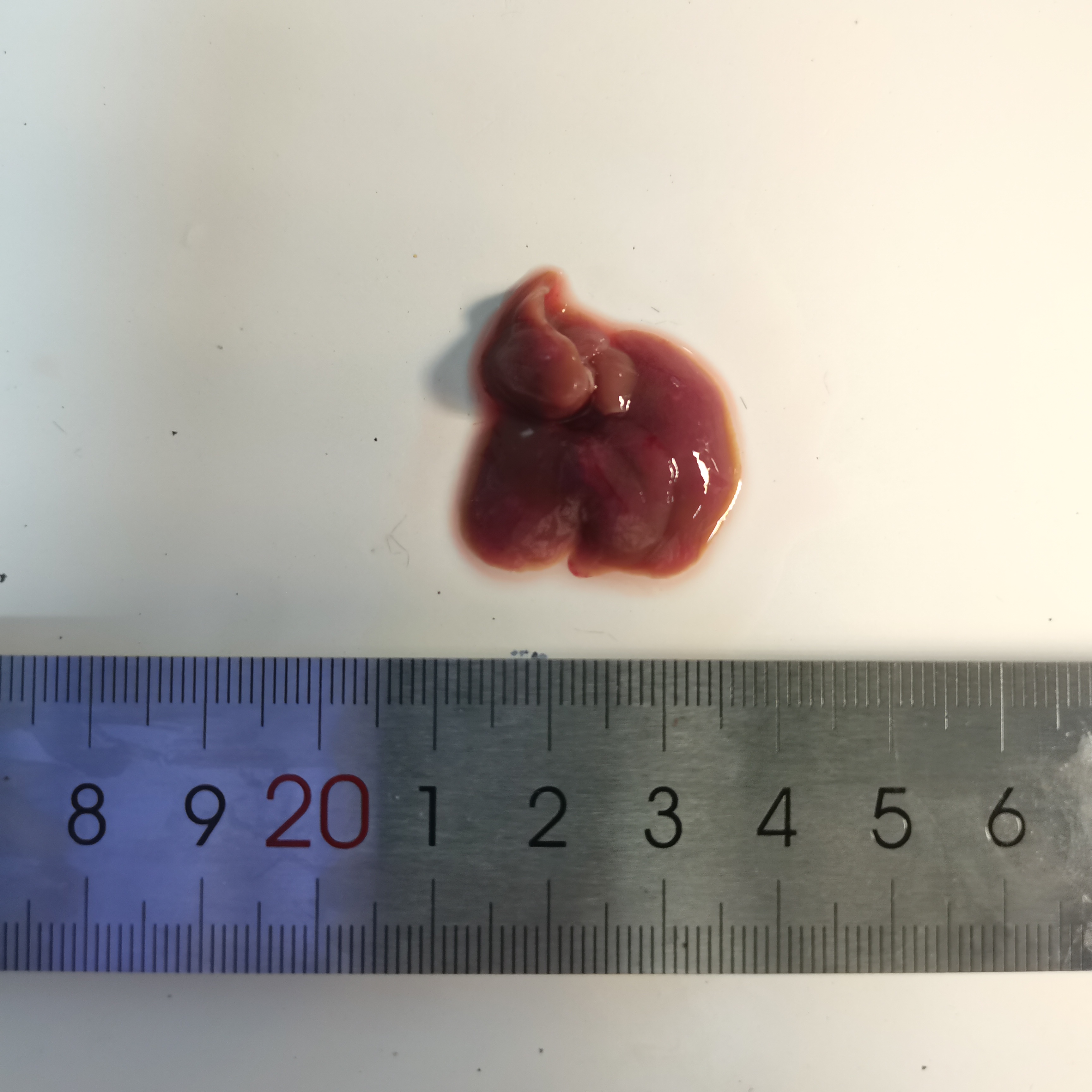

Supplement: Supplementary file 5 [file DataSheet6.ZIP › mouse liver of Combination Group/Combination 8.jpg]

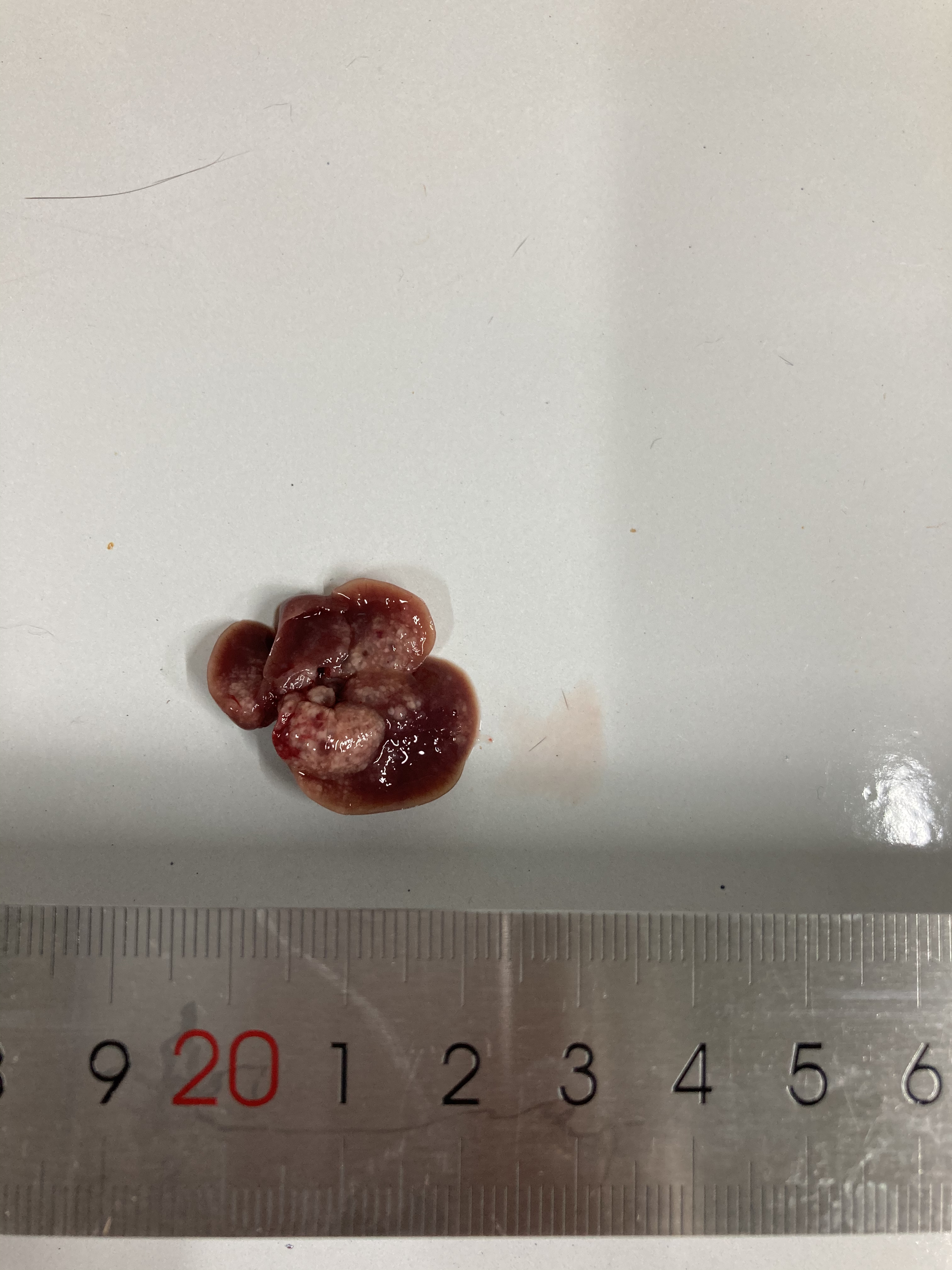

Supplement: Supplementary file 5 [file DataSheet6.ZIP › mouse liver of Combination Group/Combination.jpg]

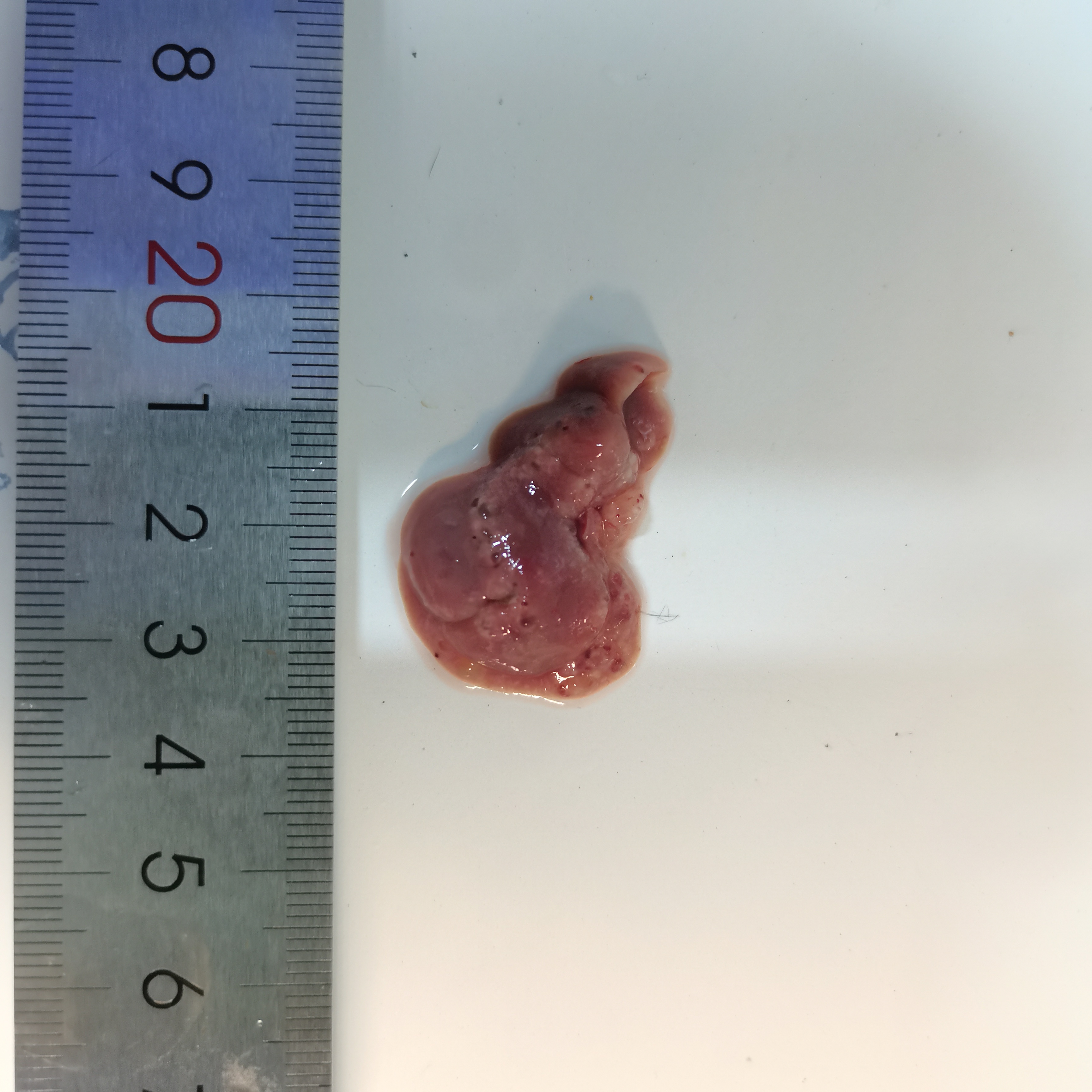

Supplement: Supplementary file 5 [file DataSheet6.ZIP › mouse liver of Combination Group/Combination1.jpg]

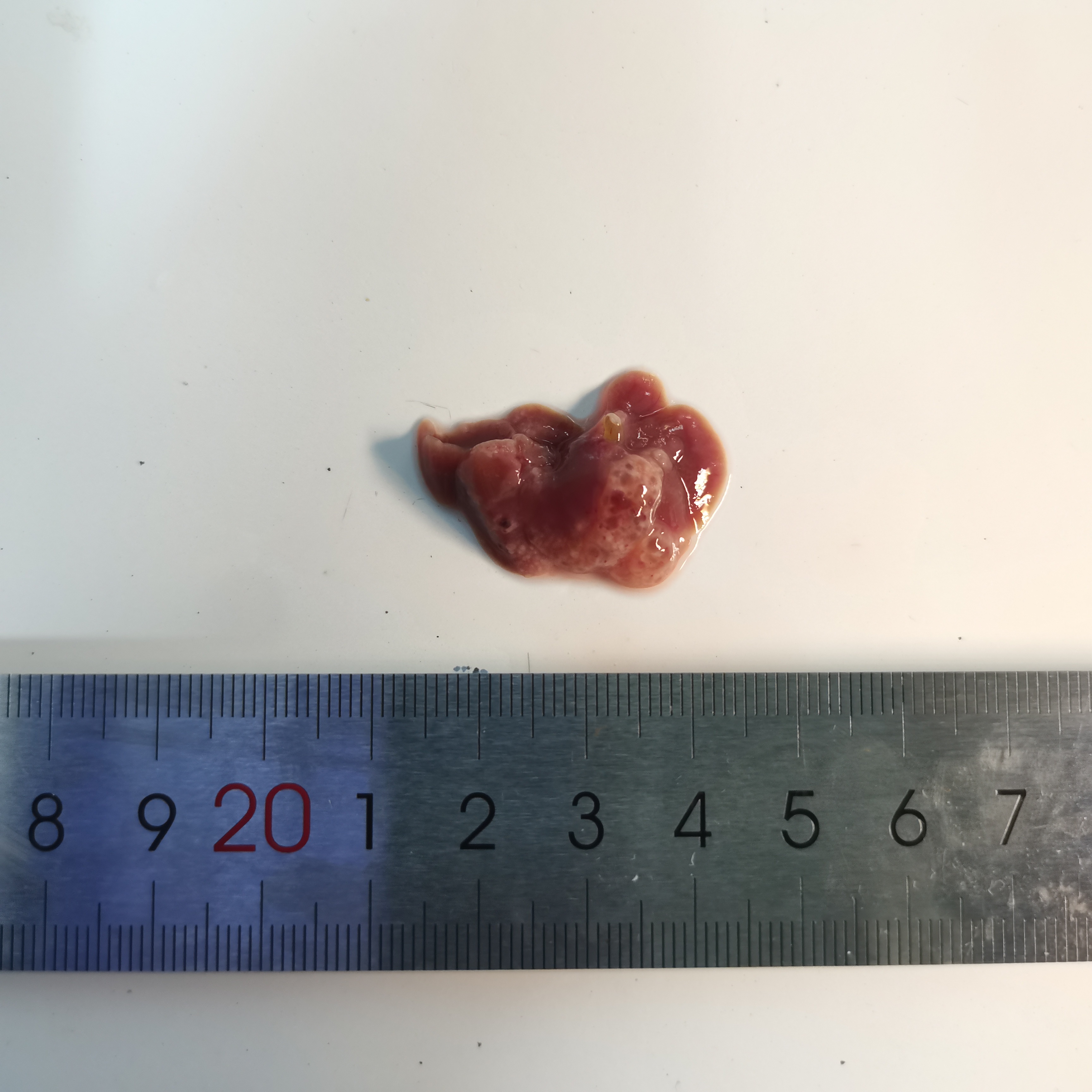

Supplement: Supplementary file 5 [file DataSheet6.ZIP › mouse liver of Combination Group/Combination3.jpg]

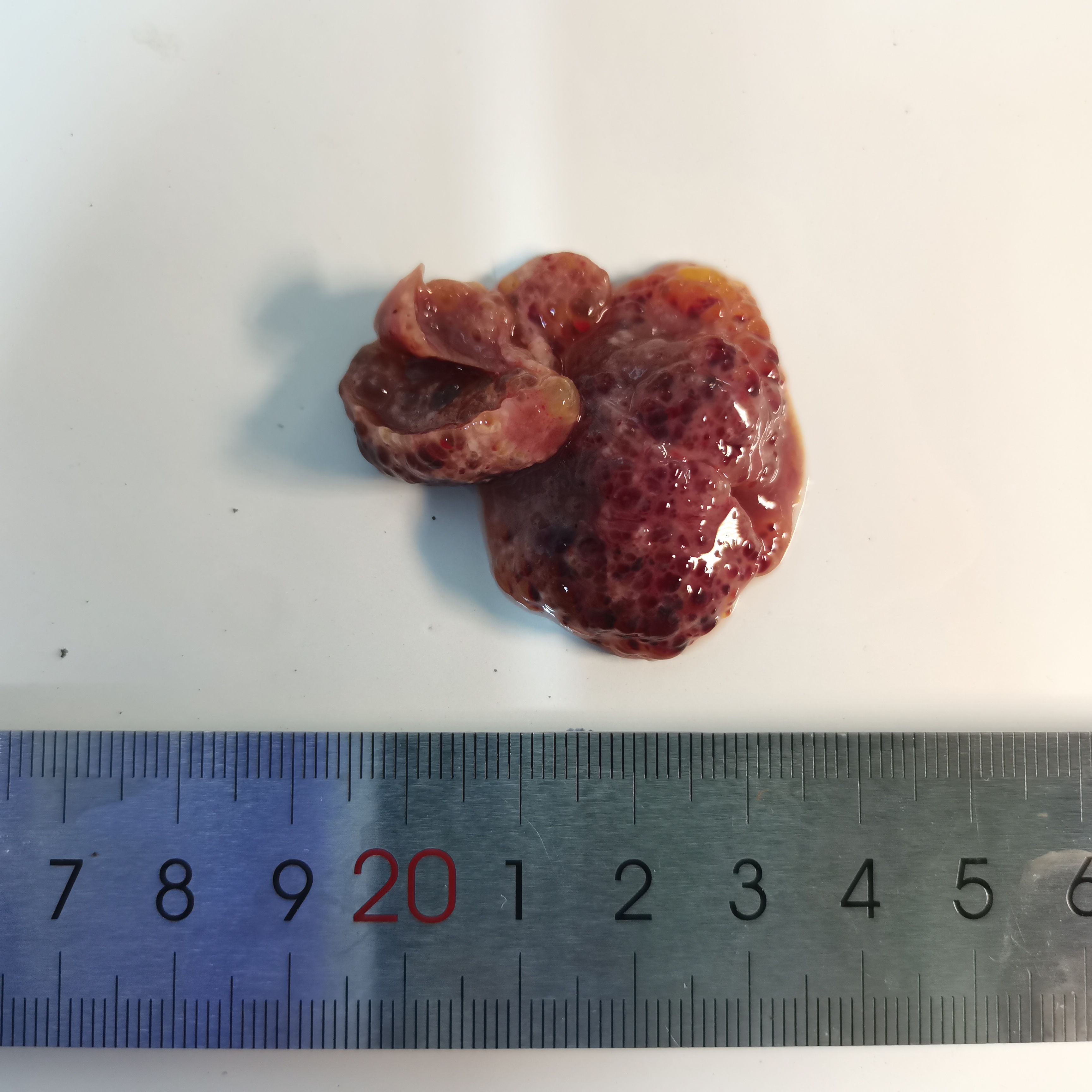

Supplement: Supplementary file 5 [file DataSheet6.ZIP › mouse liver of Combination Group/Combination4.jpg]

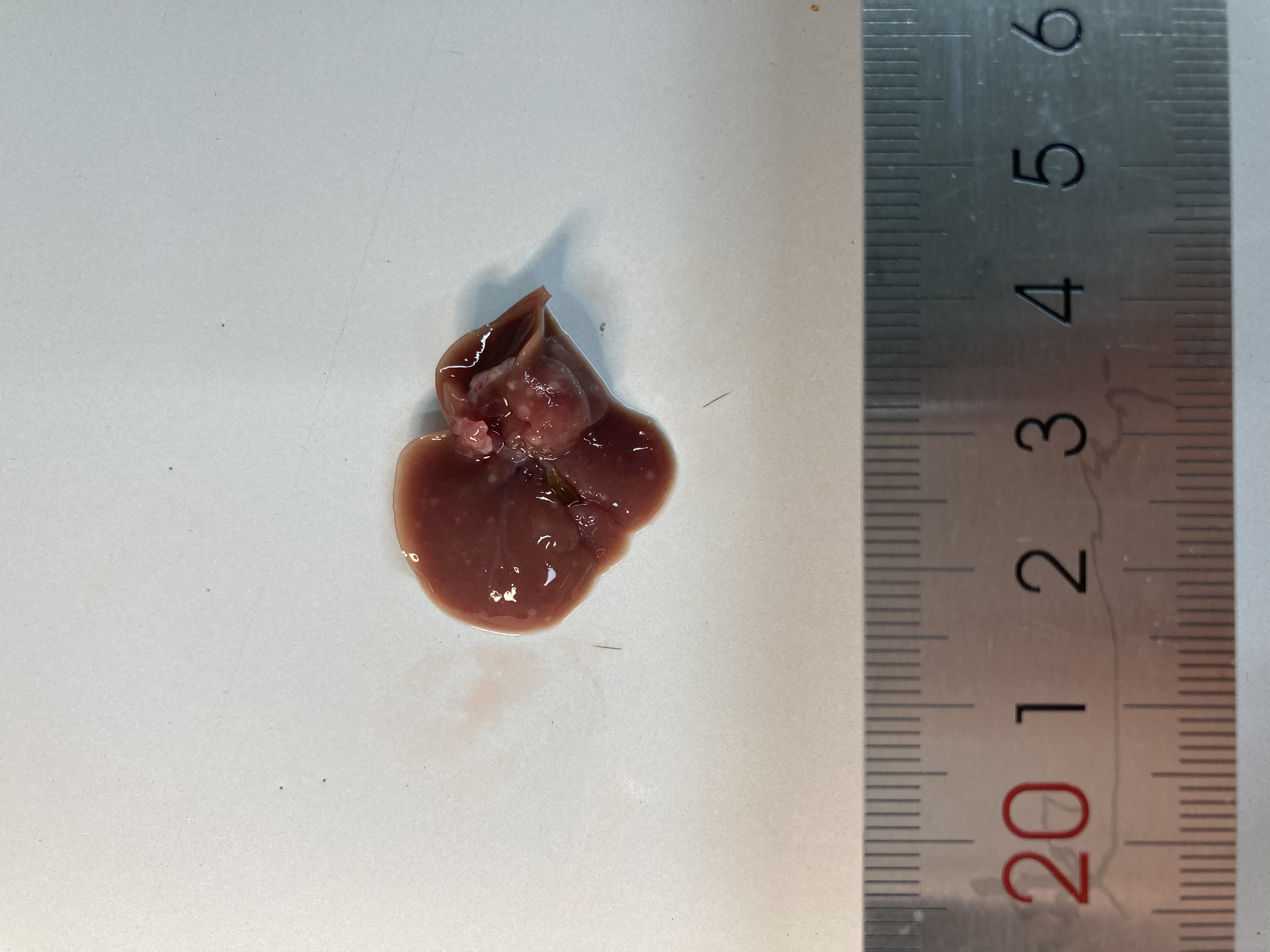

Supplement: Supplementary file 5 [file DataSheet6.ZIP › mouse liver of Combination Group/Combination7.jpg]

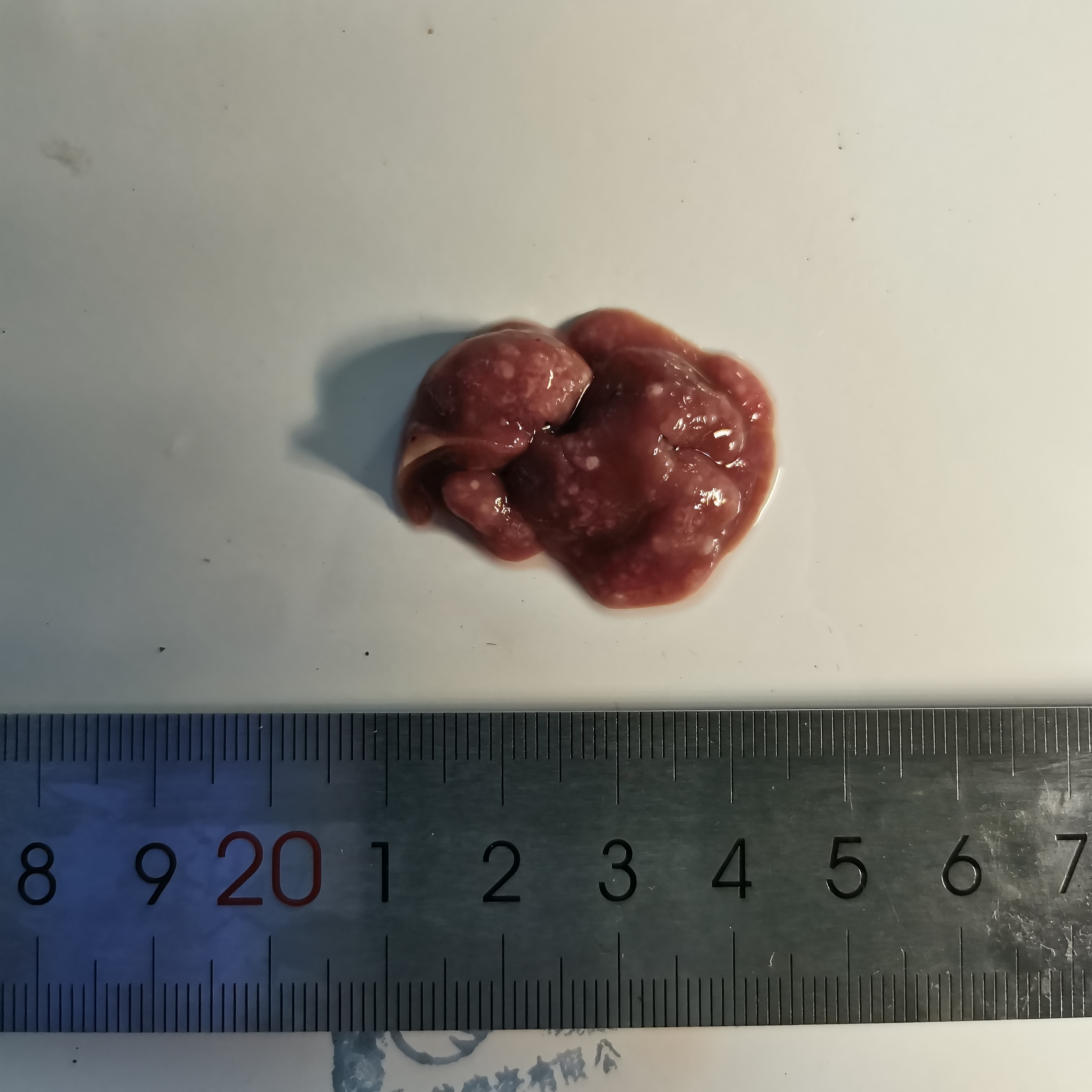

Supplement: Supplementary file 8 [file DataSheet5.ZIP › mouse liver of Anti-PD1 Group/Anti-PD1 1.jpg]

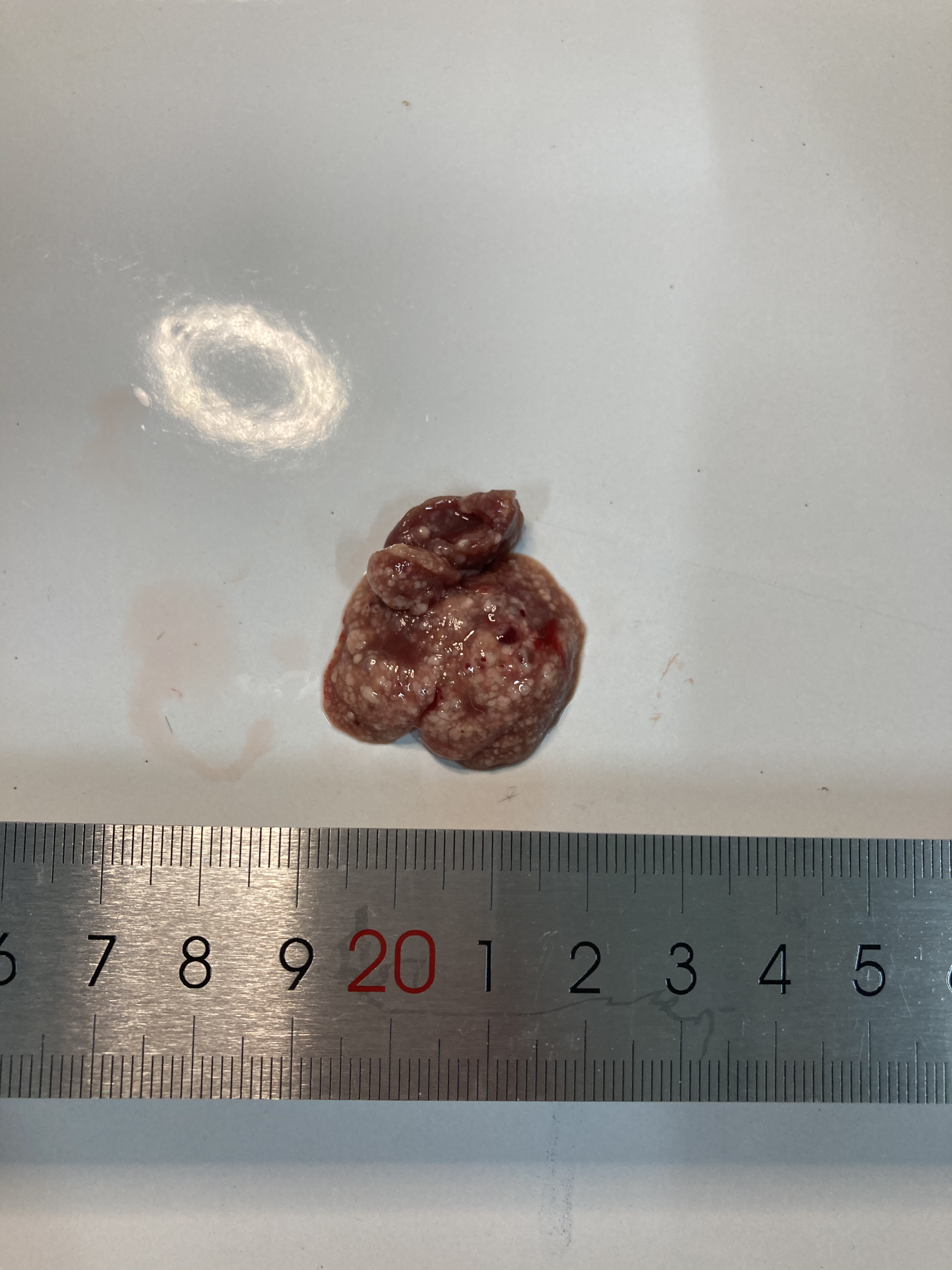

Supplement: Supplementary file 8 [file DataSheet5.ZIP › mouse liver of Anti-PD1 Group/Anti-PD1 2.jpg]

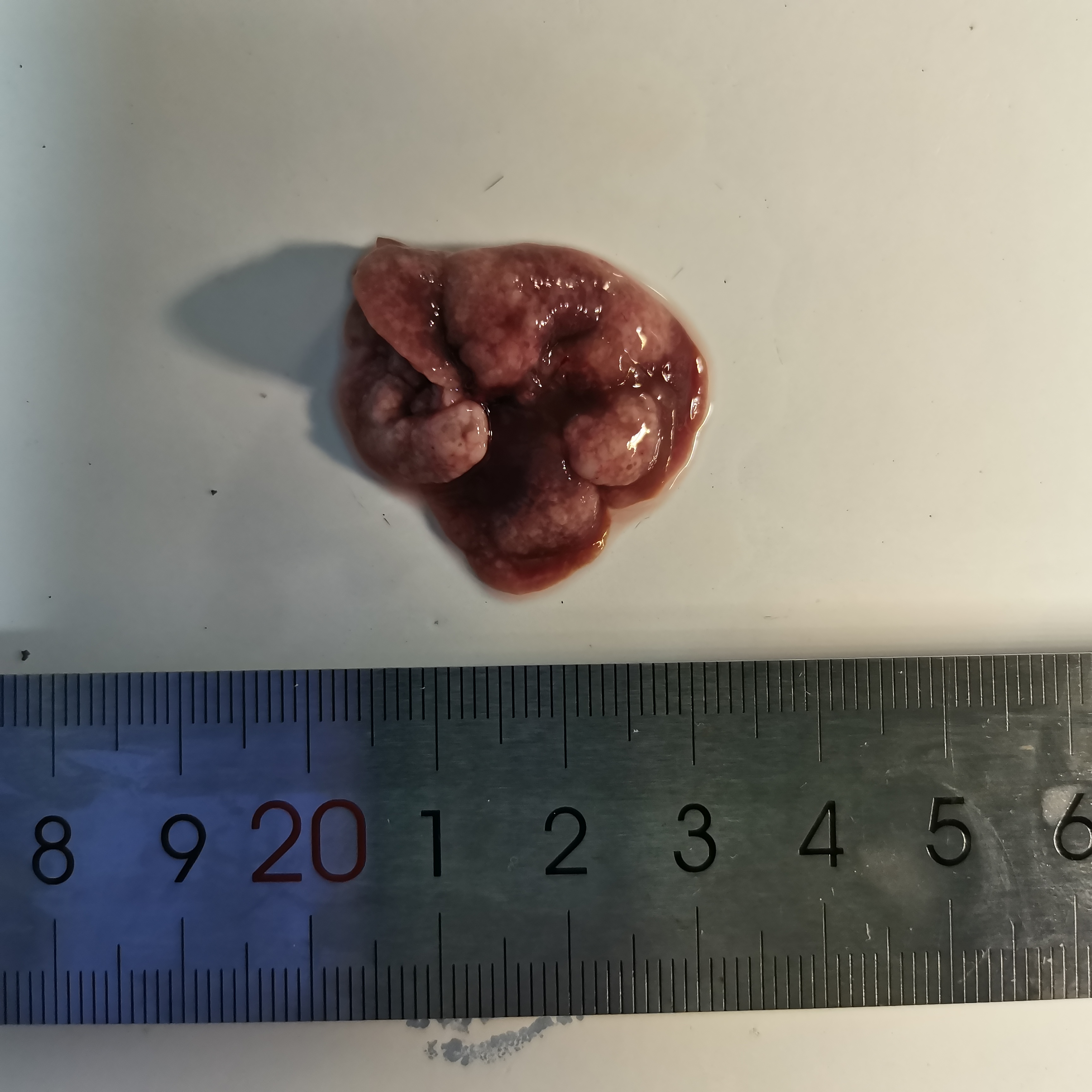

Supplement: Supplementary file 8 [file DataSheet5.ZIP › mouse liver of Anti-PD1 Group/Anti-PD1 3.jpg]

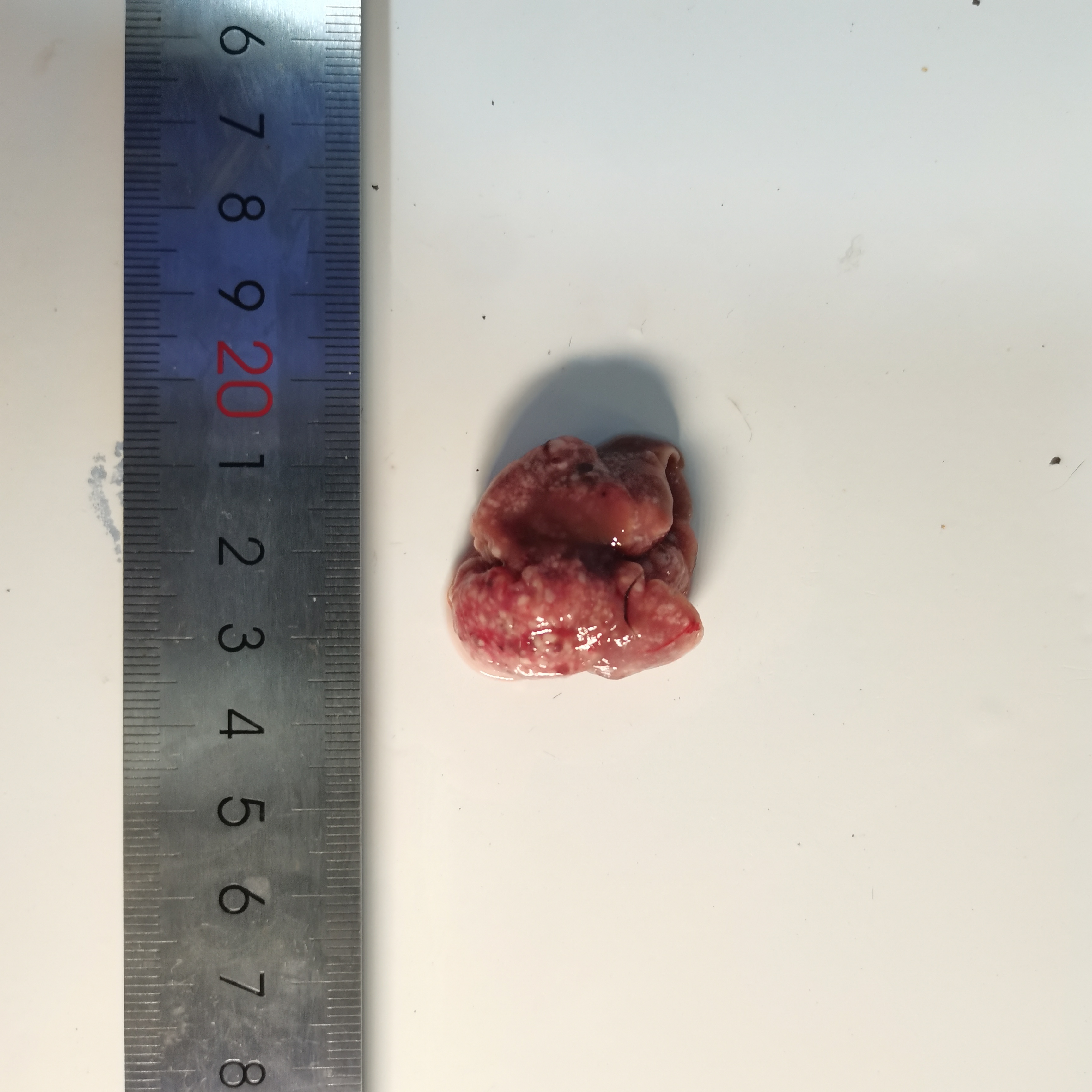

Supplement: Supplementary file 8 [file DataSheet5.ZIP › mouse liver of Anti-PD1 Group/Anti-PD1 4.jpg]

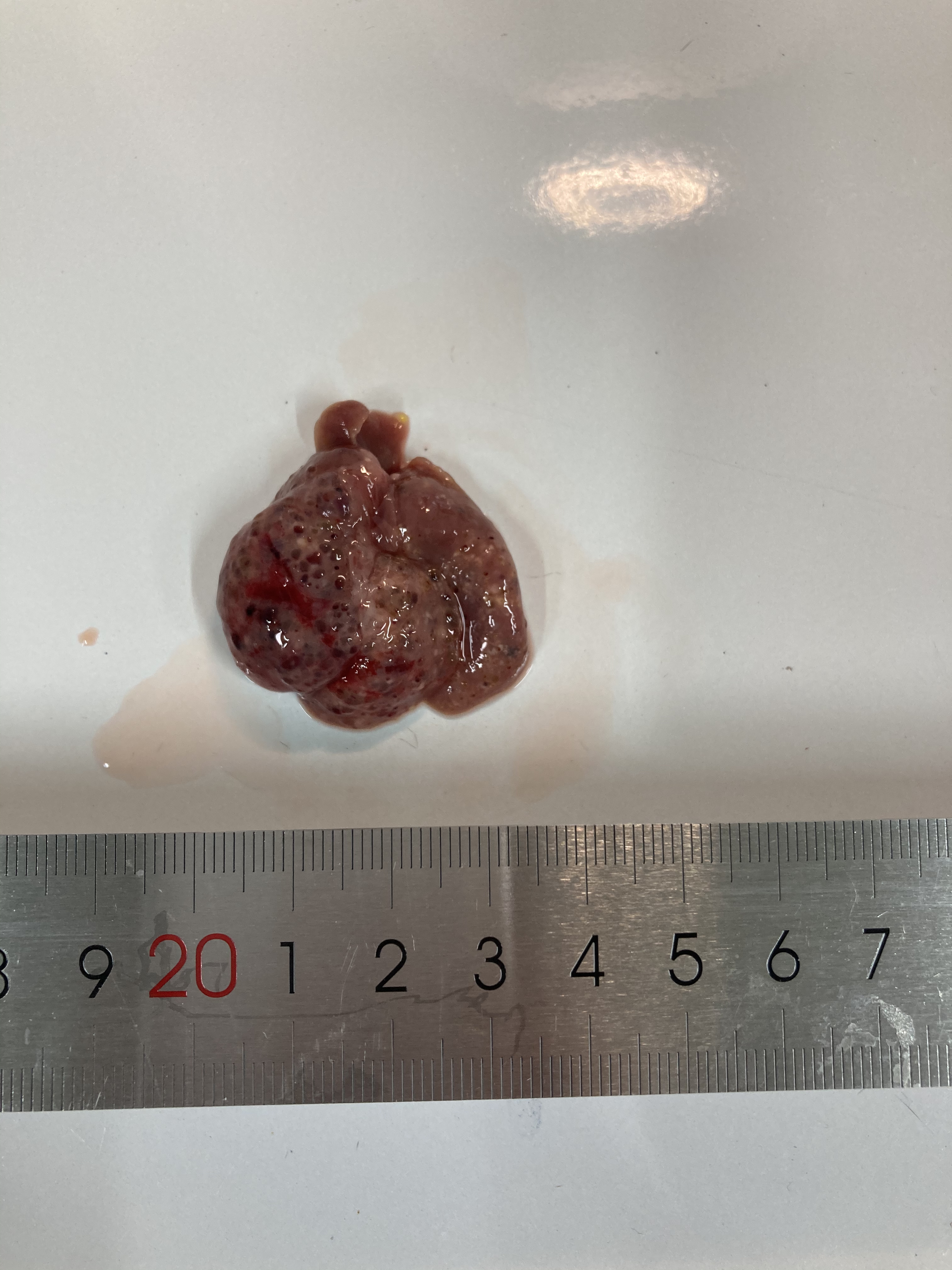

Supplement: Supplementary file 8 [file DataSheet5.ZIP › mouse liver of Anti-PD1 Group/Anti-PD1 5 .jpg]

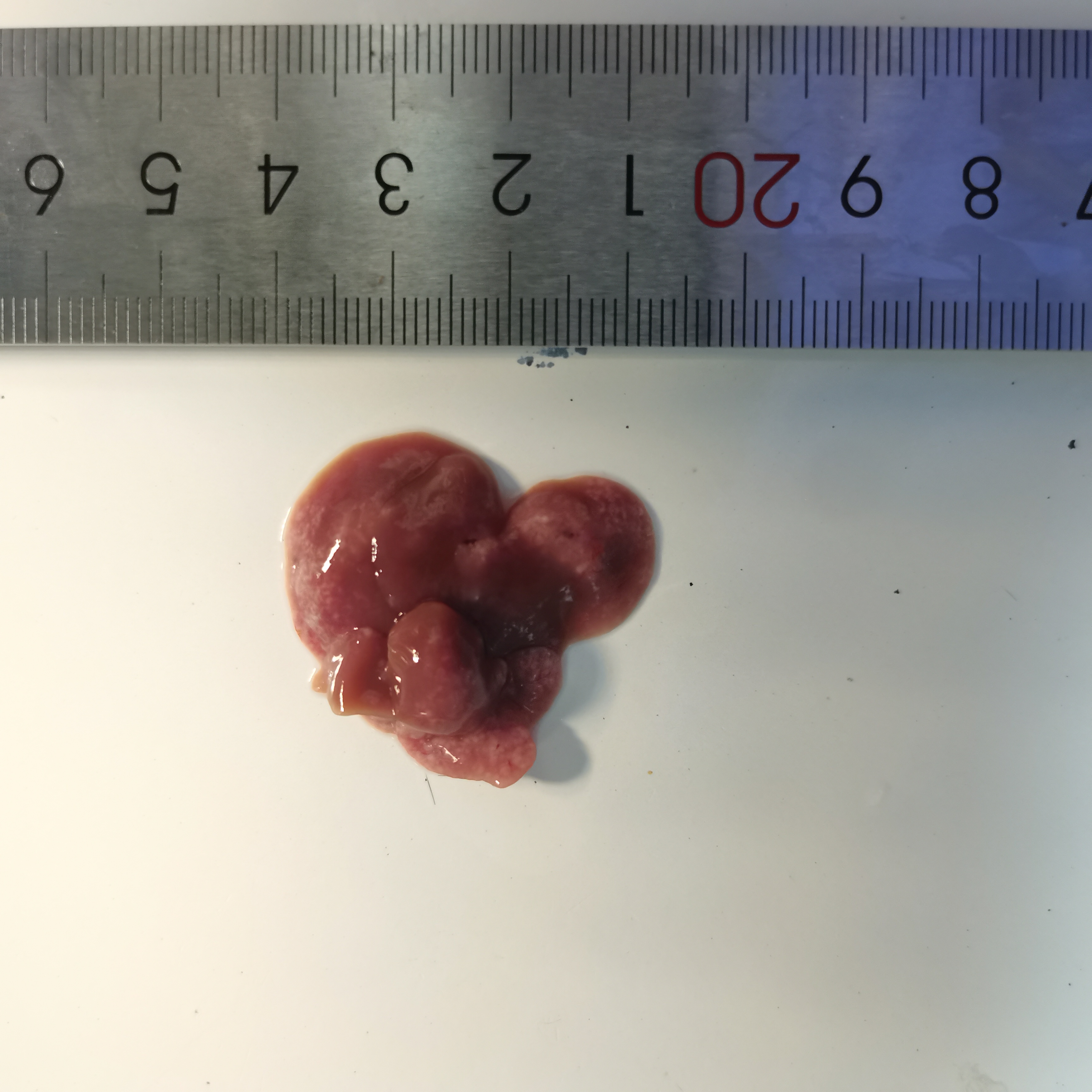

Supplement: Supplementary file 8 [file DataSheet5.ZIP › mouse liver of Anti-PD1 Group/Anti-PD1 6 .jpg]

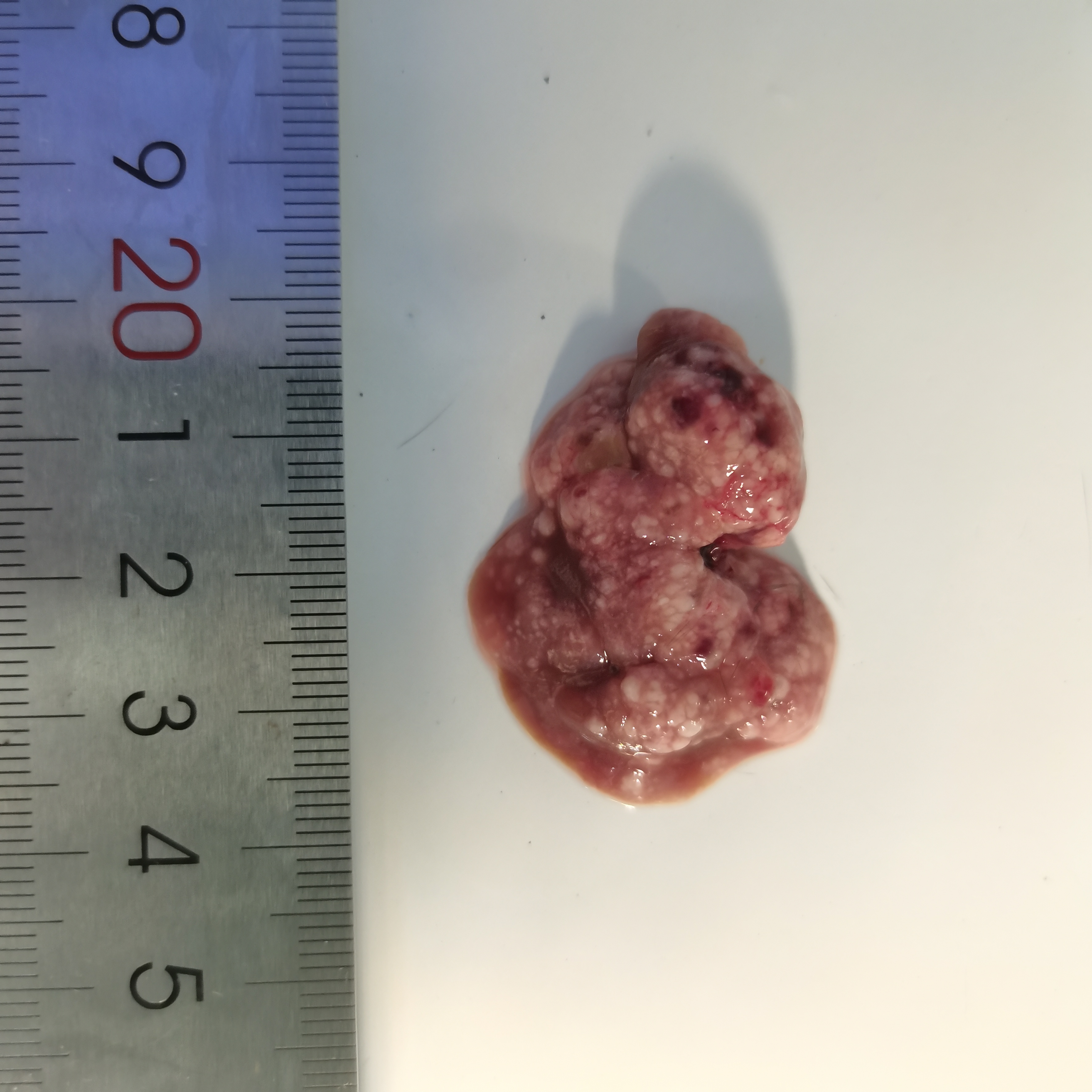

Supplement: Supplementary file 8 [file DataSheet5.ZIP › mouse liver of Anti-PD1 Group/Anti-PD1 7.jpg]

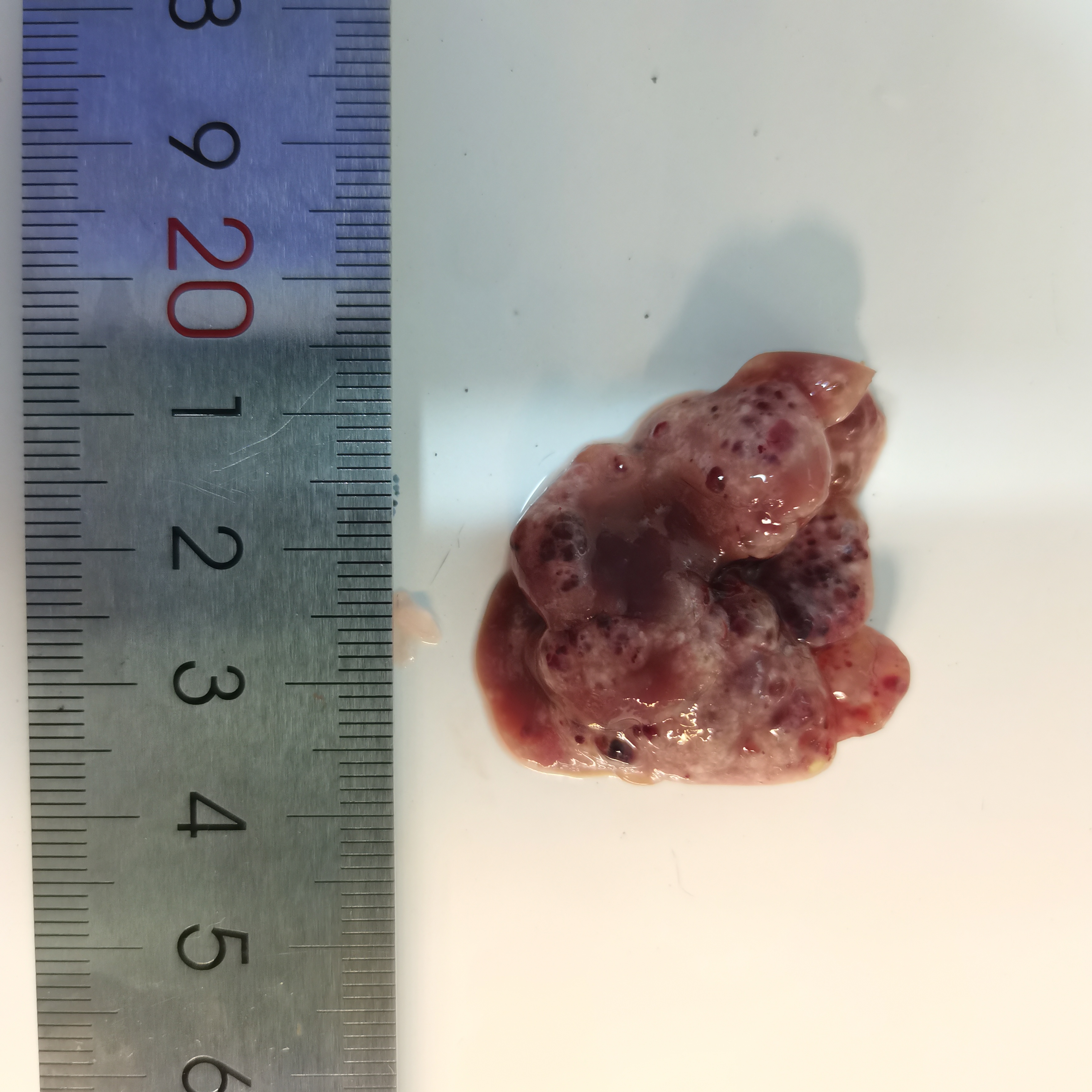

Supplement: Supplementary file 9 [file DataSheet7.ZIP › mouse liver of FAK inhibitor Group/FAK inhibitor1.jpg]

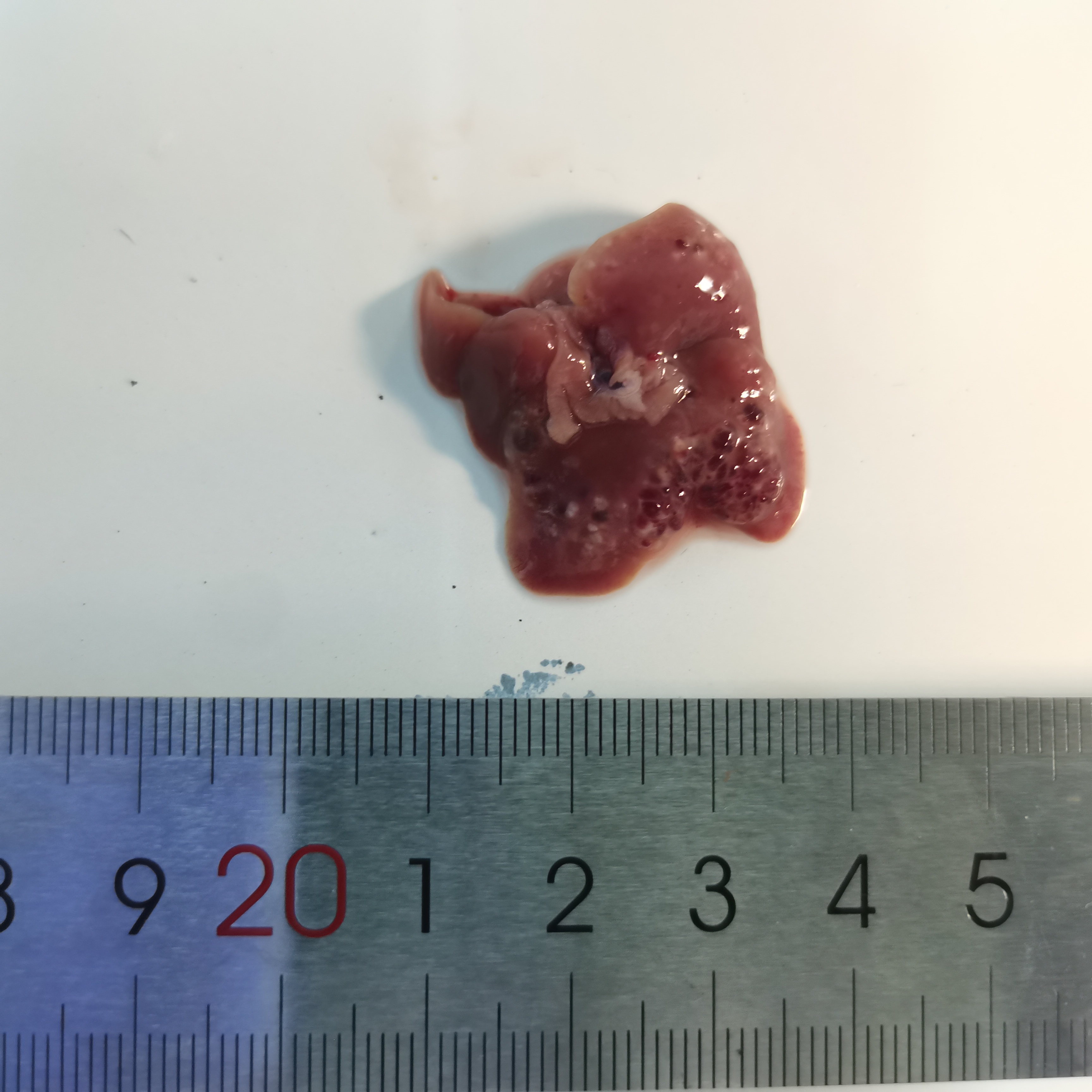

Supplement: Supplementary file 9 [file DataSheet7.ZIP › mouse liver of FAK inhibitor Group/FAK inhibitor2.jpg]

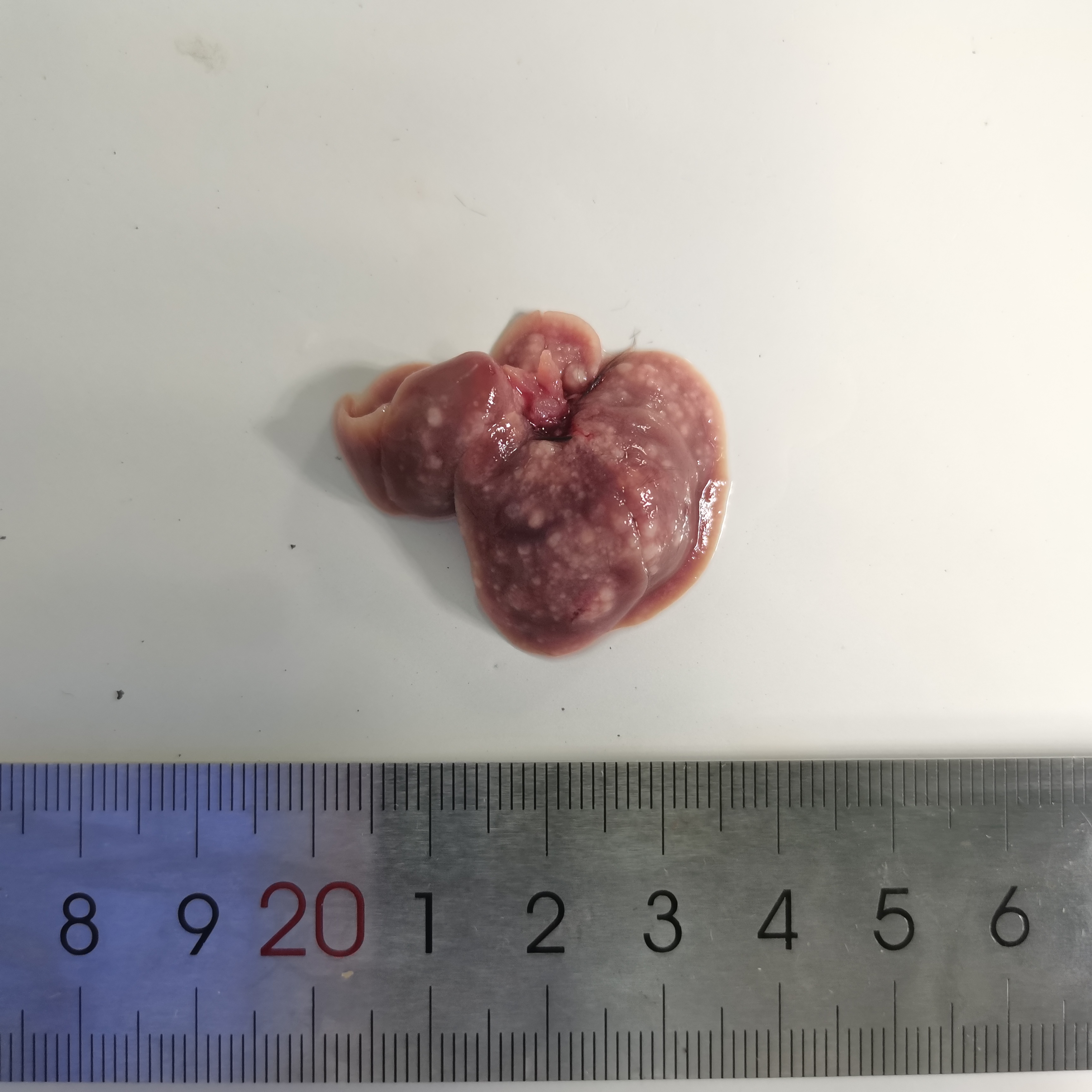

Supplement: Supplementary file 9 [file DataSheet7.ZIP › mouse liver of FAK inhibitor Group/FAK inhibitor3.jpg]

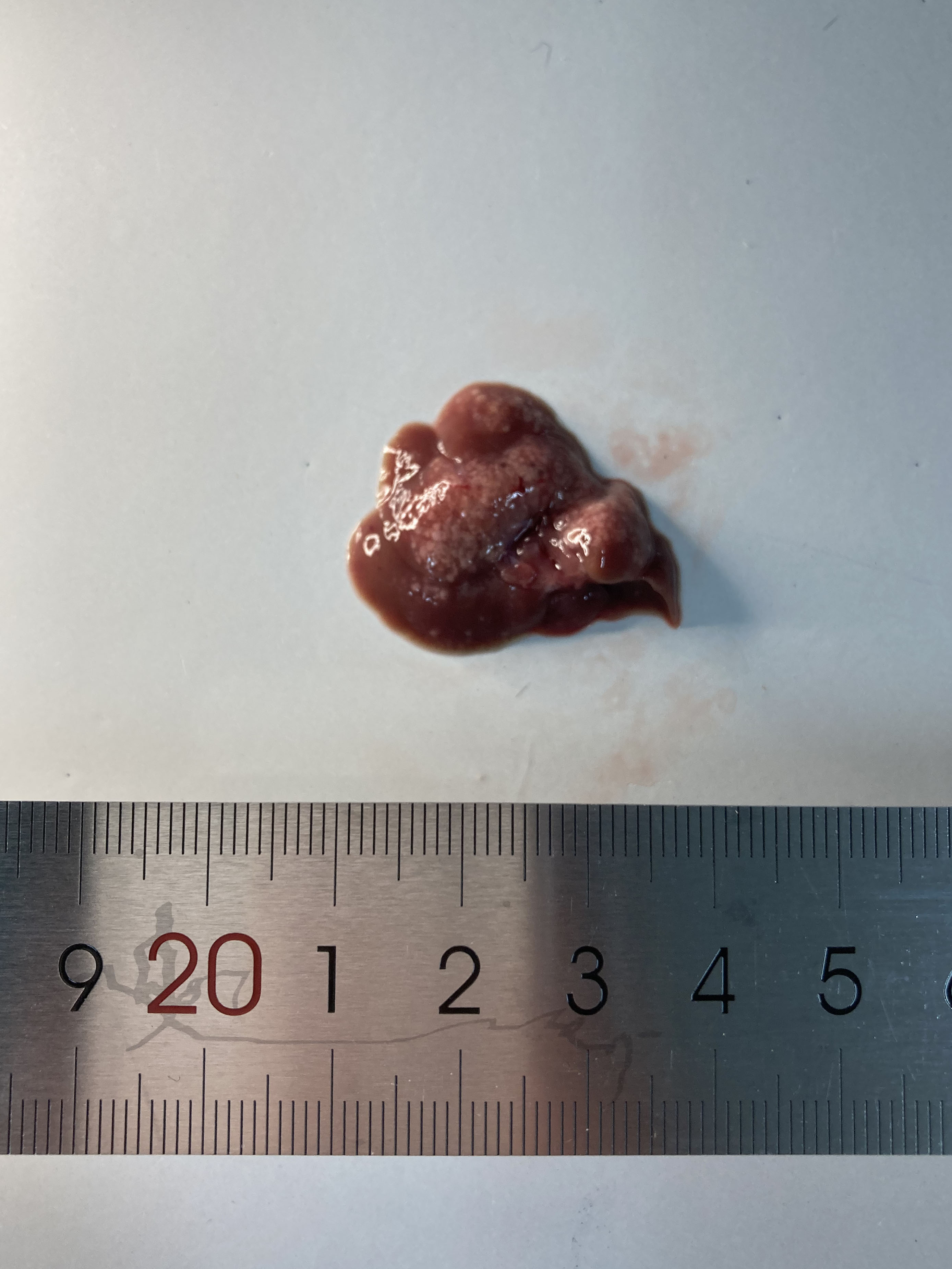

Supplement: Supplementary file 9 [file DataSheet7.ZIP › mouse liver of FAK inhibitor Group/FAK inhibitor4.jpg]

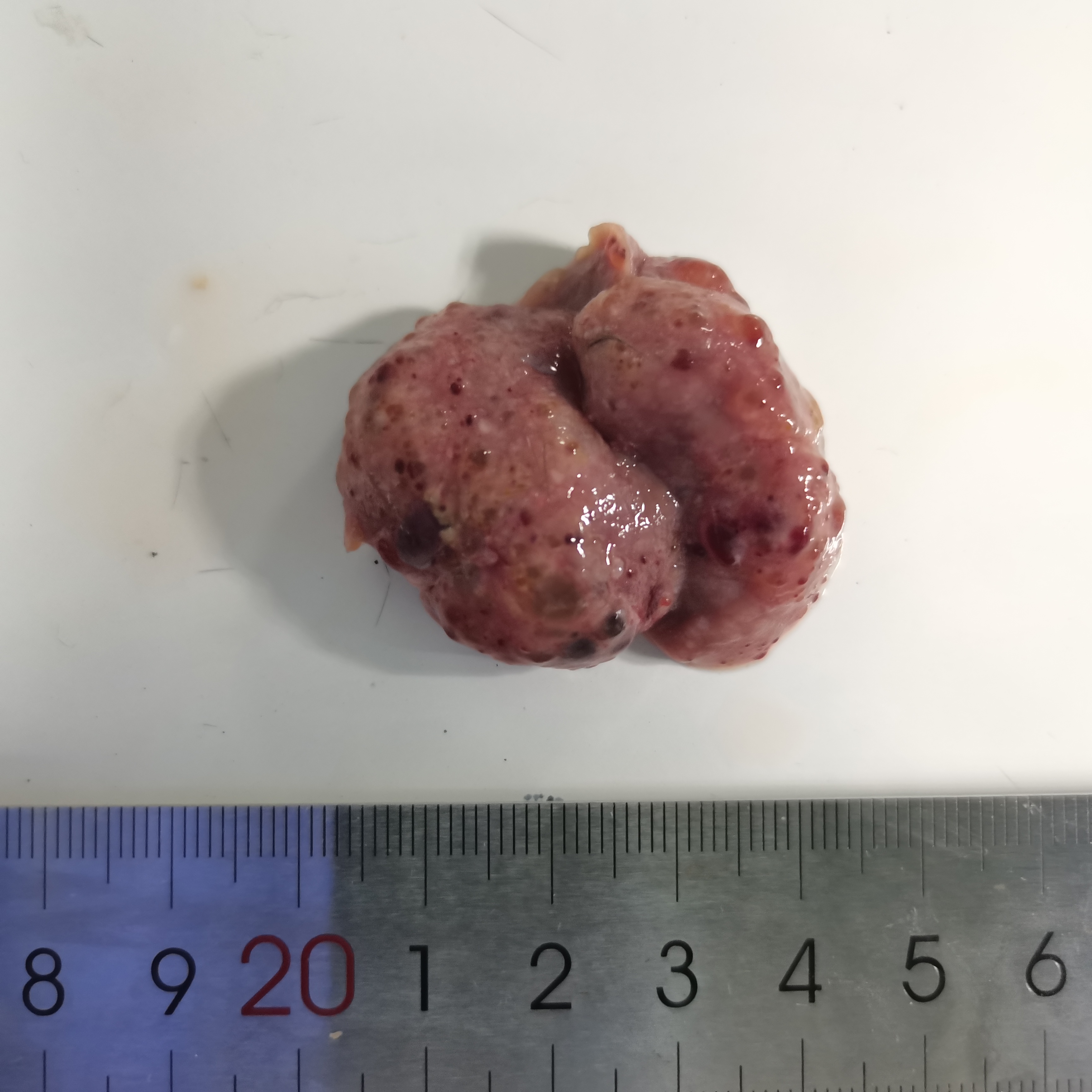

Supplement: Supplementary file 9 [file DataSheet7.ZIP › mouse liver of FAK inhibitor Group/FAK inhibitor5.jpg]

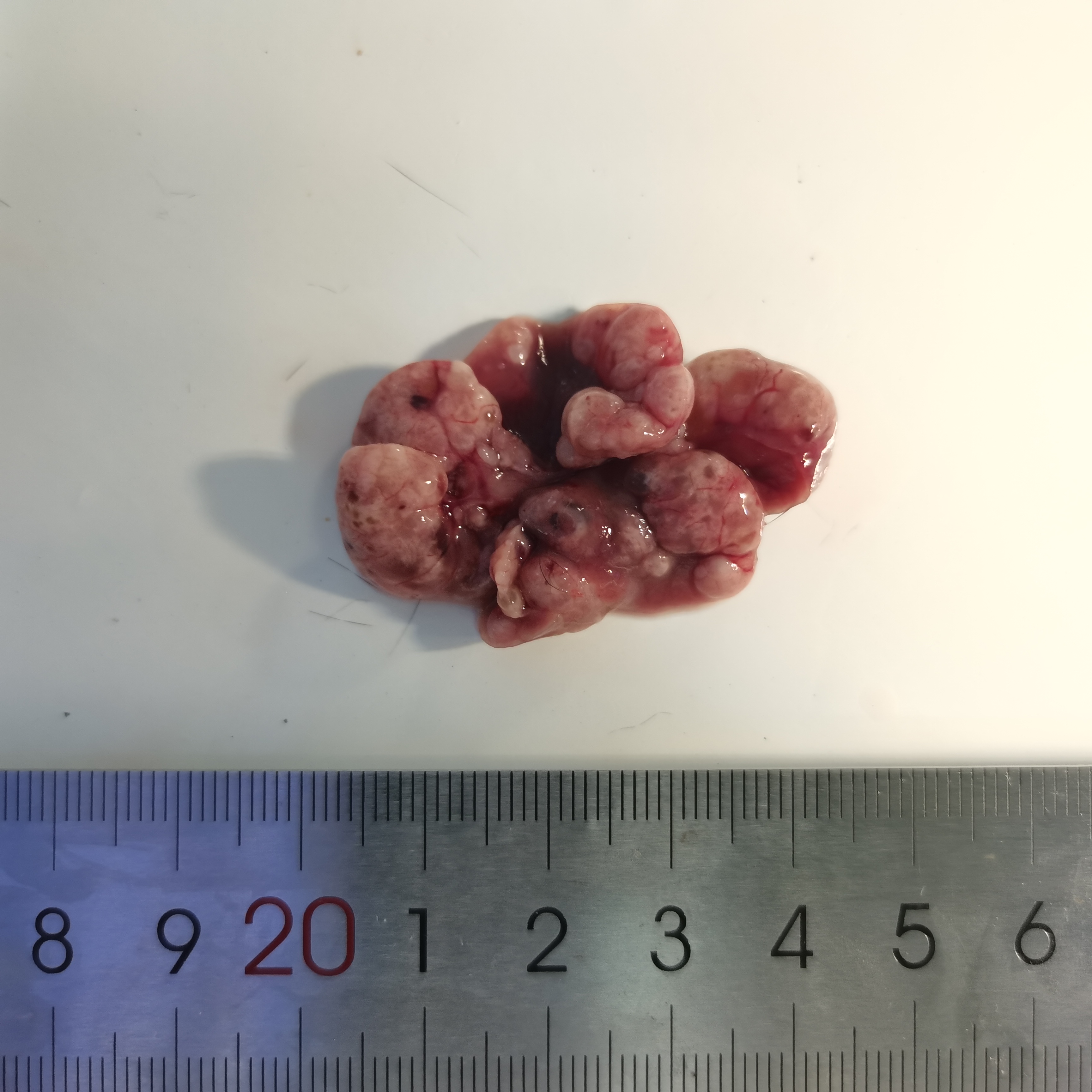

Supplement: Supplementary file 9 [file DataSheet7.ZIP › mouse liver of FAK inhibitor Group/FAK inhibitor6.jpg]
