# Supplementary material for: A FAK Inhibitor Boosts Anti-PD1 Immunotherapy in a Hepatocellular Carcinoma Mouse Model
Source: Front Pharmacol. 2022 Jan 18;12:820446. doi: 10.3389/fphar.2021.820446 (PMC8804348; doi:10.3389/fphar.2021.820446)
Supplement: Supplementary file 3 [file Presentation1.PPTX]

## Slide 1
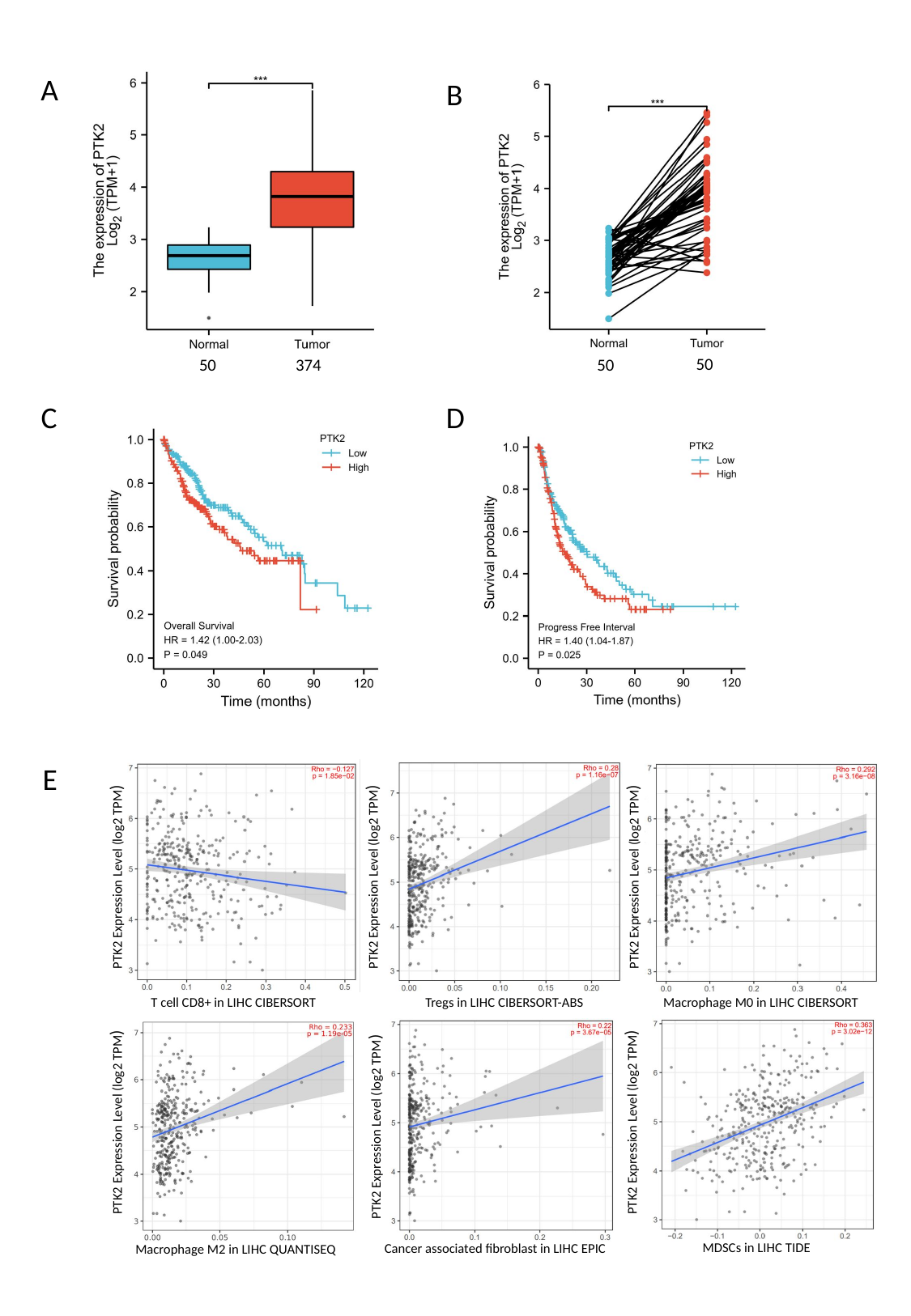

A
B
50
50
50
374
C
D
E
PTK2 Expression Level (log2 TPM)
PTK2 Expression Level (log2 TPM)
PTK2 Expression Level (log2 TPM)
T cell CD8+ in LIHC CIBERSORT
 Tregs in LIHC CIBERSORT-ABS
Macrophage M0 in LIHC CIBERSORT
PTK2 Expression Level (log2 TPM)
PTK2 Expression Level (log2 TPM)
PTK2 Expression Level (log2 TPM)
MDSCs in LIHC TIDE
Cancer associated fibroblast in LIHC EPIC
Macrophage M2 in LIHC QUANTISEQ

## Slide 2
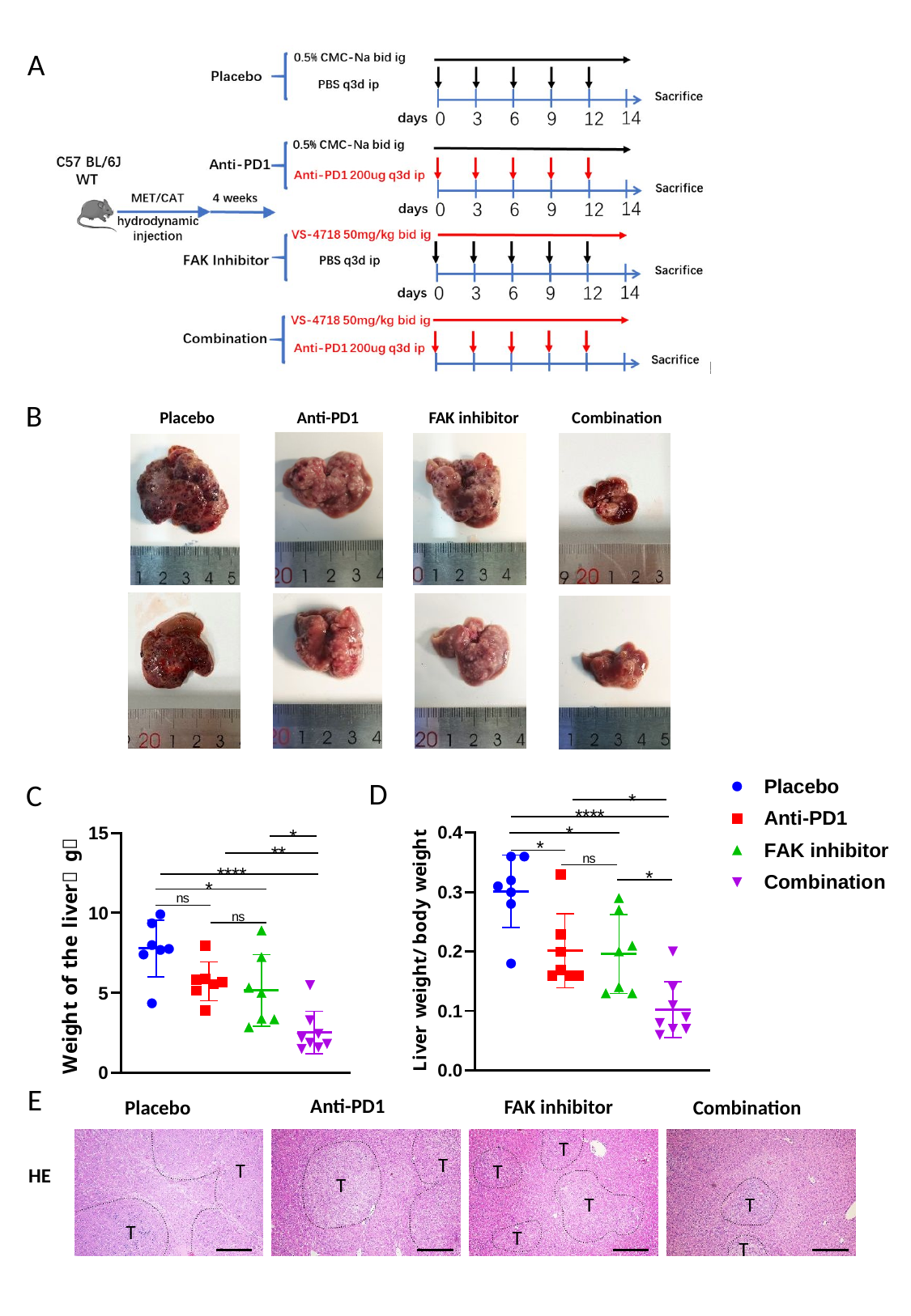

A
B
Anti-PD1
Placebo
FAK inhibitor
Combination
D
C
E
Anti-PD1
FAK inhibitor
Combination
Placebo
T
T
T
T
HE
T
T
T
T
T
T

## Slide 3
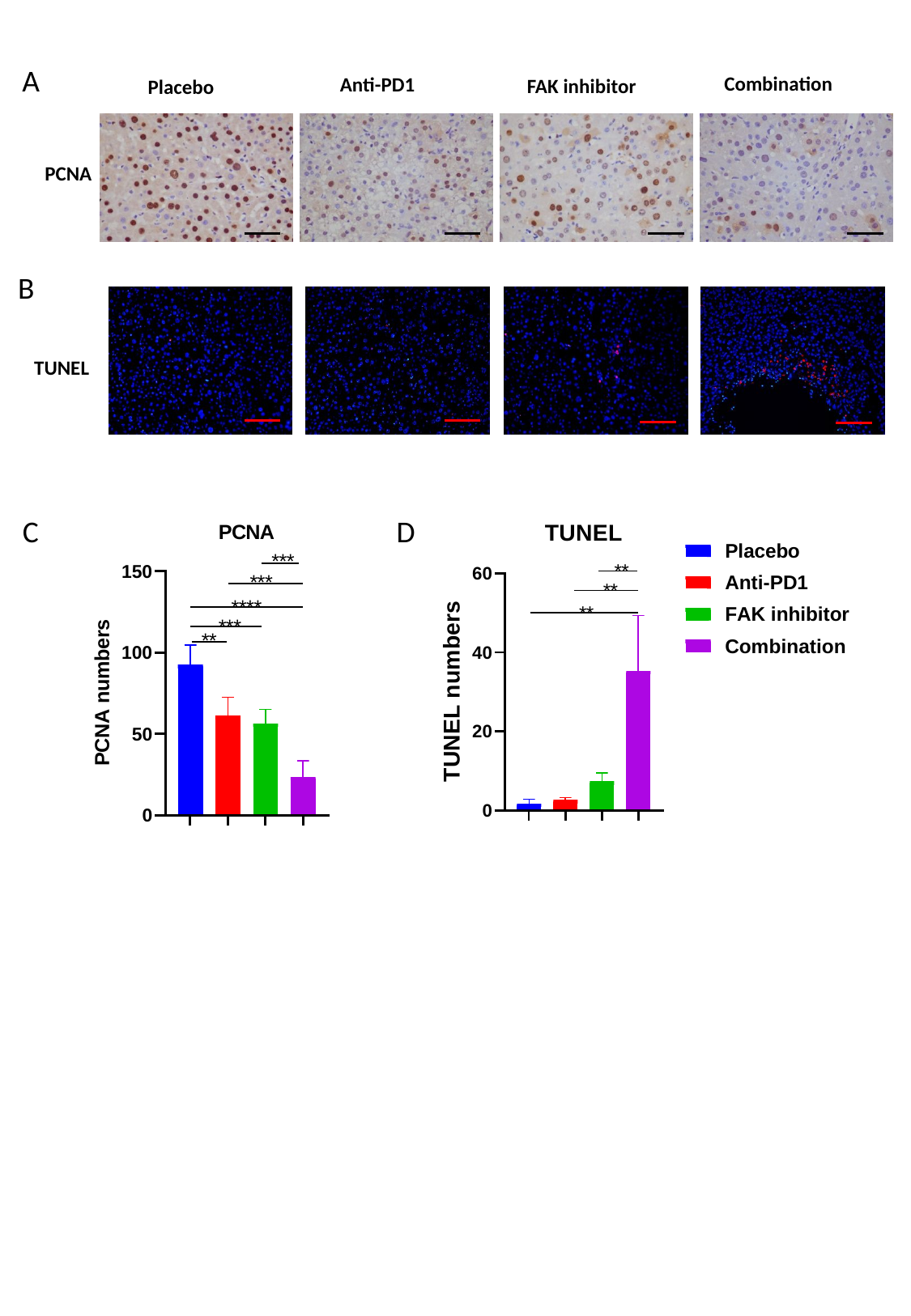

A
Combination
Anti-PD1
FAK inhibitor
Placebo
PCNA
B
TUNEL
C
D

## Slide 4
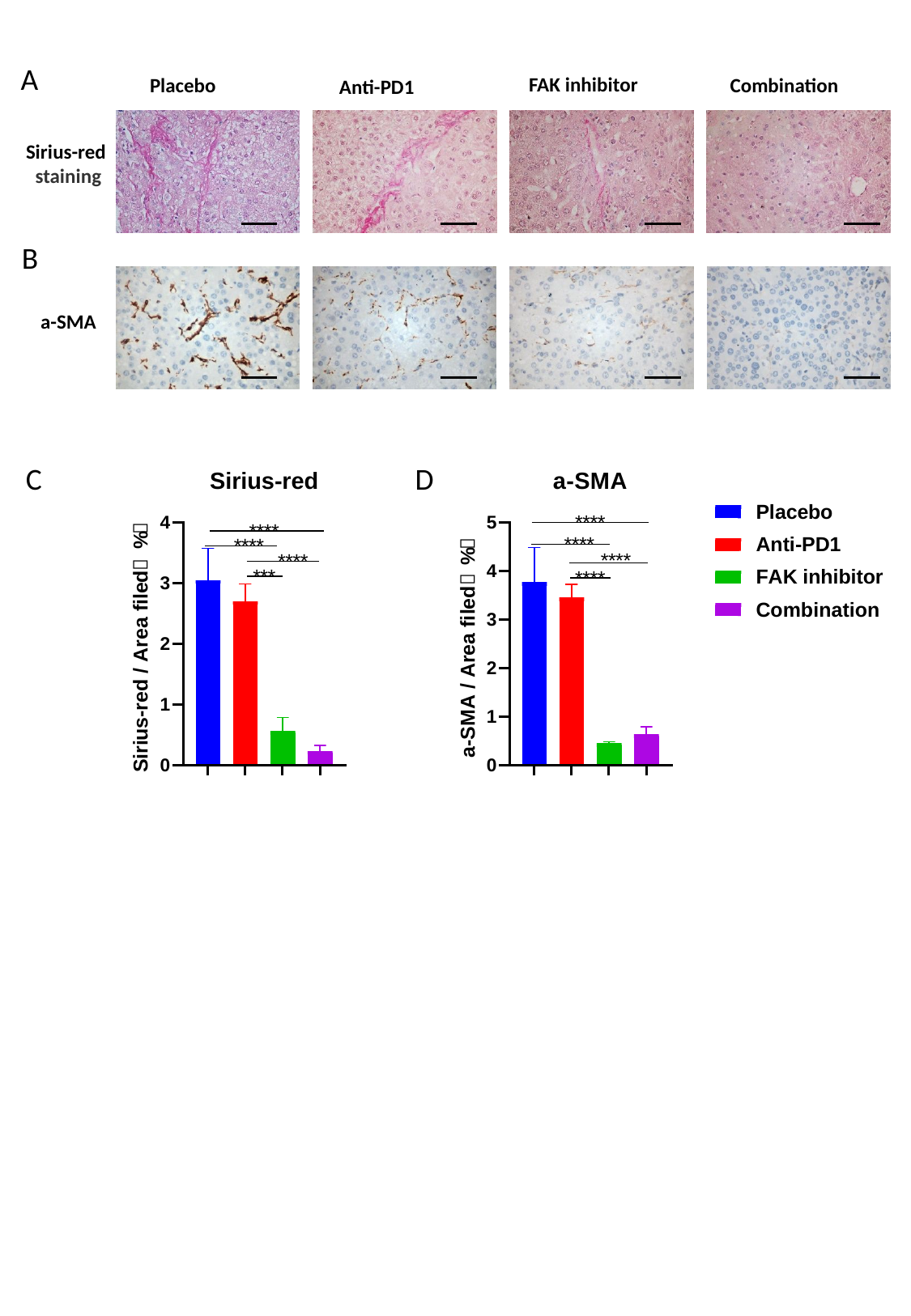

A
FAK inhibitor
Placebo
Combination
Anti-PD1
Sirius-red
staining
B
a-SMA
C
D

## Slide 5
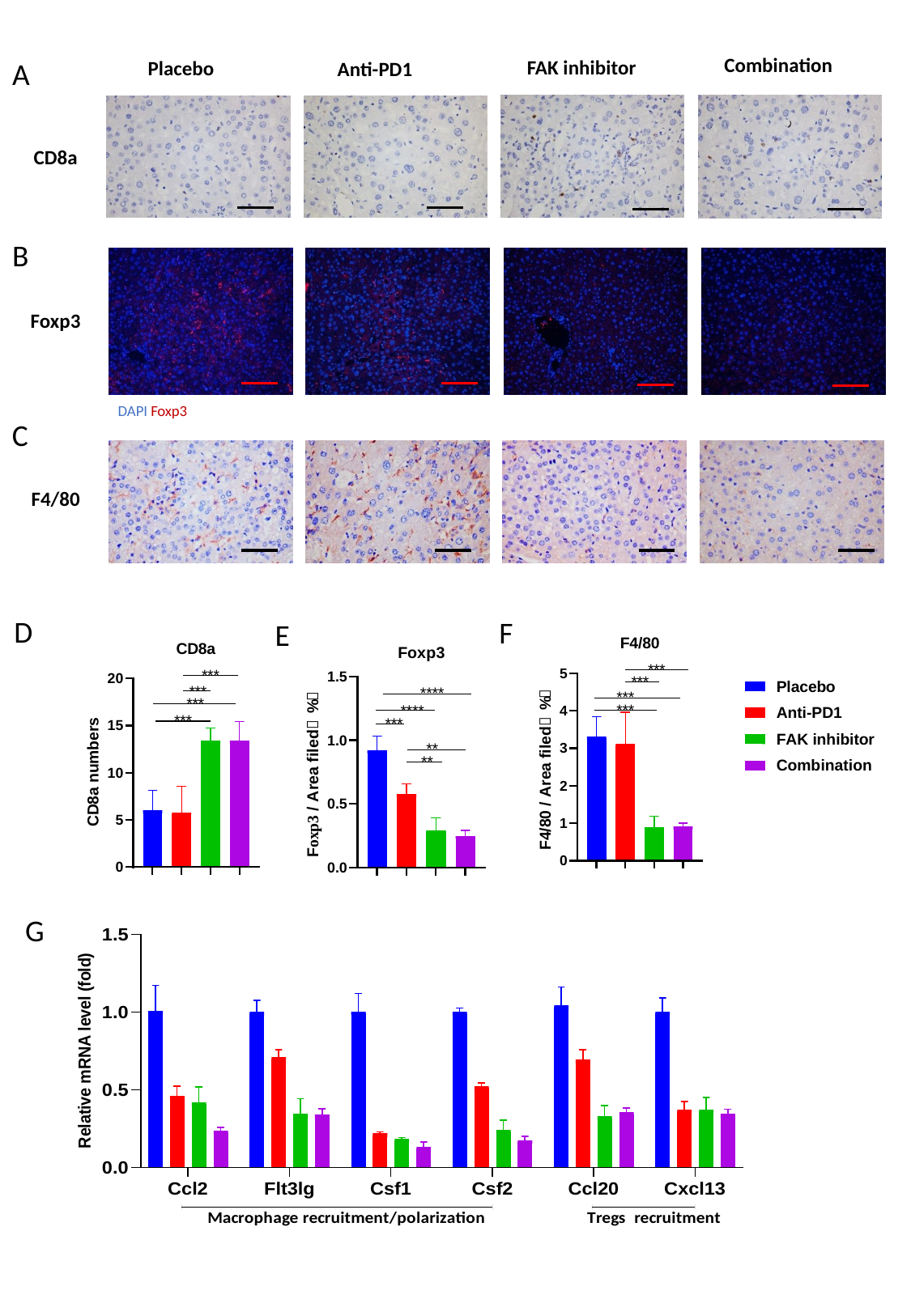

Combination
FAK inhibitor
Placebo
A
Anti-PD1
CD8a
B
Foxp3
DAPI Foxp3
C
F4/80
D
F
E
G

## Slide 6
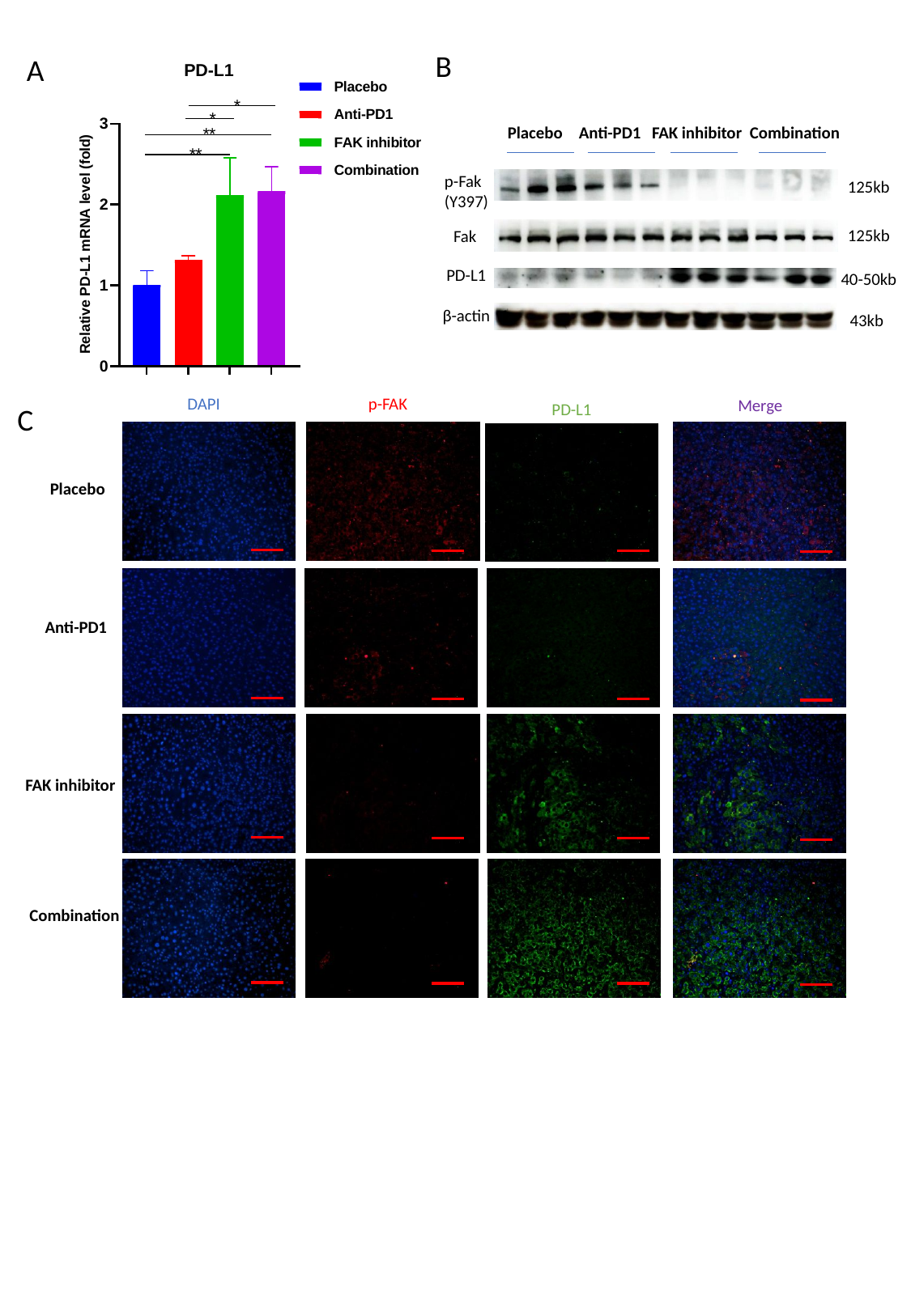

B
A
Anti-PD1
FAK inhibitor
Placebo
Combination
p-Fak
(Y397)
125kb
125kb
Fak
PD-L1
40-50kb
β-actin
43kb
DAPI
p-FAK
Merge
PD-L1
C
Placebo
Anti-PD1
FAK inhibitor
Combination
